# Global, regional, and national burden and trends of syphilis among women of childbearing age from 1990 to 2021

**DOI:** 10.3389/fpubh.2025.1580964

**Published:** 2025-07-08

**Authors:** Yanhui Huang, Yunfeng Ye, Limei Li, Zhiheng Zhou

**Affiliations:** ^1^Public Health Service Center, Bao’an District, Shenzhen, China; ^2^Chronic Disease Prevention and Control Hospital of Bao’an District, Shenzhen, China; ^3^Pingshan Hospital of Southern Medical University, Shenzhen, China

**Keywords:** syphilis, women of childbearing age, burden of disease, estimated annual percentage change, age-standardized rate

## Abstract

**Background:**

Syphilis represents a significant sexual health concern for women of childbearing age (WCBA) worldwide. However, information regarded the burden and trends associated with this disease is limited. This study aimed to evaluate the changes in syphilis burden among WCBA aged 15–49 years from 1990 to 2021 at global, regional, and national levels.

**Methods:**

The extensive information was gathered from the Global Burden of Disease (GBD) 2021 database concerning the incidence, prevalence, and disability-adjusted life years (DALYs) related to syphilis in WCBA aged 15–49 across 204 countries and territories from 1990 to 2021. To quantify temporal trends, the estimated annual percentage change (EAPC) was calculated in age-standardized rate (ASR) for incidence, prevalence, and DALYs based on age group, region, and sociodemographic index (SDI). The relationship between ASR and SDI was examined using spearman correlation analysis.

**Results:**

In 2021, there were 20.48 million prevalent cases, 5.36 million new syphilis cases, and 39.59 thousand DALYs among WCBA, reflecting increases of 45.85, 46.96, and 16.08%, respectively, since 1990. Over 32 years, global rates of prevalence, incidence, and DALYs declined, with EAPCs of −0.50, −0.35, and −1.30. However, high-middle SDI regions experienced rising trends in incidence (EAPC: 0.28) and prevalence (EAPC: 0.22). The Low-middle SDI region had the highest syphilis cases among WCBA, accounting for about one-third of the global total. Notably, the 20–24 age group had the highest incidence rate at 467.35 per 100,000.

**Conclusion:**

Our findings highlight a decline in the global prevalence of syphilis from 1990 to 2021, the burden of this disease remains significant in low-and middle-income countries and regions. The development of more effective strategies to prevent and reduce the burden of syphilis is a pressing need.

## 1 Introduction

Syphilis, a chronic and multistage disease engendered by the bacterium *Treponema pallidum* (1), is transmitted via sexual contact, blood transfusions, and from mother to child. This sexually transmitted infection (STI) poses a substantial and enduring challenge to global public health, particularly affecting women of childbearing age (WCBA) (2). The disease exerts significant effects on various bodily systems beyond the reproductive organs, including the nervous system, cardiovascular system, ocular structures, and hepatic function (3). Furthermore, among women aged 15–49, syphilis stands as a principal contributor to reproductive tract diseases, encompassing infertility and pelvic inflammatory diseases. It can also precipitate severe outcomes during pregnancy, such as neonatal mortality, congenital anomalies, and other adverse pregnancy outcomes (APOs), with a particularly pronounced impact in low-and middle-income countries (4, 5).

Globally, it is estimated that there are approximately 12 million new cases of syphilis annually, with 90% occurring in developing countries (6). Among WCBA in the aged 15–49 years, the World Health Organization (WHO) estimates that about 4 million adults were infected with syphilis in 2022 (7). This age group is significant as it includes the majority of the reproductive-age population, and infection rates among women in this age bracket are key indicators of the disease’s impact on fertility and pregnancy outcomes. Regional trends show a concerning rise in syphilis cases in certain areas, such as Japan, where the number of cases has been increasing since 2013, reaching a record high of 14,906 cases in 2023, and 10,162 new cases as of September 15, 2024, indicating an alarming upward trend (8). This resurgence in Japan highlights the need for vigilant surveillance and intervention strategies to control the spread of the disease (9).

The WHO has established an objective to achieve a 90% reduction in global syphilis incidence by 2030, alongside a 50% decrease in congenital syphilis cases per 100,000 live births across 80% of affected nations (10, 11). However, recent data indicate an increase in the global prevalence of syphilis, which underscores the urgency for countries to revise their policies and focus on high-risk populations to achieve this target (12). Initiatives such as the Syphilis Interventions Towards Elimination (SITE) model, implemented in countries like Papua New Guinea, have shown that scaling up clinical treatment, contact tracing, and targeted behavioral risk reduction interventions can significantly reduce the syphilis burden (1, 13). Additionally, the Eliminating Mother-to-Child Transmission (EMTCT) of Syphilis initiative has demonstrated significant progress in countries like China, where the congenital syphilis incidence rate per 100,000 live births has markedly decreased from 69.9 in 2013 to 11.9 in 2019 (14). These strategies offer valuable insights for nations seeking to implement effective syphilis control measures (15).

The significance of this research lies in its concentration on the demographic group known as WCBA, who are especially susceptible to the detrimental effects of syphilis. Comprehending the magnitude and evolving trends of syphilis within this demographic is essential for guiding public health policies and interventions designed to mitigate the disease’s influence. Consequently, it is imperative to furnish a detailed depiction and examination of the comprehensive disease profile and shifting trends of syphilis affecting WCBA. Utilizing the most recent data from the Global Burden of Disease (GBD) Study 2021, we undertook an analysis of syphilis incidence, prevalence, and disability-adjusted life years (DALYs) among WCBA, spanning global, regional, and national scopes from 1990 to 2021, and compared the distribution and alterations in the syphilis burden across various age cohorts.

## 2 Methods

### 2.1 Data sources and disease definition

The data pertaining to syphilis among WCBA employed in this analysis were procured from the GBD Study 2021. This comprehensive dataset provides the latest estimates on the epidemiological impact of 371 diseases and injuries across 21 GBD regions and 204 countries and territories, encompassing the period from 1990 to 2021. This information is publicly available through the Global Health Data Exchange^1^ (16), with extensive details on the methodologies and statistical approaches provided in prior reports (17). The datasets incorporated a variety of sources, including vital registration systems, verbal autopsies, census data, household surveys, disease-specific registries, and health service utilization records (18). A diagnosis of syphilis was established when a patient’s clinical presentation and laboratory findings align with the criteria set forth by the Centers for Disease Control and Prevention and the WHO. According to the International Classification of Diseases (ICD), both the 9th and 10th editions categorize syphilis under codes “090–097.9” and “A50-A53.9, I98.0, K67.2, M73.1-M73.8,” respectively (19). Further details on data selection were shown in Supplementary Methods and Supplementary Figure S1.

### 2.2 Sociodemographic index and regions

The sociodemographic index (SDI), introduced by the Institute for Health Metrics and Evaluation (IHME) in 2015, serves as a comprehensive indicator to assess the development level of countries or regions, highlighting the interconnection between social development and population health outcomes (16). Specifically, the SDI is calculated as the geometric mean of a 0 to 1 index that includes the total fertility rate for individuals younger than 25 years, mean education for those aged 15 years and older, and lag-distributed income per capita (20). In the GBD Study 2021, SDI values were scaled by a factor of 100 to establish a range from 0 to 100. This scale represents a continuum where 0 corresponds to the lowest levels of income and educational attainment coupled with the highest rates of fertility, and 100 corresponds to the highest levels of income and educational attainment and the lowest rates of fertility. Consequently, the 204 countries and territories were categorized into five distinct SDI quintiles: low, low-middle, middle, high-middle, and high. Additionally, these countries or territories were arranged into 21 regions based on epidemiological similarities and geographical proximity, such as the high-income Asia Pacific and Central Asia regions. This regional classification aims to enhance the nuanced comprehension of global health trends within the context of the GBD study (21).

### 2.3 Statistical analysis

We conducted an assessment of the age-standardized rate (ASR) pertaining to the prevalence, incidence, and DALYs for syphilis among WCBA from 1990 to 2021. The assessment was executed employing the global standard population as delineated in the GBD Study 2021 (22), along with their corresponding 95% confidence intervals (CIs) (23). The analysis used the “epitools” package in R software. By analyzing incidence, prevalence, DALYs, and age-standardized figures per 100,000 individuals, we outlined the global burden of syphilis while categorizing data by 204 countries, 21 geographic locations, SDI, and various age groups to provide a detailed perspective on how syphilis affects global health.

The estimated annual percentage change (EAPC) and 95% CI were calculated to assess the trend in ASR from 1990 to 2021, with the EAPC serving as a widely accepted summary measure for this purpose. A regression line was fitted to the natural log of the ASR, represented by the equation y = *α* + *β*x + ɛ, where y is the natural log of the ASR and x is the calendar year. The EAPC was derived as 100 × (exp(β) − 1) and its 95% CI was obtained from the linear regression model. An upward trend in ASR was indicated if both the EAPC and the lower limit of its 95% CI were positive, while a downward trend was identified if both the EAPC and the upper limit of its 95% CI were negative. If neither condition was met, the ASR was considered stable over the period.

The hierarchical clustering analysis was performed to classify countries and territories into three groups based on EAPC temporal trends in ASR of syphilis prevalence, incidence and DALYs: (a) significant decrease; (b) decrease; and (c) increase. The relationship between ASR and SDI was examined using spearman correlation analysis. In addition, to investigate the factors influencing EAPC, we examined the relationships between EAPC and ASR from 1990 and the SDI from 2021 at the national level. All statistical analyses were performed using R version 4.3.3, with a *p* value threshold of less than 0.05 considered statistically significant. Further details on analysis are shown in Supplementary Figure S1.

## 3 Results

### 3.1 Global trend of syphilis

**Figure 1:**
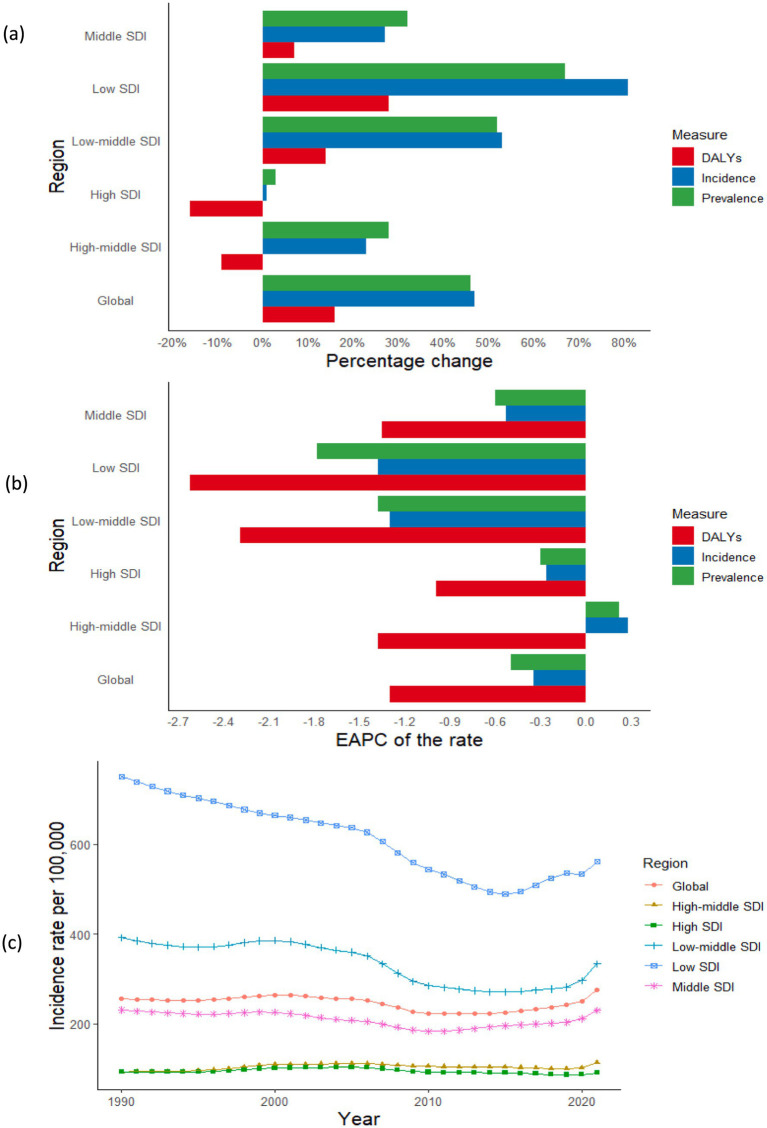
Temporal trend of syphilis burden in WCBA across 5 SDI regions: **(a)** Percentage change in cases of prevalent, incident, and DALYs in 1990 and 2021; **(b)** The EAPC of prevalence, incidence, and DALYs rates from 1990 to 2021; **(c)** The rates of incidence from 1990 to 2021. WCBA, women of childbearing age; EAPC, estimated annual percentage change; DALYs, disability-adjusted life years; SDI, sociodemographic index.

There has been a notable increase in the global incidence, prevalence, and DALYs associated with syphilis among WCBA. Specifically, the number of new cases surged from 3.64 million in 1990 to 5.36 million by 2021, reflecting a rise of 46.94%. Similarly, prevalent cases escalated from 14.04 million in 1990 to 20.48 million in 2021, representing a percentage change of 45.85%. Additionally, DALYs increased from approximately 34.11 thousand in 1990 to about 39.59 thousand in 2021, corresponding to a percentage change of 16.08% (Table 1; Figure 1a). In contrast, a decline was observed in the ASR for incidence, prevalence, and DALYs related to syphilis among WCBA globally from 1990 to 2021, with EAPCs recorded at −0.35 (95% CI: −0.56 to −0.15), −0.50 (95% CI: −0.69 to −0.31), and −1.30 (95% CI: −1.53 to −1.06), respectively. These findings indicate that the overall burden of syphilis among WCBA is decreasing worldwide (Table 1; Figures 1b,c; Supplementary Figure S3).

**Table 1:** Number of cases, age-standardized incidence, prevalence, and DALYs rates of syphilis among WCBA in 1990 and 2021; percentage change in prevalent, incident, and DALYs cases between 1990 and 2021; and EAPC of these rates from 1990 to 2021, by SID and GBD regions.

Items	1990		2021		1990–2021	
	Cases number	ASR per 100,000	Cases number	ASR per 100,000	EAPC	Cases change (%)
	No. (95% UI)	No. (95% CI)	No. (95% UI)	No. (95% CI)	No. (95% CI)	No. (95% UI)
*Incidence*						
Global	3,644,611 (2,528,551, 4,866,997)	257.38 (257.11, 257.65)	5,355,440 (3,717,326, 7,240,281)	275.45 (275.22, 275.69)	−0.35 (−0.56, −0.15)	46.94 (42.49, 50.86)
Sociodemographic index						
Low SDI	917,095 (658,897, 1,199,367)	750.74 (749.15, 752.32)	1,656,353 (1,160,830, 2,227,205)	561.61 (560.73, 562.49)	−1.38 (−1.56, −1.19)	80.61 (64.41, 93.17)
Low-middle SDI	1,143,508 (784,737, 1,531,944)	392.54 (391.80, 393.28)	1,749,377 (1,201,233, 2,371,568)	333.83 (333.33, 334.32)	−1.30 (−1.57, −1.02)	52.98 (48.00, 57.46)
Middle SDI	1,100,213 (749,009, 1,492,787)	231.28 (230.84, 231.73)	1,399,908 (979,372, 1,895,219)	230.45 (230.07, 230.83)	−0.53 (−0.74, −0.31)	27.24 (20.45, 33.65)
High-middle SDI	267,608 (185,166, 365,761)	93.55 (93.19, 93.91)	329,244 (231,640, 441,593)	113.60 (113.20, 114.00)	0.28 (0.08, 0.48)	23.03 (14.05, 32.20)
High SDI	213,100 (148,910, 290,712)	93.67 (93.27, 94.07)	216,230 (151,386, 293,382)	91.38 (90.99, 91.77)	−0.26 (−0.45, −0.08)	1.47 (−1.52, 5.00)
GBD regions						
High-income Asia Pacific	51,207 (35,953, 69,278)	113.69 (112.71, 114.69)	38,953 (27,472, 52,173)	108.10 (107.00, 109.21)	−0.11 (−0.17, −0.04)	−23.93 (−28.28, −19.13)
High-income North America	98,314 (68,399, 134,704)	131.39 (130.56, 132.22)	102,433 (71,882, 139,154)	123.42 (122.67, 124.18)	−0.40 (−0.68, −0.12)	4.19 (1.74, 6.98)
Western Europe	42,780 (30,230, 58,258)	44.55 (44.12, 44.97)	38,736 (27,281, 52,990)	43.55 (43.11, 43.99)	0.03 (−0.06, 0.11)	−9.45 (−12.77, −5.37)
Australasia	4,866 (3,376, 6,556)	90.34 (87.82, 92.92)	6,039 (4,199, 8,127)	85.17 (83.02, 87.37)	−0.22 (−0.28, −0.17)	24.10 (15.18, 33.26)
Andean Latin America	37,870 (25,943, 51,116)	362.18 (358.44, 365.95)	63,119 (43,523, 85,333)	353.65 (350.89, 356.42)	−1.62 (−2.03, −1.20)	66.67 (55.75, 80.59)
Tropical Latin America	143,785 (96,204, 196,060)	342.84 (341.04, 344.65)	303,423 (219,553, 387,109)	508.03 (506.22, 509.85)	−0.45 (−1.80, 0.92)	111.03 (85.14, 144.49)
Central Latin America	74,931 (51,497, 101,583)	167.88 (166.64, 169.12)	102,849 (72,296, 138,554)	149.90 (148.98, 150.82)	−0.04 (−0.26, 0.19)	37.26 (28.24, 46.96)
Southern Latin America	32,761 (23,856, 43,929)	257.54 (254.75, 260.35)	47,865 (33,558, 64,842)	276.96 (274.48, 279.46)	0.75 (0.50, 1.01)	46.10 (34.40, 60.16)
Caribbean	33,370 (23,856, 44,158)	331.46 (327.85, 335.10)	43,792 (30,863, 58,625)	361.82 (358.44, 365.23)	0.53 (0.37, 0.69)	31.23 (19.06, 42.15)
Central Europe	13,900 (9,844, 18,721)	45.73 (44.97, 46.50)	10,572 (7,544, 14,234)	42.92 (42.09, 43.77)	−0.09 (−0.17, −0.02)	−23.94 (−28.78, −18.56)
Eastern Europe	24,503 (16,818, 34,102)	43.88 (43.33, 44.44)	18,996 (13,191, 25,623)	41.20 (40.59, 41.81)	−0.28 (−0.35, −0.20)	−22.48 (−28.31, −16.69)
Central Asia	8,710 (5,988, 11,737)	48.52 (47.48, 49.59)	10,829 (7,589, 14,562)	44.78 (43.94, 45.64)	−0.64 (−0.76, −0.52)	24.33 (16.36, 33.44)
North Africa and Middle East	117,775 (80,561, 163,456)	141.25 (140.41, 142.08)	212,477 (146,410, 288,694)	132.93 (132.36, 133.49)	−0.13 (−0.27, 0.00)	80.41 (70.32, 94.19)
South Asia	981,798 (660,461, 1,331,976)	365.96 (365.22, 366.69)	1,431,200 (983,195, 1,949,503)	281.41 (280.94, 281.87)	−2.46 (−3.07, −1.84)	45.77 (40.80, 50.92)
Southeast Asia	294,044 (198,556, 404,658)	226.19 (225.36, 227.03)	417,387 (288,110, 572,393)	228.44 (227.75, 229.14)	0.84 (0.60, 1.08)	41.95 (34.34, 51.51)
East Asia	334,520 (228,289, 458,677)	98.28 (97.94, 98.62)	350,512 (245,454, 473,097)	108.98 (108.61, 109.36)	−0.60 (−1.08, −0.13)	4.78 (−8.01, 16.30)
Oceania	12,327 (8,594, 16,549)	727.62 (714.45, 741.00)	23,763 (16,506, 32,288)	656.99 (648.60, 665.46)	−0.75 (−1.00, −0.50)	92.76 (73.52, 112.92)
Western Sub-Saharan Africa	301,399 (210,254, 402,265)	631.41 (629.05, 633.78)	605,566 (416,603, 821,775)	469.84 (468.62, 471.07)	−0.48 (−0.63, −0.32)	100.92 (89.84, 110.48)
Eastern Sub-Saharan Africa	573,680 (425,020, 731,790)	1183.37 (1180.16, 1186.58)	918,296 (648,635, 1,235,092)	789.62 (787.95, 791.29)	−1.75 (−2.00, −1.50)	60.07 (42.19, 77.52)
Central Sub-Saharan Africa	175,594 (127,006, 233,657)	1318.37 (1311.96, 1324.80)	396,285 (275,396, 536,101)	1128.84 (1125.23, 1132.47)	−1.43 (−1.68, −1.18)	125.68 (103.24, 145.14)
Southern Sub-Saharan Africa	286,477 (202,190, 374,509)	1961.74 (1954.33, 1969.19)	212,347 (144,525, 290,354)	957.91 (953.83, 962.00)	−2.30 (−3.24, −1.35)	−25.88 (−35.29, −17.30)
*Prevalence*						
Global	14,038,355 (10,599,589, 18,399,106)	1024.78 (1024.24, 1025.32)	20,475,289 (15,154,286, 27,783,875)	1051.66 (1051.20, 1052.11)	−0.50 (−0.69, −0.31)	45.85 (40.89, 51.02)
Sociodemographic index						
Low SDI	3,769,532 (2,977,121, 4,695,511)	3285.55 (3282.13, 3288.97)	6,300,025 (4,603,829, 8,399,217)	2263.54 (2261.72, 2265.36)	−1.78 (−1.96, −1.60)	67.13 (51.88, 79.87)
Low-middle SDI	4,361,104 (3,236,618, 5,802,665)	1577.57 (1576.06, 1579.09)	6,645,643 (4,894,267, 9,049,357)	1295.86 (1294.87, 1296.85)	−1.38 (−1.62, −1.14)	52.38 (47.76, 57.29)
Middle SDI	4,124,109 (3,062,904, 5,415,506)	908.86 (907.96, 909.76)	5,438,130 (4,019,728, 7,281,702)	883.92 (883.17, 884.66)	−0.60 (−0.82, −0.38)	31.86 (25.98, 37.79)
High-middle SDI	999,491 (717,057, 1,338,143)	354.38 (353.68, 355.08)	1,278,164 (923,093, 1,694,878)	425.14 (424.39, 425.90)	0.22 (0.02, 0.43)	27.88 (20.26, 35.80)
High SDI	771,673 (552,312, 1,045,725)	336.47 (335.72, 337.23)	795,641 (569,528, 1,069,823)	327.86 (327.13, 328.59)	−0.30 (−0.48, −0.12)	3.11 (0.08, 6.29)
GBD regions						
High-income Asia Pacific	180,344 (130,132, 240,948)	400.90 (399.04, 402.76)	137,769 (99,122, 183,088)	370.35 (368.34, 372.36)	−0.21 (−0.26, −0.16)	−23.61 (−28.01, −18.77)
High-income North America	355,465 (252,858, 489,844)	467.65 (466.11, 469.20)	366,342 (264,666, 500,019)	436.17 (434.76, 437.59)	−0.42 (−0.70, −0.14)	3.06 (0.40, 5.92)
Western Europe	145,139 (104,191, 192,486)	150.75 (149.97, 151.53)	136,047 (96,941, 178,663)	149.67 (148.87, 150.48)	0.05 (−0.01, 0.11)	−6.26 (−9.95, −1.97)
Australasia	16,062 (11,374, 21,328)	297.34 (292.75, 301.98)	20,292 (14,369, 27,374)	279.70 (275.84, 283.60)	−0.23 (−0.26, −0.20)	26.34 (15.43, 37.65)
Andean Latin America	138,167 (95,757, 188,782)	1391.67 (1384.14, 1399.23)	225,384 (159,125, 313,419)	1264.06 (1258.83, 1269.30)	−2.10 (−2.58, −1.63)	63.12 (51.78, 75.60)
Tropical Latin America	541,365 (385,993, 725,418)	1336.00 (1332.38, 1339.63)	1,192,268 (959,706, 1,495,887)	1970.43 (1966.88, 1973.98)	−0.41 (−1.74, 0.94)	120.23 (89.19, 161.64)
Central Latin America	269,034 (192,450, 364,365)	635.64 (633.16, 638.12)	388,527 (283,911, 526,632)	568.93 (567.15, 570.73)	0.28 (0.04, 0.52)	44.42 (35.74, 53.97)
Southern Latin America	122,603 (90,747, 158,408)	983.43 (977.91, 988.97)	182,700 (133,785, 246,465)	1046.64 (1041.84, 1051.46)	0.81 (0.48, 1.15)	49.02 (35.34, 62.96)
Caribbean	133,400 (99,356, 172,654)	1386.60 (1379.04, 1394.20)	179,861 (132,301, 237,069)	1487.48 (1480.61, 1494.37)	0.65 (0.47, 0.84)	34.83 (22.33, 46.77)
Central Europe	50,475 (36,113, 67,225)	164.78 (163.34, 166.23)	38,902 (28,341, 50,501)	152.57 (151.01, 154.14)	−0.11 (−0.19, −0.03)	−22.93 (−27.82, −17.23)
Eastern Europe	92,013 (65,173, 126,015)	162.13 (161.08, 163.19)	72,790 (52,476, 97,878)	150.58 (149.45, 151.72)	−0.34 (−0.43, −0.25)	−20.89 (−25.50, −15.39)
Central Asia	31,550 (22,268, 42,049)	181.31 (179.24, 183.40)	45,036 (32,490, 60,165)	182.60 (180.91, 184.30)	0.12 (−0.07, 0.31)	42.75 (30.88, 64.78)
North Africa and Middle East	474,359 (332,028, 643,173)	609.02 (607.23, 610.81)	885,582 (618,006, 1,173,990)	555.01 (553.85, 556.17)	0.11 (−0.24, 0.46)	86.69 (74.64, 100.78)
South Asia	3,623,206 (2,630,329, 4,873,818)	1410.73 (1409.25, 1412.21)	5,254,614 (3,794,409, 7,207,149)	1050.08 (1049.18, 1050.98)	−2.76 (−3.43, −2.09)	45.03 (39.69, 50.68)
Southeast Asia	1,100,324 (771,261, 1,497,643)	885.93 (884.23, 887.62)	1,628,559 (1,179,348, 2,156,672)	889.86 (888.49, 891.23)	0.89 (0.61, 1.16)	48.01 (40.20, 57.29)
East Asia	1,274,356 (913,622, 1,721,842)	385.12 (384.44, 385.81)	1,399,766 (988,230, 1,897,262)	420.43 (419.72, 421.15)	−0.67 (−1.14, −0.19)	9.84 (−0.41, 19.67)
Oceania	53,187 (39,583, 70,659)	3384.51 (3354.93, 3414.31)	105,537 (75,966, 141,685)	3015.19 (2996.89, 3033.58)	−0.95 (−1.27, −0.63)	98.43 (78.21, 118.46)
Western Sub-Saharan Africa	1,240,200 (928,220, 1,616,952)	2793.81 (2788.67, 2798.96)	2,370,058 (1,701,680, 3,158,937)	1950.45 (1947.88, 1953.02)	−0.69 (−0.82, −0.56)	91.10 (79.46, 101.10)
Eastern Sub-Saharan Africa	2,384,903 (1,952,007, 2,889,492)	5314.33 (5307.28, 5321.40)	3,486,184 (2,558,678, 4,595,677)	3193.00 (3189.53, 3196.47)	−2.19 (−2.44, −1.95)	46.18 (28.37, 62.28)
Central Sub-Saharan Africa	706,358 (536,153, 925,398)	5663.51 (5649.81, 5677.24)	1,477,853 (1,074,382, 1,985,235)	4488.92 (4481.49, 4496.37)	−2.06 (−2.40, −1.73)	109.22 (89.65, 127.81)
Southern Sub-Saharan Africa	1,105,847 (880,626, 1,357,959)	8083.14 (8067.58, 8098.73)	881,218 (642,500, 1,182,779)	3989.00 (3980.65, 3997.36)	−2.36 (−3.40, −1.31)	−20.31 (−31.84, −9.04)
*DALYs*						
Global	34,108 (26,095, 43,215)	2.55 (2.52, 2.58)	39,592 (30,064, 53,302)	2.03 (2.01, 2.05)	−1.30 (−1.53, −1.06)	16.08 (3.48, 32.74)
Sociodemographic index						
Low SDI	11,911 (8,643, 15,610)	10.48 (10.29, 10.68)	15,298 (11,548, 21,448)	5.48 (5.39, 5.57)	−2.62 (−2.81, −2.43)	28.44 (10.25, 59.73)
Low-middle SDI	13,003 (9,921, 16,480)	4.90 (4.82, 4.99)	14,798 (10,894, 19,707)	2.95 (2.90, 3.00)	−2.29 (−2.59, −2.00)	13.80 (−1.28, 33.83)
Middle SDI	7,069 (5,408, 9,199)	1.64 (1.60, 1.68)	7,586 (5,711, 10,241)	1.22 (1.19, 1.25)	−1.35 (−1.60, −1.09)	7.31 (−3.94, 22.14)
High-middle SDI	1,448 (1,179, 1,876)	0.54 (0.51, 0.57)	1,325 (1,034, 1,855)	0.41 (0.39, 0.44)	−1.38 (−1.70, −1.07)	−8.53 (−17.95, 1.66)
High SDI	653 (493, 949)	0.29 (0.26, 0.31)	549 (385, 866)	0.22 (0.20, 0.23)	−0.99 (−1.07, −0.91)	−16.00 (−22.46, −8.59)
GBD regions						
High-income Asia Pacific	88 (52, 155)	0.19 (0.15, 0.24)	74 (45, 127)	0.18 (0.14, 0.23)	−0.19 (−0.33, −0.06)	−15.92 (−25.43, −5.40)
High-income North America	306 (230,4 30)	0.41 (0.36, 0.45)	271 (195, 402)	0.31 (0.28, 0.35)	−0.89 (−1.00, −0.78)	−11.55 (−17.22, −5.33)
Western Europe	159 (129, 220)	0.17 (0.14, 0.19)	106 (77, 164)	0.11 (0.09, 0.13)	−1.20 (−1.33, −1.07)	−33.26 (−41.41, −24.82)
Australasia	12 (9, 18)	0.23 (0.12, 0.40)	9 (5, 17)	0.12 (0.06, 0.24)	−1.66 (−2.02, −1.30)	−26.57 (−48.51, 1.82)
Andean Latin America	188 (138, 257)	1.96 (1.68, 2.28)	160 (111, 250)	0.91 (0.77, 1.06)	−3.41 (−3.71, −3.11)	−14.59 (−34.27, 10.22)
Tropical Latin America	759 (622, 994)	1.94 (1.80, 2.09)	1,215 (935, 1,659)	1.99 (1.88, 2.10)	−0.63 (−1.20, −0.06)	60.03 (45.75, 75.49)
Central Latin America	566 (496, 683)	1.42 (1.30, 1.55)	906 (746, 1,162)	1.33 (1.25, 1.42)	0.54 (0.03, 1.05)	60.27 (37.88, 92.83)
Southern Latin America	77 (53, 122)	0.62 (0.49, 0.78)	111 (75, 182)	0.64 (0.52, 0.77)	0.42 (0.18, 0.66)	44.71 (22.38, 68.87)
Caribbean	298 (232, 385)	3.25 (2.88, 3.65)	492 (362, 649)	4.09 (3.74, 4.47)	0.96 (0.81, 1.11)	65.10 (26.82, 116.11)
Central Europe	90 (78, 109)	0.29 (0.23, 0.36)	44 (34, 61)	0.16 (0.11, 0.21)	−1.87 (−2.02, −1.71)	−50.89 (−59.41, −39.71)
Eastern Europe	436 (408, 487)	0.79 (0.71, 0.86)	370 (317, 439)	0.68 (0.61, 0.76)	−1.26 (−1.92, −0.60)	−15.16 (−25.92, −4.60)
Central Asia	180 (156, 206)	1.10 (0.94, 1.28)	111 (85, 158)	0.46 (0.38, 0.55)	−3.46 (−4.14, −2.78)	−38.29 (−51.49, −18.15)
North Africa and Middle East	463 (331, 670)	0.60 (0.54, 0.66)	564 (367, 907)	0.35 (0.32, 0.38)	−1.16 (−1.48, −0.83)	21.74 (2.08, 43.53)
South Asia	14,167 (10,504, 18,268)	5.74 (5.64, 5.84)	14,457 (10,478, 19,153)	2.97 (2.92, 3.02)	−3.05 (−3.49, −2.61)	2.04 (−13.69, 21.40)
Southeast Asia	1,650 (1,103, 2,408)	1.38 (1.31, 1.45)	2,821 (1,886, 4,019)	1.54 (1.48, 1.60)	0.63 (0.46, 0.80)	70.96 (48.53, 94.83)
East Asia	2,061 (1,406, 2,899)	0.67 (0.64, 0.70)	959 (645, 1,498)	0.27 (0.25, 0.29)	−3.39 (−3.59, −3.20)	−53.46 (−65.68, −35.65)
Oceania	62 (39, 92)	4.11 (3.11, 5.36)	142 (92, 211)	4.13 (3.47, 4.88)	−0.37 (−0.63, −0.11)	129.55 (67.75, 222.09)
Western Sub-Saharan Africa	2,155 (1,533, 2,879)	4.88 (4.66, 5.10)	4,087 (2,889, 5,596)	3.36 (3.25, 3.47)	−1.14 (−1.22, −1.05)	89.62 (70.35, 112.78)
Eastern Sub-Saharan Africa	7,502 (5,281, 9,982)	16.65 (16.26, 17.06)	9,096 (6,546, 13,687)	8.08 (7.91, 8.26)	−2.82 (−3.05, −2.60)	21.25 (−3.07, 66.06)
Central Sub-Saharan Africa	1,593 (1,128, 2,162)	13.02 (12.36, 13.71)	2,348 (1,673, 3,426)	7.25 (6.95, 7.56)	−2.71 (−3.02, −2.39)	47.46 (14.51, 86.40)
Southern Sub-Saharan Africa	1,297 (948, 1,796)	9.97 (9.41, 10.55)	1,248 (911, 1,753)	5.75 (5.43, 6.08)	−2.12 (−2.56, −1.67)	−3.76 (−23.09, 40.75)

WCBA, women of childbearing age; DALYs, disability-adjusted life years; ASR, age-standardized rate; CI, confidence interval; EAPC, estimated annual percentage change; UI, uncertainty interval; SDI, sociodemographic index; GBD, Global Burden of Disease.

### 3.2 SDI regional level

**Figure 2:**
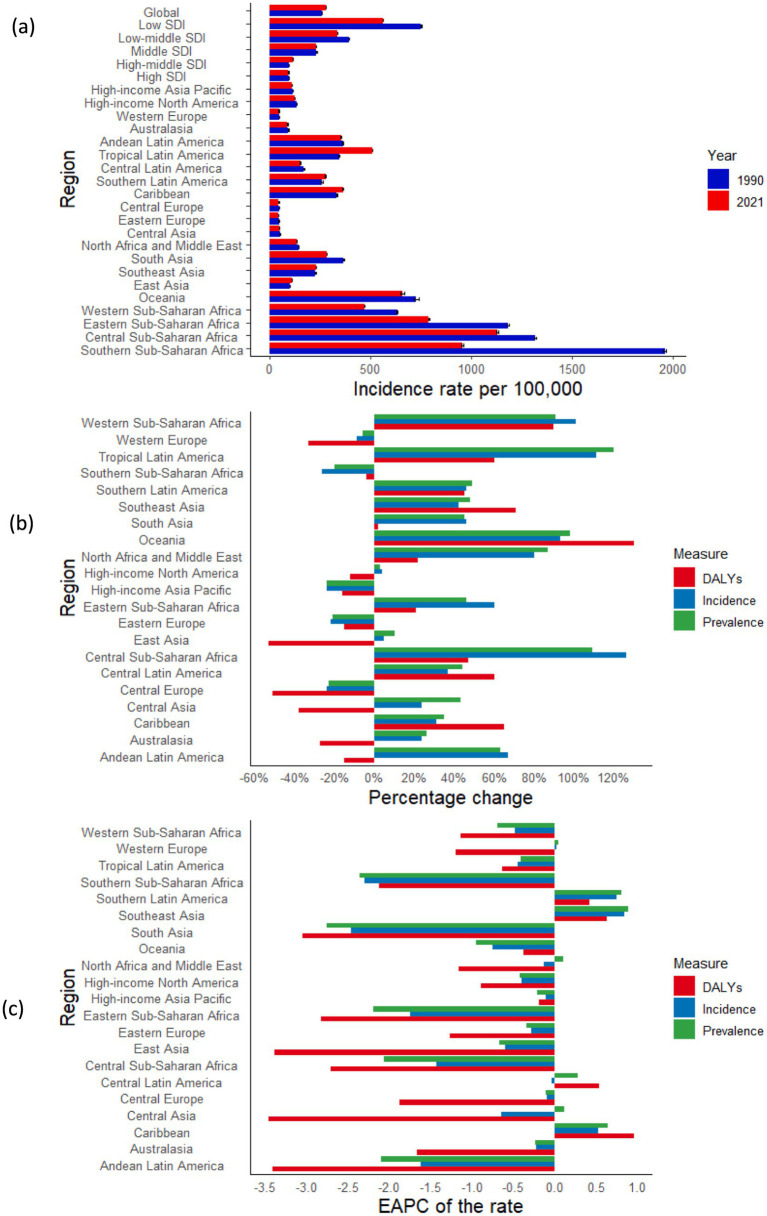
Temporal trend of syphilis burden in WCBA across 21 GBD regions: **(a)** Incidence rate per 100,000 population in 1990 and 2021; **(b)** Percentage change in cases of prevalent, incident, and DALYs in 1990 and 2021; **(c)** EAPC of rates of prevalent, incident, and DALYs from 1990 to 2021. WCBA, women of childbearing age; EAPC, estimated annual percentage change; DALYs, disability-adjusted life years; GBD, Global Burden of Disease.

In 2021, within the WCBA across various SDI regions, the low-middle SDI category registered the highest number of incident syphilis cases, amounting to 1.75 million (95% uncertainty interval [UI]: 1.20 to 2.37), and prevalent cases at 6.65 million (95% UI: 9.01 to 12.59). Conversely, the highest burden in terms of DALYs was noted in the low SDI region, with a total of 15.30 thousand (95% UI: 11.55 to 21.45), constituting 32.67, 32.46, and 38.64% of the global totals for incident cases, prevalent cases, and DALYs, respectively. The highest rates of prevalence, incidence, and DALYs were found in the low SDI regions (Table 1; Figures 1c, 2a; Supplementary Figure S3). As the SDI decreased, the cases of prevalence, incidence, and DALYs exhibited a gradual increase, with the low SDI region demonstrating the most significant percentage change, ranging from approximately 30 to 80%, while the high SDI regions showed the minimal percentage change (Figure 1a). Additionally, from 1990 to 2021, both incidence and prevalence rates in high-middle SDI regions displayed upward trends, with EAPCs of 0.28 (95% CI: 0.08 to 0.48) and 0.22 (95% CI: 0.02 to 0.43), respectively (Table 1; Figure 1b).

### 3.3 GBD regional level

The absolute numbers of prevalence, incidence, and DALYs associated with syphilis in WCBA have increased over time in 11 regions, including Tropical Latin America, South Asia, and Western Sub-Saharan Africa. In contrast, regions such as High-Income Asia Pacific, Western Europe, Central Europe, and Eastern Europe—classified as high SDI or high-middle SDI regions—have exhibited a decrease in these metrics. Over the past 32 years, consistent declines in incidence, prevalence, and DALYs related to syphilis among WCBA have been observed in the majority of regions, with South Asia experiencing the largest decreases in both incidence and prevalence, reflected by EAPCs of −2.46 (95% CI: −3.07 to −1.84) and −2.76 (95% CI: −3.43 to −2.09), respectively. However, Central Asia reported the greatest burden in terms of DALYs, with an EAPC of −3.46 (95% CI: −4.14 to −2.78). In contrast, regions such as Southern Latin America, Southeast Asia, and the Caribbean, classified as middle SDI or high-middle SDI regions, have shown an increase in these metrics. Significantly, the High-Income Asia Pacific region demonstrated a marked decline in both the absolute figures and rates of prevalence, incidence, and DALYs related to syphilis, suggesting a reduced burden of the disease in this area. This trend is likely associated with advancements in healthcare and enhancements in economic conditions (Table 1; Figure 2; Supplementary Figure S3).

### 3.4 Countries level

**Figure 3:**
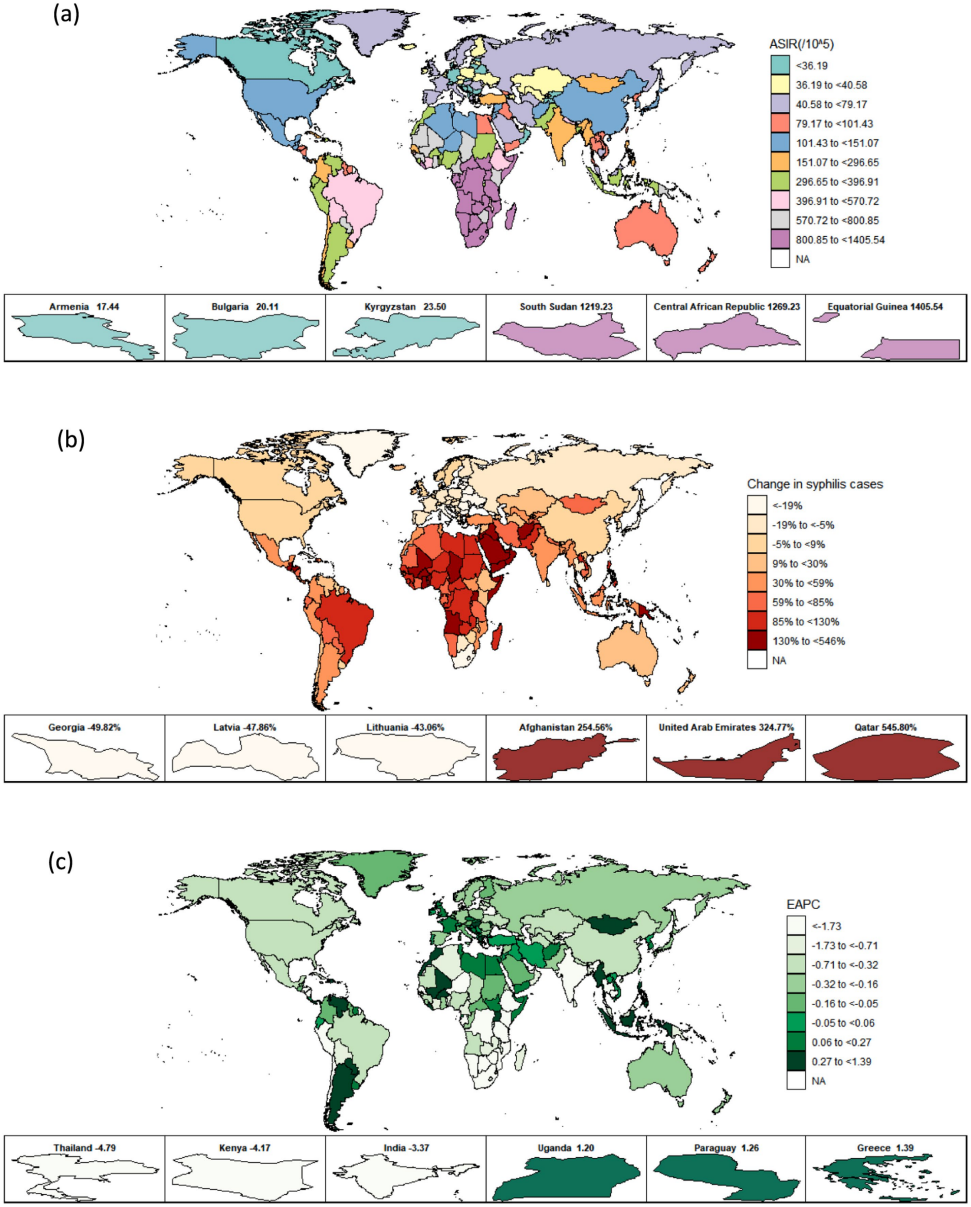
Temporal trend of syphilis incidence in WCBA across 204 countries: **(a)** Age-standardized incidence rate (ASIR) in 2021; **(b)** Percentage change in incident cases in 1990 and 2021; **(c)** EAPC in incident rates from 1990 to 2021. WCBA, women of childbearing age; EAPC, estimated annual percentage change.

Approximately 60% of countries showed an increasing trend in the cases of prevalence, incidence, and DALYs associated with syphilis among WCBA from 1990 to 2021. Qatar, classified as a high SDI region, experienced the highest percentage changes in both prevalence and incidence, exceeding 500%. Meanwhile, Kuwait, also categorized as a high SDI region, reported the greatest burden in terms of DALYs, with a percentage change of 664.05% (95% UI: 348.03 to 1570.57). Additionally, only 40% of countries exhibited a decrease in the prevalence, incidence, and DALYs associated with syphilis, including Georgia, Lithuania, and Latvia, all of which are classified as high SDI regions, with percentage changes ranging from 42 to 74%. This indicates a diverging trend in the rise of syphilis cases among WCBA in countries classified as high SDI regions. Over the past 32 years, the majority of countries have demonstrated downward trends in both prevalence and incidence rates, with the most significant decrease observed in Thailand, classified in the middle SDI region, showing an EAPC of −4.79 (95% CI: −6.17 to −3.39) for prevalence rates and −5.55 (95% CI: −7.08 to −3.99) for incidence rates, in line with the global trend in middle SDI regions. In contrast, approximately one-quarter of the countries exhibited an upward trend in DALYs rates, particularly in Dominica, where the EAPC was 4.00 (95% CI: 3.53 to 4.48). Despite this, the majority of countries showed a decreasing trend in DALYs rates, with Armenia experiencing the most significant decline at an EAPC of −5.93 (95% CI: −6.82 to −5.04). Additionally, in 2021, the highest rates of incidence, prevalence, and DALYs were reported in Equatorial Guinea and Central African Republic (Supplementary Tables S1–S3, Figure 3; Supplementary Figures S4, S5).

Primarily, the hierarchical clustering analysis of the EAPC in ASR pertaining to prevalence, incidence, and DALYs revealed that the 204 countries were categorized into three distinct clusters: 41 countries were classified as exhibiting a ‘significant decrease’ corresponding to low and low-middle SDI; 58 countries were identified as experiencing a ‘decrease’ akin to the global average and high SDI; and 105 countries were recognized as showing an ‘increase’, including nations such as the Central African Republic, Somalia, Liberia, and South Sudan (Supplementary Figure S2).

### 3.5 Age patterns

**Figure 4:**
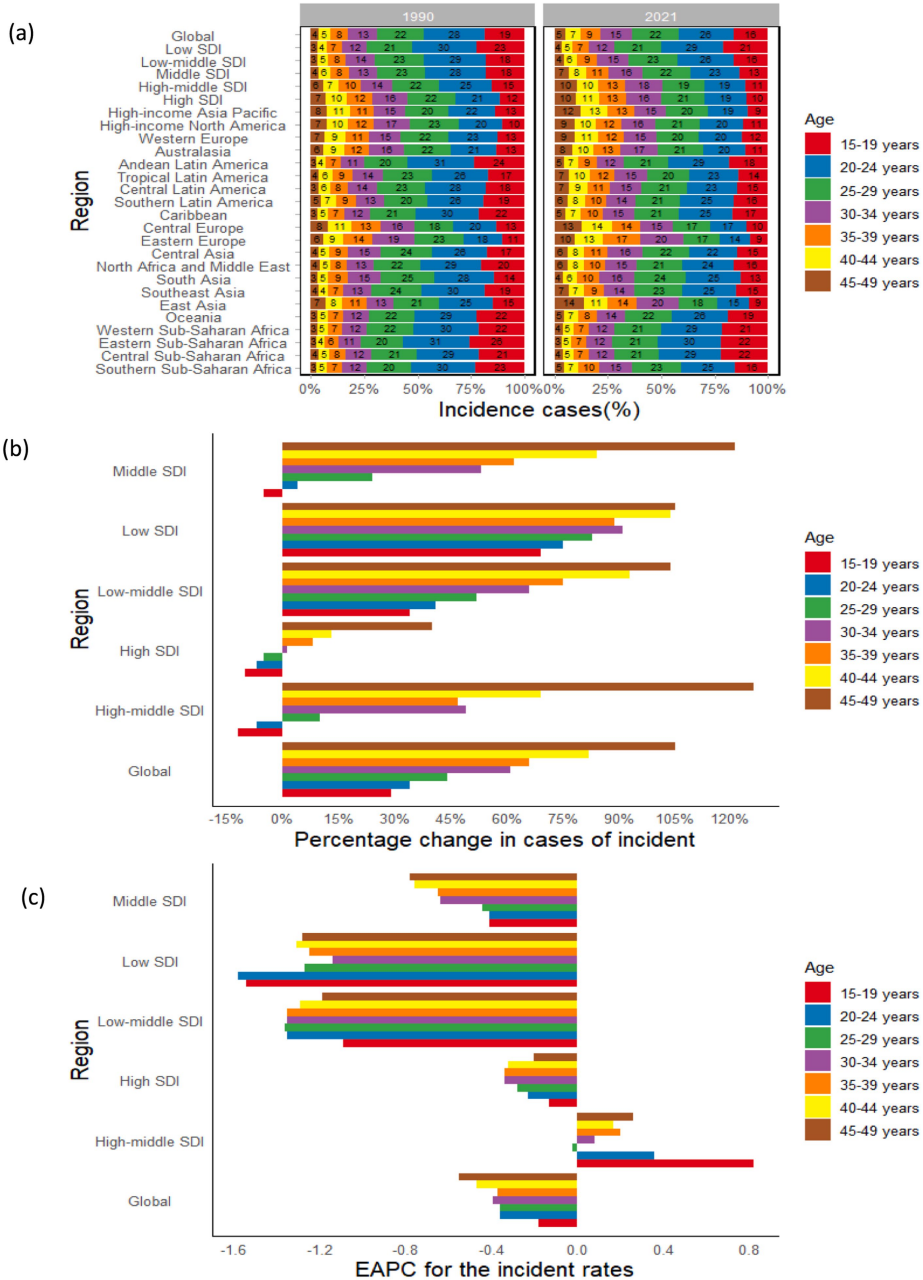
Temporal trend of syphilis burden in WCBA by age pattern across SDI and GBD regions: **(a)** The distribution of incident cases across 7 age groups as percentages in 1990 and 2021; **(b)** Percentage change in incident cases of 7 age groups in 1990 and 2021 across SDI regions; **(c)** EAPC of incident rates of 7 age groups from 1990 to 2021 across SDI regions. WCBA, women of childbearing age; EAPC, estimated annual percentage change; SDI, sociodemographic index; GBD, Global Burden of Disease.

The detailed trends of syphilis cases over the past 32 years in both the global and the five SDI regions were illustrated in Table 2, Figure 4, and Supplementary Figure S6. The percentage change in the prevalence, incidence, and DALYs of syphilis among WCBA globally exhibited an upward trend with advancing age, with the most minimal changes observed in the 15–29 age cohorts: specifically, the 15–19 age group demonstrated a 29.13% increase in incidence, while the 20–24 age group reflected a 29.52% rise in prevalence, and the 25–29 age group indicates a 7.74% increase in DALYs. In contrast, the 45–49 age group exhibited the highest percentage changes, with increases of 104.68% in incidence, 93.06% in prevalence, and 38.80% in DALYs, which were approximately 3–4 times greater than the increments seen in the 15–29 age groups.

**Table 2:** Number of cases, incidence, prevalence, and DALYs rates of syphilis among WCBA in 1990 and 2021; percentage change in prevalent, incident, and DALYs cases between 1990 and 2021; and EAPC of these rates from 1990 to 2021, by age groups across SDI regions.

Items	1990		2021		1990–2021	
	Cases number	Rates per 100,000	Cases number	Rates per 100,000	EAPC	Cases change (%)
	No. (95% UI)	No. (95% UI)	No. (95% UI)	No. (95% UI)	No. (95% CI)	No. (95% UI)
*Incidence*						
Global						
15–19 years	679,698 (389,380, 1,087,604)	265.99 (152.38, 425.61)	877,717 (455,417, 1,433,595)	289.06 (149.98, 472.12)	−0.18 (−0.34, −0.02)	29.13 (15.03, 36.36)
20–24 years	1,025,646 (581,840, 1,625,953)	420.11 (238.33, 666.01)	1,372,862 (757,971, 2,213,692)	467.35 (258.03, 753.59)	−0.36 (−0.62, −0.11)	33.85 (26.38, 39.30)
25–29 years	813,226 (442,398, 1,346,339)	369.48 (201.00, 611.70)	1,173,637 (627,079, 1,940,795)	403.33 (215.50, 666.97)	−0.36 (−0.63, −0.10)	44.32 (38.67, 49.12)
30–34 years	484,046 (248,129, 769,981)	254.61 (130.52, 405.02)	781,030 (404,365, 1,253,135)	261.27 (135.27, 419.21)	−0.39 (−0.58, −0.20)	61.35 (56.13, 66.73)
35–39 years	303,173 (156,659, 504,016)	174.79 (90.32, 290.58)	502,822 (260,234, 841,339)	181.00 (93.68, 302.85)	−0.37 (−0.57, −0.18)	65.85 (60.67, 70.31)
40–44 years	199,558 (98,366, 341,342)	142.31 (70.15, 243.42)	362,319 (178,878, 623,621)	146.04 (72.10, 251.37)	−0.47 (−0.69, −0.25)	81.56 (76.52, 86.90)
45–49 years	139,265 (70,267, 252,171)	122.38 (61.75, 221.59)	285,052 (144,839, 514,957)	120.97 (61.47, 218.53)	−0.55 (−0.72, −0.39)	104.68 (98.51, 111.73)
15–49 years	3,644,611 (2,528,551, 4,866,997)	272.52 (189.07, 363.93)	5,355,440 (3,717,326, 7,240,281)	274.80 (190.74, 371.51)	−0.52 (−0.72, −0.33)	46.94 (42.49, 50.86)
Low SDI						
15–19 years	207,146 (130,378, 319,047)	824.34 (518.84, 1269.64)	351,085 (184,112, 576,346)	569.40 (298.60, 934.74)	−1.54 (−1.70, −1.39)	69.49 (35.09, 92.32)
20–24 years	274,757 (169,855, 419,957)	1264.95 (781.99, 1933.43)	482,018 (267,697, 774,529)	914.08 (507.65, 1468.79)	−1.58 (−1.79, −1.37)	75.43 (51.94, 94.74)
25–29 years	193,951 (107,179, 314,128)	1047.06 (578.61, 1695.85)	355,318 (193,674, 586,877)	806.64 (439.68, 1332.33)	−1.27 (−1.51, −1.03)	83.20 (64.45, 99.92)
30–34 years	107,937 (55,533, 172,247)	709.60 (365.09, 1132.39)	205,831 (110,216, 334,588)	554.52 (296.93, 901.41)	−1.14 (−1.37, −0.90)	90.70 (75.76, 106.68)
35–39 years	64,160 (33,144, 104,105)	497.96 (257.24, 807.98)	121,013 (61,483, 203,037)	379.76 (192.95, 637.16)	−1.25 (−1.53, −0.97)	88.61 (75.28, 102.63)
40–44 years	40,676 (20,380, 68,006)	410.24 (205.55, 685.88)	82,813 (39,318, 143,195)	317.05 (150.53, 548.22)	−1.31 (−1.57,− 1.05)	103.59 (87.23, 118.96)
45–49 years	28,468 (14,970, 49,295)	342.92 (180.32, 593.80)	58,276 (28,797, 105,378)	280.61 (138.66, 507.42)	−1.28 (−1.47, −1.09)	104.71 (89.45, 120.88)
15–49 years	917,095 (658,897, 1,199,367)	821.14 (589.96, 1073.88)	1,656,353 (1,160,830, 2,227,205)	603.82 (423.18, 811.93)	−1.43 (−1.61, −1.25)	80.61 (64.41, 93.17)
Low-middle SDI						
15–19 years	207,813 (111,543, 344,043)	353.71 (189.85, 585.59)	278,949 (141,975, 466,385)	308.57 (157.05, 515.91)	−1.09 (−1.30, −0.88)	34.23 (24.91, 40.02)
20–24 years	328,736 (184,612, 523,300)	630.31 (353.97, 1003.37)	461,967 (253,176, 747,885)	530.94 (290.98, 859.55)	−1.35 (−1.61, −1.08)	40.53 (34.00, 45.97)
25–29 years	264,100 (143,106, 434,891)	588.42 (318.84, 968.95)	401,301 (215,020, 663,403)	493.93 (264.65, 816.53)	−1.36 (−1.67, −1.06)	51.95 (46.09, 57.23)
30–34 years	156,022 (80,011, 251,731)	416.68 (213.68, 672.29)	258,371 (133,753,4 16,050)	349.58 (180.97, 562.93)	−1.35 (−1.65, −1.05)	65.60 (59.82, 72.16)
35–39 years	92,953 (46,900, 154,637)	290.17 (146.41, 482.73)	163,087 (83,990, 270,170)	244.92 (126.14, 405.74)	−1.35 (−1.65, −1.05)	75.45 (68.37, 82.32)
40–44 years	56,624 (27,360, 98,482)	218.25 (105.46, 379.59)	109,555 (54,010, 187,500)	189.93 (93.64, 325.06)	−1.29 (−1.60, −0.98)	93.48 (87.12, 101.88)
45–49 years	37,261 (18,417, 67,326)	171.67 (84.85, 310.18)	76,147 (37,983, 136,985)	154.03 (76.83, 277.09)	−1.19 (−1.47, −0.91)	104.36 (96.23, 114.07)
15–49 years	1,143,508 (784,737, 1,531,944)	419.00 (287.54, 561.32)	1,749,377 (1,201,233, 2,371,568)	345.54 (237.27, 468.44)	−1.39 (−1.66, −1.12)	52.98 (48.00, 57.46)
Middle SDI						
15–19 years	198,684 (111,567, 318,075)	215.48 (121.00, 344.97)	188,632 (99,291, 311,494)	214.75 (113.04, 354.62)	−0.41 (−0.64, −0.18)	−5.06 (−11.98, −1.38)
20–24 years	310,234 (173,852, 498,374)	351.21 (196.82, 564.20)	324,009 (178,631, 519,508)	373.85 (206.11, 599.43)	−0.41 (−0.69, −0.13)	4.44 (−0.06, 7.48)
25–29 years	248,918 (131,994, 416,437)	332.30 (176.21, 555.93)	307,464 (163,068, 512,221)	339.37 (179.99, 565.37)	−0.44 (−0.68, −0.20)	23.52 (19.42, 27.22)
30–34 years	145,810 (73,504, 232,468)	242.86 (122.43, 387.20)	223,201 (111,459, 356,498)	226.02 (112.87, 361.00)	−0.64 (−0.83, −0.45)	53.08 (48.44, 57.61)
35–39 years	92,668 (47,650, 152,864)	167.18 (85.96, 275.77)	150,012 (77,609, 248,038)	163.68 (84.68, 270.64)	−0.65 (−0.88, −0.42)	61.88 (56.04, 67.07)
40–44 years	61,097 (30,535, 105,283)	144.59 (72.26, 249.15)	112,117 (56,676, 194,352)	136.98 (69.24, 237.45)	−0.76 (−1.01, −0.51)	83.51 (78.31, 89.31)
45–49 years	42,801 (21,643, 78,045)	126.28 (63.86, 230.27)	94,473 (49,132, 170,226)	116.47 (60.57, 209.87)	−0.78 (−0.95, −0.61)	120.72 (114.53, 129.29)
15–49 years	1,100,213 (749,009, 1,492,787)	246.10 (167.54, 333.91)	1,399,908 (979,372, 1,895,219)	226.35 (158.35, 306.44)	−0.78 (−0.99, −0.56)	27.24 (20.45, 33.65)
High-middle SDI						
15–19 years	40,599 (21,592, 66,896)	85.72 (45.59, 141.25)	35,887 (18,884, 58,641)	104.22 (54.84, 170.30)	0.82 (0.64, 1.01)	−11.61 (−14.76, −7.92)
20–24 years	66,469 (36,648, 108,589)	138.07 (76.13, 225.57)	62,106 (33,824, 101,482)	174.48 (95.02, 285.10)	0.36 (0.15, 0.57)	−6.56 (−9.26, −3.27)
25–29 years	58,523 (30,234, 99,818)	127.93 (66.09, 218.20)	64,128 (33,198, 107,458)	159.09 (82.36, 266.58)	−0.02 (−0.29, 0.24)	9.58 (5.69, 13.64)
30–34 years	38,800 (19,459, 63,154)	92.94 (46.61, 151.27)	57,716 (28,865, 94,542)	112.09 (56.06, 183.61)	0.08 (−0.19, 0.35)	48.75 (43.13, 55.40)
35–39 years	28,057 (14,530, 47,923)	71.04 (36.79, 121.33)	41,278 (21,169, 69,994)	83.30 (42.72, 141.26)	0.20 (−0.04, 0.45)	47.12 (42.14, 52.69)
40–44 years	19,922 (9,675, 35,121)	64.80 (31.47, 114.24)	33,754 (16,418, 59,877)	74.15 (36.07, 131.54)	0.17 (0.01, 0.34)	69.43 (64.38, 75.15)
45–49 years	15,238 (7,723, 27,715)	62.15 (31.50, 113.04)	34,373 (17,208, 62,645)	71.27 (35.68, 129.89)	0.26 (0.17, 0.35)	125.58 (116.03, 135.68)
15–49 years	267,608 (185,166, 365,761)	96.35 (66.67, 131.69)	329,244 (231,640,4 41,593)	107.90 (75.92, 144.72)	0.04 (−0.15, 0.24)	23.03 (14.05, 32.20)
High SDI						
15–19 years	24,802 (12,796, 41,089)	77.82 (40.15, 128.93)	22,399 (11,637, 37,198)	76.99 (40.00, 127.86)	−0.13 (−0.33, 0.06)	−9.69 (−12.23, −7.72)
20–24 years	44,560 (24,256, 72,066)	132.70 (72.24, 214.62)	41,641 (22,600, 67,956)	132.09 (71.69,215.57)	−0.23 (−0.45, −0.02)	−6.55 (−8.38, −4.41)
25–29 years	47,078 (24,495, 79,253)	131.33 (68.33, 221.09)	44,504 (23,075, 75,343)	128.78 (66.77, 218.01)	−0.28 (−0.49, −0.08)	−5.47 (−7.49, −3.32)
30–34 years	35,099 (17,585, 57,483)	98.91 (49.56, 161.99)	35,309 (17,703, 57,569)	94.33 (47.29, 153.79)	−0.34 (−0.51, −0.16)	0.60 (−2.17, 3.13)
35–39 years	25,102 (13,000, 43,328)	75.07 (38.88, 129.57)	27,041 (14,038, 46,787)	71.29 (37.01, 123.34)	−0.34 (−0.50, −0.19)	7.72 (4.60, 10.45)
40–44 years	21,076 (10,247, 37,229)	67.50 (32.82, 119.24)	23,781 (11,650, 42,080)	64.78 (31.73, 114.62)	−0.32 (−0.48, −0.17)	12.84 (9.35, 15.87)
45–49 years	15,383 (7,769, 28,171)	60.88 (30.75, 111.50)	21,555 (10,930,3 9,210)	60.03 (30.44, 109.20)	−0.20 (−0.34, −0.06)	40.12 (34.66, 44.96)
15–49 years	213,100 (148,910,290,712)	94.00 (65.68,128.23)	216,230 (151,386, 293,382)	88.93 (62.26, 120.65)	−0.32 (−0.48, −0.16)	1.47 (−1.52, 5.00)
*Prevalence*						
Global						
15–19 years	1,803,191 (1,297,873, 2,453,620)	705.64 (507.90, 960.18)	2,395,865 (1,705,038, 3,362,825)	789.02 (561.51, 1107.47)	−0.10 (−0.29, 0.09)	32.87 (25.16, 38.98)
20–24 years	3,097,219 (1,869,320, 4,598,731)	1268.65 (765.69, 1883.68)	4,011,432 (2,251,554, 6,234,135)	1365.58 (766.48, 2122.24)	−0.43 (−0.67, −0.19)	29.52 (18.10, 36.62)
25–29 years	3,409,497 (2,163,960, 5,216,037)	1549.07 (983.17, 2369.85)	4,686,616 (2,784,882, 7,368,650)	1610.59 (957.05, 2532.29)	−0.57 (−0.80, −0.33)	37.46 (29.00, 43.35)
30–34 years	2,471,384 (1,553,369, 3,700,882)	1299.98 (817.09, 1946.72)	3,790,068 (2,317,485, 5,850,227)	1267.87 (775.26, 1957.05)	−0.63 (−0.79, −0.47)	53.36 (45.38, 59.99)
35–39 years	1,607,340 (1,013,713, 2,418,204)	926.67 (584.43, 1394.15)	2,578,667 (1,583,088, 3,968,690)	928.24 (569.86, 1428.60)	−0.53 (−0.70, −0.36)	60.43 (53.49, 65.77)
40–44 years	981,109 (616,094, 1,511,940)	699.67 (439.36, 1078.22)	1,721,841 (1,068,044, 2,691,026)	694.04 (430.51, 1084.70)	−0.57 (−0.78, −0.37)	75.50 (69.65, 80.36)
45–49 years	668,615 (419,029, 1,038,414)	587.53 (368.21, 912.48)	1,290,799 (798,019, 2,037,202)	547.78 (338.66, 864.53)	−0.72 (−0.88, −0.56)	93.06 (85.81, 99.55)
15–49 years	14,038,355 (10,599,589, 18,399,106)	1049.71 (792.57, 1375.78)	20,475,289 (15,154,286, 27,783,875)	1050.63 (777.60, 1425.65)	−0.59 (−0.77, −0.40)	45.85 (40.89, 51.02)
Low SDI						
15–19 years	540,346 (407,069, 712,898)	2150.30 (1619.93, 2836.97)	926,755 (641,750, 1,310,219)	1503.05 (1040.82, 2124.97)	−1.45 (−1.60, −1.30)	71.51 (54.28, 87.46)
20–24 years	914,828 (611,614, 1,282,835)	4211.76 (2815.80, 5906.03)	1,458,737 (833,152, 2,291,417)	2766.31 (1579.96, 4345.38)	−1.89 (−2.07, −1.71)	59.45 (32.55, 80.84)
25–29 years	924,513 (626,160, 1,318,433)	4991.06 (3380.38, 7117.67)	1,471,718 (878,499, 2,301,667)	3341.09 (1994.37, 5225.24)	−1.95 (−2.16, −1.74)	59.19 (36.50, 76.05)
30–34 years	632,257 (440,365, 889,259)	4156.59 (2895.06, 5846.18)	1,053,290 (657,148, 1,580,534)	2837.65 (1770.41, 4258.09)	−1.89 (−2.09, −1.69)	66.59 (44.99, 84.23)
35–39 years	384,335 (264,208, 544,030)	2982.90 (2050.57, 4222.32)	676,131 (417,928, 1,017,004)	2121.81 (1311.53, 3191.53)	−1.71 (−1.91, −1.51)	75.92 (56.52, 90.69)
40–44 years	221,622 (146,224, 323,060)	2235.18 (1474.75, 3258.24)	429,223 (265,617, 667,124)	1643.28 (1016.91, 2554.08)	−1.58 (−1.83, −1.34)	93.67 (74.10, 109.77)
45–49 years	151,631 (101,903, 223,680)	1826.52 (1227.50, 2694.40)	284,172 (178,384, 444,054)	1368.35 (858.96, 2138.22)	−1.55 (−1.78, −1.31)	87.41 (69.23, 102.55)
15–49 years	3,769,532 (2,977,121, 4,695,511)	3375.13 (2665.63, 4204.23)	6,300,025 (4,603,829, 8,399,217)	2296.67 (1678.32, 3061.93)	−1.79 (−1.97, −1.62)	67.13 (51.88, 79.87)
Low-middle SDI						
15–19 years	557,277 (400,228, 769,278)	948.53 (681.22, 1309.37)	765,147 (546,393, 1,066,731)	846.39 (604.41, 1180.00)	−1.04 (−1.28, −0.81)	37.30 (29.89, 43.22)
20–24 years	960,926 (554,957, 1,472,492)	1842.46 (1064.06, 2823.33)	1,325,507 (745,426, 2,082,566)	1523.41 (856.72, 2393.51)	−1.33 (−1.57, −1.10)	37.94 (29.05, 43.63)
25–29 years	1,075,809 (666,362, 1,666,859)	2396.93 (1484.67, 3713.81)	1,581,741 (941,909, 2,476,014)	1946.83 (1159.32, 3047.52)	−1.44 (−1.70, −1.19)	47.03 (39.79, 53.45)
30–34 years	781,780 (494,036, 1,169,807)	2087.88 (1319.41, 3124.17)	1,238,362 (766,466, 1,907,126)	1675.55 (1037.05, 2580.41)	−1.49 (−1.73, −1.24)	58.40 (51.55, 65.60)
35–39 years	498,128 (306,846, 757,511)	1554.99 (957.88, 2364.70)	831,623 (511,806, 1,277,689)	1248.93 (768.63, 1918.83)	−1.46 (−1.70, −1.23)	66.95 (59.19, 73.71)
40–44 years	296,013 (186,299, 448,581)	1140.95 (718.07, 1729.00)	538,461 (336,676, 826,165)	933.50 (583.68, 1432.28)	−1.40 (−1.64, −1.15)	81.90 (74.00, 88.99)
45–49 years	191,170 (122,056, 292,890)	880.76 (562.34, 1349.40)	364,802 (229,620, 566,831)	737.91 (464.47, 1146.57)	−1.30 (−1.53, −1.07)	90.83 (82.30, 99.25)
15–49 years	4,361,104 (3,236,618, 5,802,665)	1597.96 (1185.94, 2126.17)	6,645,643 (4,894,267, 9,049,357)	1312.67 (966.73, 1787.45)	−1.38 (−1.62, −1.15)	52.38 (47.76, 57.29)
Middle SDI						
15–19 years	526,398 (372,053, 720,135)	570.90 (403.51, 781.02)	545,602 (393,507, 748,723)	621.15 (447.99, 852.39)	−0.26 (−0.53, 0.01)	3.65 (−1.83, 8.97)
20–24 years	907,500 (538,354, 1,383,192)	1027.37 (609.47, 1565.90)	934,608 (530,173, 1,467,938)	1078.38 (611.73, 1693.76)	−0.45 (−0.73, −0.18)	2.99 (−3.25, 6.61)
25–29 years	1,008,683 (623,586, 1,563,762)	1346.56 (832.47, 2087.58)	1,217,958 (726,391, 1,902,591)	1344.35 (801.77, 2100.02)	−0.52 (−0.75, −0.29)	20.75 (16.14, 25.11)
30–34 years	718,870 (442,761, 1,096,068)	1197.34 (737.46, 1825.60)	1,070,077 (644,163, 1,663,890)	1083.59 (652.30, 1684.90)	−0.72 (−0.92, −0.52)	48.86 (43.27, 54.52)
35–39 years	474,651 (290,332, 720,506)	856.28 (523.77, 1299.81)	748,216 (451,431, 1,145,754)	816.40 (492.57, 1250.17)	−0.70 (−0.94, −0.45)	57.63 (51.69, 62.82)
40–44 years	289,754 (181,279, 451,451)	685.71 (429.00, 1068.37)	510,066 (316,290,7 99,225)	623.17 (386.42, 976.44)	−0.84 (−1.09, −0.58)	76.03 (69.51, 81.66)
45–49 years	198,252 (121,444, 313,120)	584.94 (358.32, 923.85)	411,603 (253,632, 650,806)	507.46 (312.70, 802.36)	−0.94 (−1.12, −0.76)	107.62 (100.16, 114.28)
15–49 years	4,124,109 (3,062,904, 5,415,506)	922.49 (685.12,1 211.35)	5,438,130 (4,019,728, 7,281,702)	879.29 (649.95, 1177.38)	−0.70 (−0.93, −0.47)	31.86 (25.98, 37.79)
High-middle SDI						
15–19 years	115,052 (77,994, 165,142)	242.93 (164.68, 348.69)	99,935 (69,846, 142,743)	290.22 (202.84, 414.54)	0.62 (0.46, 0.78)	−13.14 (−17.55, −8.37)
20–24 years	191,558 (105,861, 306,223)	397.91 (219.90, 636.10)	176,682 (97,950, 281,800)	496.36 (275.17, 791.67)	0.51 (0.30, 0.71)	−7.77 (−11.38, −4.22)
25–29 years	226,762 (129,384, 369,429)	495.70 (282.83, 807.57)	248,063 (142,823, 400,126)	615.40 (354.32, 992.65)	0.03 (−0.23, 0.30)	9.39 (5.43, 13.39)
30–34 years	180,945 (106,742, 287,031)	433.42 (255.68, 687.53)	267,454 (155,952, 424,283)	519.42 (302.87, 824.00)	0.00 (−0.28, 0.27)	47.81 (41.78, 55.74)
35–39 years	134,788 (77,675, 213,864)	341.26 (196.66, 541.47)	197,612 (115,121, 310,028)	398.81 (232.33, 625.68)	0.17 (−0.07, 0.40)	46.61 (39.55, 53.34)
40–44 years	86,386 (50,675, 138,740)	280.98 (164.83, 451.27)	145,960 (87,649, 235,930)	320.65 (192.55, 518.31)	0.18 (0.02, 0.34)	68.96 (60.76, 76.58)
45–49 years	63,999 (37,050, 106,619)	261.03 (151.12, 434.87)	142,458 (82,966, 235,151)	295.39 (172.03, 487.58)	0.18 (0.09, 0.27)	122.59 (110.98, 132.26)
15–49 years	999,491 (717,057, 1,338,143)	359.85 (258.16, 481.77)	1,278,164 (923,093, 1,694,878)	418.89 (302.53, 555.46)	0.13 (−0.06, 0.32)	27.88 (20.26, 35.80)
High SDI						
15–19 years	62,460 (40,816, 92,891)	195.99 (128.08, 291.48)	56,386 (36,944, 84,422)	193.82 (126.99, 290.19)	−0.15 (−0.32, 0.02)	−9.73 (−12.93, −6.65)
20–24 years	119,581 (65,426, 198,773)	356.13 (194.85, 591.97)	112,435 (60,769, 187,254)	356.67 (192.77, 594.01)	−0.20 (−0.42, 0.01)	−5.98 (−8.53, −3.55)
25–29 years	170,748 (96,528, 277,729)	476.34 (269.28, 774.78)	163,175 (90,856, 266,448)	472.16 (262.90, 770.99)	−0.27 (−0.47, −0.06)	−4.44 (−6.99, −1.64)
30–34 years	155,377 (90,687, 246,318)	437.87 (255.57, 694.15)	157,646 (92,580, 251,862)	421.14 (247.32, 672.83)	−0.37 (−0.55, −0.20)	1.46 (−1.47, 4.87)
35–39 years	114,066 (66,483, 183,108)	341.12 (198.82, 547.59)	122,827 (71,234, 197,792)	323.81 (187.79, 521.43)	−0.41 (−0.56, −0.27)	7.68 (4.37, 11.14)
40–44 years	86,473 (51,940, 140,329)	276.95 (166.35, 449.44)	96,564 (57,765, 156,001)	263.03 (157.35, 424.94)	−0.39 (−0.54, −0.23)	11.67 (8.21, 15.41)
45–49 years	62,967 (35,986, 106,878)	249.21 (142.42, 423.00)	86,608 (50,272, 145,903)	241.21 (140.01, 406.35)	−0.28 (−0.43, −0.12)	37.54 (32.86, 43.04)
15–49 years	771,673 (552,312, 1,045,725)	340.38 (243.62, 461.27)	795,641 (569,528, 1,069,823)	327.21 (234.22, 439.97)	−0.33 (−0.48, −0.17)	3.11 (0.08, 6.29)
*DALYs*						
Global						
15–19 years	5,689 (4,258, 7,520)	2.23 (1.67, 2.94)	6,143 (4,589, 8,469)	2.02 (1.51, 2.79)	−0.97 (−1.20,− 0.74)	7.99 (−7.18, 28.55)
20–24 years	6,123 (4,552, 8,233)	2.51 (1.86, 3.37)	6,647 (4,873, 9,379)	2.26 (1.66, 3.19)	−1.11 (−1.43, −0.79)	8.56 (−7.55, 30.41)
25–29 years	6,619 (4,664, 8,498)	3.01 (2.12, 3.86)	7,132 (5,214, 9,801)	2.45 (1.79, 3.37)	−1.26 (−1.56, −0.96)	7.74 (−7.71, 31.07)
30–34 years	5,342 (3,899, 6,787)	2.81 (2.05, 3.57)	6,311 (4,650, 8,406)	2.11 (1.56, 2.81)	−1.35 (−1.53, −1.18)	18.15 (1.08,3 8.18)
35–39 years	4,000 (3,089, 5,180)	2.31 (1.78, 2.99)	4,917 (3,690, 6,510)	1.77 (1.33, 2.34)	−1.29 (−1.49, −1.09)	22.92 (8.29, 40.66)
40–44 years	3,634 (2,652, 4,627)	2.59 (1.89, 3.30)	4,693 (3,542,6,034)	1.89 (1.43, 2.43)	−1.51 (−1.77, −1.24)	29.12 (10.11, 48.62)
45–49 years	2,701 (2,076, 3,487)	2.37 (1.82, 3.06)	3,749 (2,947, 4,847)	1.59 (1.25, 2.06)	−1.78 (−2.04, −1.53)	38.80 (24.01, 57.55)
15–49 years	34,108 (26,095, 43,215)	2.55 (1.95, 3.23)	39,592 (30,064, 53,302)	2.03 (1.54, 2.74)	−1.31 (−1.55, −1.08)	16.08 (3.48, 32.74)
Low SDI						
15–19 years	2,496 (1,807, 3,350)	9.93 (7.19, 13.33)	3,271 (2,362, 4,687)	5.31 (3.83, 7.60)	−2.56 (−2.75, −2.37)	31.08 (3.24, 71.93)
20–24 years	2,429 (1 733, 3,269)	11.18 (7.98, 15.05)	3,169 (2,253, 4,605)	6.01 (4.27, 8.73)	−2.51 (−2.69, −2.32)	30.47 (2.92, 74.77)
25–29 years	2,427 (1,613, 3,158)	13.10 (8.71, 17.05)	2,943 (2,037, 4,258)	6.68 (4.62, 9.67)	−2.63 (−2.81, −2.44)	21.23 (−3.54, 66.93)
30–34 years	1,735 (1,221, 2,325)	11.41 (8.02, 15.28)	2,226 (1,584, 3,114)	6.00 (4.27, 8.39)	−2.57 (−2.75, −2.39)	28.33 (1.14, 67.78)
35–39 years	1,197 (879, 1,589)	9.29 (6.82, 12.33)	1,524 (1,109, 2,143)	4.78 (3.48, 6.73)	−2.70 (−2.91, −2.49)	27.34 (2.80, 59.46)
40–44 years	891 (645, 1,204)	8.99 (6.51, 12.15)	1,212 (883, 1,691)	4.64 (3.38, 6.48)	−2.77 (−3.01, −2.52)	36.01 (6.31, 69.34)
45–49 years	736 (543, 1,008)	8.86 (6.54, 12.14)	953 (690, 1,357)	4.59 (3.32, 6.53)	−2.73 (−2.96, −2.49)	29.51 (6.41, 61.97)
15–49 years	11,911 (8,643,15,610)	10.66 (7.74,13.98)	15,298 (11,548,21,448)	5.58 (4.21,7.82)	−2.61 (−2.80,−2.42)	28.44 (10.25,59.73)
Low-middle SDI						
15–19 years	2,065 (1,504, 2,827)	3.51 (2.56, 4.81)	1,994 (1,455, 2,793)	2.21 (1.61, 3.09)	−2.35 (−2.70, −2.00)	−3.42 (−21.37, 20.56)
20–24 years	2,205 (1,574, 2,959)	4.23 (3.02, 5.67)	2,273 (1,584, 3,170)	2.61 (1.82, 3.64)	−2.42 (−2.77, −2.07)	3.07 (−17.41, 30.57)
25–29 years	2,510 (1,697, 3,234)	5.59 (3.78, 7.21)	2,697 (1,912, 3,663)	3.32 (2.35, 4.51)	−2.31 (−2.65, −1.98)	7.46 (−11.72, 37.10)
30–34 years	2,076 (1,426, 2,688)	5.55 (3.81, 7.18)	2,449 (1,765, 3,285)	3.31 (2.39, 4.44)	−2.37 (−2.66, −2.07)	17.94 (−4.74, 46.54)
35–39 years	1,506 (1,092, 2,030)	4.70 (3.41, 6.34)	1,920 (1,405, 2,604)	2.88 (2.11, 3.91)	−2.14 (−2.41, −1.86)	27.46 (3.05, 60.32)
40–44 years	1,582 (1,075, 2,097)	6.10 (4.14,8.08)	2,023 (1,409,2,667)	3.51 (2.44,4.62)	−2.21 (−2.44,−1.98)	27.89 (0.37, 62.23)
45–49 years	1,058 (783,1,419)	4.88 (3.61,6.54)	1,441 (1,100, 1,877)	2.91 (2.23,3.80)	−2.25 (−2.53,−1.97)	36.17 (10.55, 66.43)
15–49 years	13,003 (9,921, 16,480)	4.76 (3.64, 6.04)	14,798 (10,894, 19,707)	2.92 (2.15, 3.89)	−2.25 (−2.55, −1.95)	13.80 (−1.28, 33.83)
Middle SDI						
15–19 years	945 (699, 1,290)	1.03 (0.76, 1.40)	768 (542, 1,093)	0.87 (0.62, 1.24)	−0.91 (−1.12, −0.69)	−18.72 (−28.55, −6.68)
20–24 years	1,211 (889, 1,702)	1.37 (1.01,1.93)	1,018 (700, 1,488)	1.17 (0.81, 1.72)	−1.07 (−1.36, −0.77)	−15.88 (−27.75, −3.73)
25–29 years	1,373 (1,001, 1,852)	1.83 (1.34,2.47)	1,260 (891, 1,830)	1.39 (0.98, 2.02)	−1.33 (−1.65, −1.02)	−8.22 (−21.67, 9.29)
30–34 years	1,165 (859, 1,551)	1.94 (1.43,2.58)	1,307 (950, 1,850)	1.32 (0.96, 1.87)	−1.43 (−1.66, −1.19)	12.18 (−4.56, 30.65)
35–39 years	937 (725, 1,191)	1.69 (1.31,2.15)	1,131 (861, 1,479)	1.23 (0.94, 1.61)	−1.39 (−1.67, −1.11)	20.78 (5.29, 41.55)
40–44 years	831 (613, 1,059)	1.97 (1.45,2.51)	1,121 (886, 1,431)	1.37 (1.08, 1.75)	−1.59 (−1.95, −1.24)	34.89 (15.95, 61.49)
45–49 years	608 (459, 779)	1.79 (1.35,2.30)	980 (777, 1,271)	1.21 (0.96, 1.57)	−1.63 (−1.92, −1.34)	61.33 (42.13, 86.51)
15–49 years	7,069 (5,408, 9,199)	1.58 (1.21, 2.06)	7,586 (5,711, 10,241)	1.23 (0.92, 1.66)	−1.24 (−1.49, −0.98)	7.31 (−3.94, 22.14)
High-middle SDI						
15–19 years	136 (108, 184)	0.29 (0.23, 0.39)	78 (57, 111)	0.23 (0.16, 0.32)	−1.27 (−1.48, −1.06)	−42.92 (−50.84, −34.41)
20–24 years	197 (149, 285)	0.41 (0.31, 0.59)	124 (84, 197)	0.35 (0.24, 0.55)	−1.11 (−1.43, −0.79)	−37.00 (−47.47, −24.95)
25–29 years	219 (160, 323)	0.48 (0.35, 0.71)	155 (102, 277)	0.39 (0.25, 0.69)	−1.15 (−1.48, −0.82)	−29.12 (−42.13, −14.37)
30–34 years	244 (192, 326)	0.58 (0.46, 0.78)	229 (168, 350)	0.45 (0.33, 0.68)	−1.29 (−1.56, −1.01)	−6.03 (−20.14, 11.33)
35–39 years	245 (199, 306)	0.62 (0.50, 0.77)	242 (190, 326)	0.49 (0.38, 0.66)	−1.33 (−1.61, −1.05)	−1.18 (−13.42, 11.84)
40–44 years	220 (183,271)	0.71 (0.60, 0.88)	237 (192, 298)	0.52 (0.42, 0.65)	−1.70 (−2.09, −1.31)	7.87 (−5.09, 23.19)
45–49 years	187 (156, 226)	0.76 (0.64, 0.92)	259 (213, 329)	0.54 (0.44, 0.68)	−1.71 (−2.15, −1.28)	38.49 (21.88, 58.11)
15–49 years	1,448 (1,179, 1,876)	0.52 (0.42, 0.68)	1,325 (1,034, 1,855)	0.43 (0.34, 0.61)	−1.15 (−1.46, −0.84)	−8.53 (−17.95, 1.66)
High SDI						
15–19 years	43 (32, 62)	0.13 (0.10, 0.19)	26 (16, 44)	0.09 (0.06,0.15)	−1.39 (−1.55,−1.24)	−38.99 (−49.44, −29.00)
20–24 years	77 (53, 122)	0.23 (0.16, 0.36)	56 (35, 101)	0.18 (0.11, 0.32)	−0.82 (−0.95, −0.68)	−26.49 (−36.73, −15.37)
25–29 years	85 (52, 157)	0.24 (0.14, 0.44)	70 (38, 140)	0.20 (0.11, 0.41)	−0.51 (−0.60, −0.43)	−17.57 (−28.52, −6.32)
30–34 years	118 (84, 184)	0.33 (0.24, 0.52)	94 (61, 161)	0.25 (0.16,0.43)	−0.87 (−0.96, −0.78)	−19.85 (−28.72, −9.35)
35–39 years	113 (86, 162)	0.34 (0.26, 0.49)	95 (67, 148)	0.25 (0.18, 0.39)	−1.11 (−1.20, −1.03)	−15.61 (−23.89, −5.84)
40–44 years	108 (85, 142)	0.35 (0.27, 0.45)	95 (70, 137)	0.26 (0.19, 0.37)	−1.15 (−1.22, −1.07)	−12.08 (−19.88, −2.76)
45–49 years	110 (92, 137)	0.44 (0.36, 0.54)	111 (86, 151)	0.31 (0.24, 0.42)	−1.16 (−1.25, −1.06)	1.25 (−7.52, 10.83)
15–49 years	653 (493, 949)	0.29 (0.22, 0.42)	549 (385, 866)	0.23 (0.16, 0.36)	−0.91 (−0.98, −0.83)	−16.00 (−22.46, −8.59)

WCBA, women of childbearing age; DALYs, disability-adjusted life years; CI, confidence interval; EAPC, estimated annual percentage change; UI, uncertainty interval; SDI, sociodemographic index.

Across the 5 SDI regions, the percentage change in syphilis cases among WCBA showed diverse trends with age. In low SDI regions, the percentage change in the prevalence, incidence, and DALYs of syphilis among WCBA demonstrates a consistent upward trajectory across all age groups, with percentage changes in incidence ranging from approximately 70 to 100%, in prevalence from around 60 to 90%, and in DALYs from roughly 20 to 30%. Moreover, as the SDI level shifted from low-middle to high, the percentage change in syphilis cases showed a gradual increase with advancing age, with incident cases in the high-middle SDI region rising from −11.61% in the 15–19 age group to 125.58% in the 45–49 age group. However, with improved economic conditions, the percentage change in each age group gradually decreases, with incident cases in the 45–49 age group declines from 104.71% in low SDI to 40.12% in high SDI regions. It is important to note that the surge in syphilis cases among the 45–49 age group in high-middle SDI regions has resulted in percentage changes in incidence and prevalence peaking at 125.58 and 122.59%, respectively, while the peak for DALYs in middle SDI regions is 61.33% (Table 2; Figure 4c; Supplementary Figure S6).

The trend analysis of global incidence, prevalence, and DALY rates among WCBA from 1990 to 2021 reveals a decreasing trend across all age groups, with the most significant data observed in the 45–49 age group, showing an EAPC of −0.55 (95% CI: −0.72 to −0.39) for incidence, −0.72 (95% CI: −0.88 to −0.56) for prevalence, and −1.78 (95% CI: −2.04 to −1.53) for DALYs. In the 5 SDI regions, all age groups exhibit a declining trend in the EAPC for incidence and prevalence rates, with the exception of the high-middle SDI region, where both incidence and prevalence rates demonstrate an upward trend; furthermore, the EAPC for DALYs across all age groups in the high-middle SDI region also reflects a decreasing trend (Table 2; Figure 4d; Supplementary Figure S6).

In 2021, the incidence of syphilis among WCBA was recorded at 877.72 thousand (95% UI: 455.42 to 1436.36) for the age group 15–19 and 1.37 million (95% UI: 0.76 to 2.21) for the age group 20–24, with the total number of cases in these two cohorts accounting for approximately one-quarter of the global total. Meanwhile, prevalence cases were reported as 2.40 million (95% UI: 1.71 to 3.36) for the age group 15–19 and 4.01 million (2.25 to 6.23) for the age group 20–24, collectively representing about one-third of the global total. Notably, from 1990 to 2021, the most rapid increases in the incidence and prevalence rates of syphilis among WCBA in high-middle SDI regions were observed in the 15–19 age group, with an EAPC of 0.82 (95% CI: 0.64 to 1.01) for incidence and 0.62 (95% CI: 0.46 to 0.78) for prevalence, indicating that this age group bore the highest new burden of syphilis among WCBA globally in 2021 (Table 2; Figure 4d; Supplementary Figure S6e).

Between 1990 and 2021, the prevalence and incidence of syphilis among WCBA exhibited a decline across the age cohorts of 15–19, 20–24, and 25–29 years globally. Conversely, there was an observed increase in the age groups of 30–34, 35–39, 40–44, and 45–49 years. Within the five SDI regions, the trends in proportions from low-middle SDI to high SDI regions across various age groups mirrored the global trend, with no discernible changes observed in the low SDI region.

Among the 21 regions, East Asia demonstrated the most significant increase in the proportion of syphilis cases among WCBA in the 45–49 age group from 1990 to 2021, with the proportion of incidence cases rising from 7 to 14%, prevalence increasing from 7 to 14%, and DALYs growing from 13 to 22%. Concurrently, this region experienced a substantial decrease in the proportion of cases within the 15–19 age group, with a decline ranging between 40 and 60%. Consequently, there was an observable trend indicating a shift in the distribution of syphilis cases among WCBA from younger age groups to older cohorts (Figure 4b; Supplementary Figures S6a,b).

### 3.6 The association between syphilis burden and SDI

**Figure 5:**
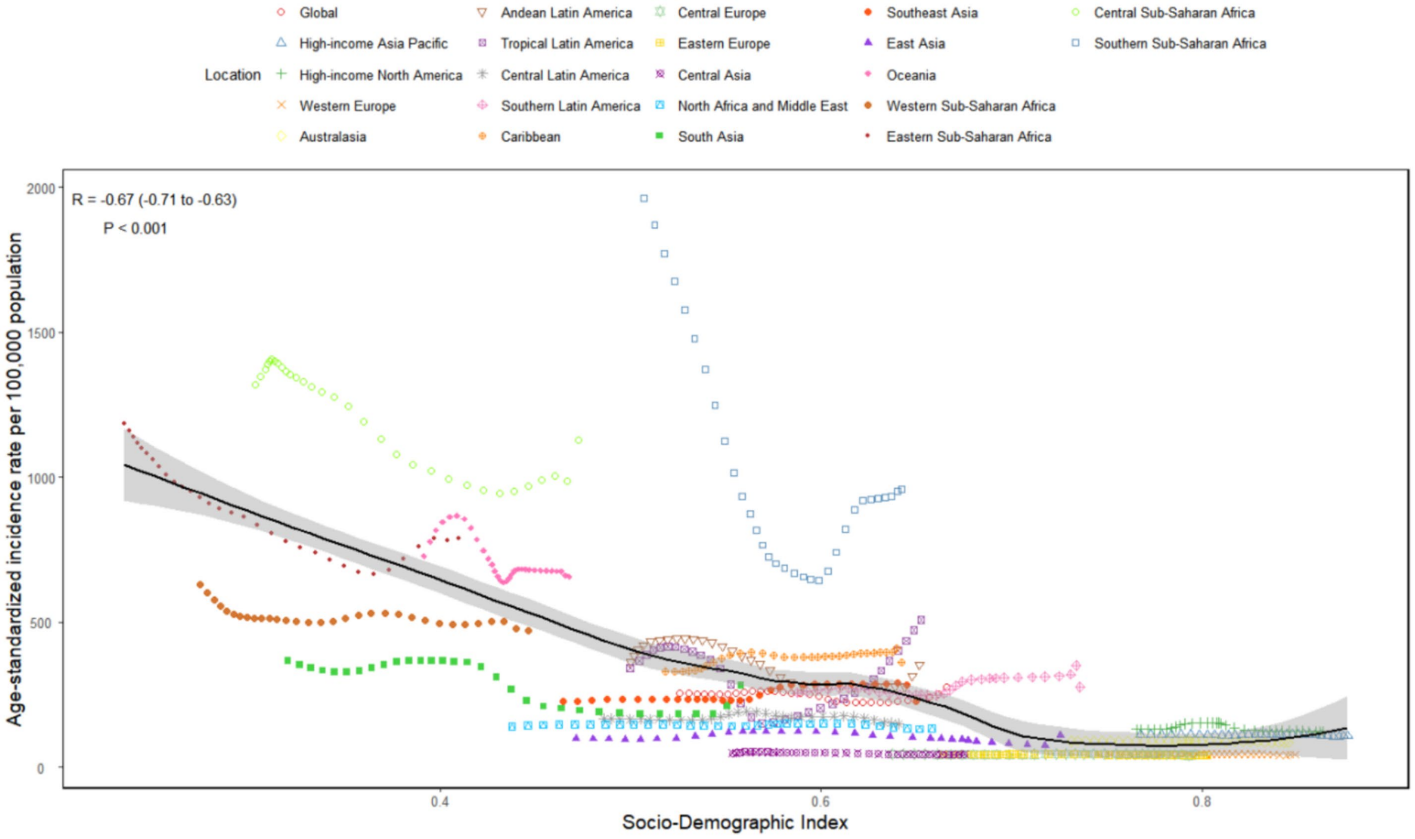
The associations between the SDI and ASIR per 100,000 population of syphilis in WCBA across 21 GBD regions. WCBA, women of childbearing age; SDI, sociodemographic index; GBD, Global Burden of Disease; ASIR, age-standardized incidence rate.

**Figure 6:**
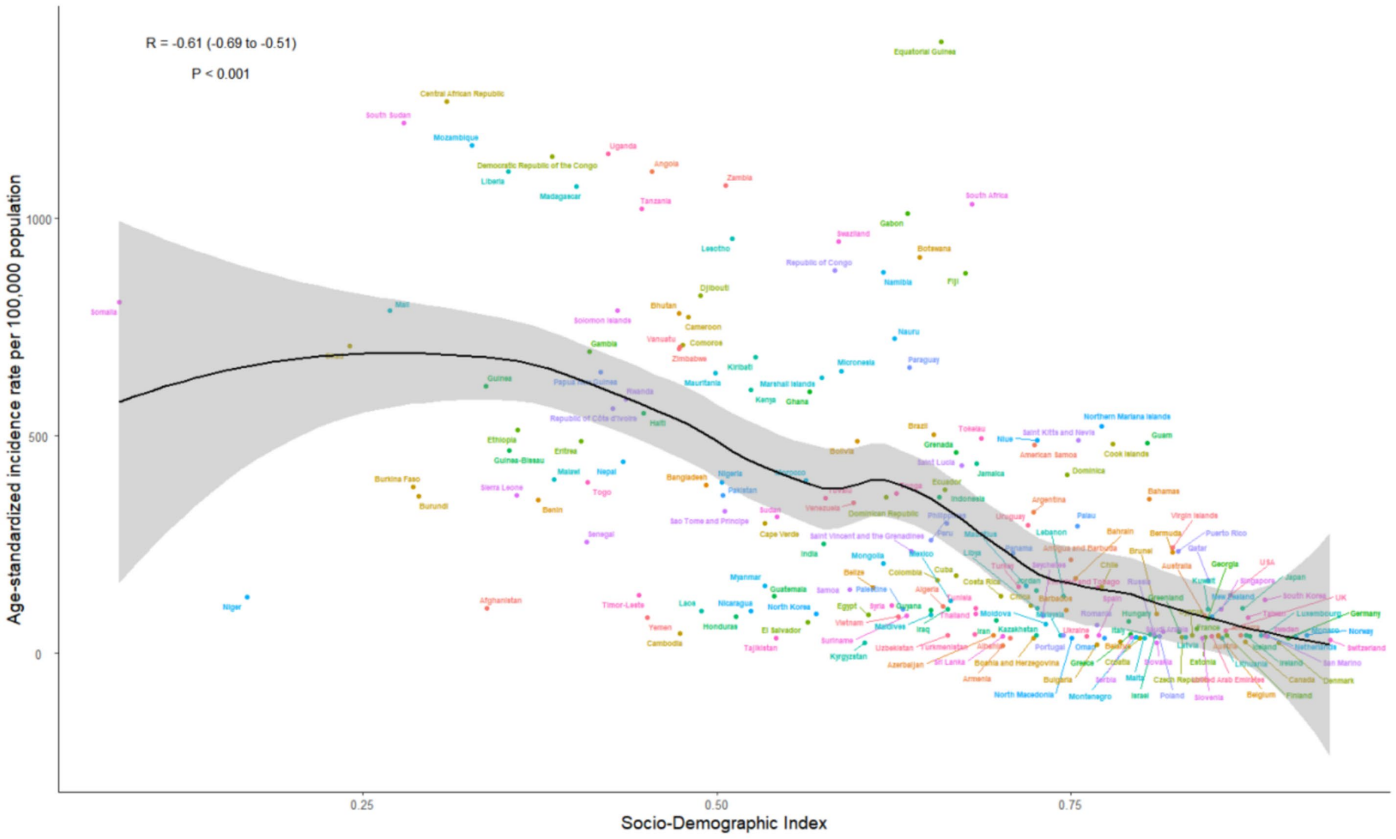
The associations between the SDI and ASIR per 100,000 population of syphilis in WCBA across 204 countries. WCBA, women of childbearing age; SDI, sociodemographic index; ASIR, age-standardized incidence rate.

In 2021, a negative correlation was identified between the age-standardized prevalence, incidence, and DALYs rates of syphilis among WCBA and the SDI. With economic advancements, the overall disease burden has been diminishing; nonetheless, the global syphilis burden remains slightly above projected levels for both prevalence and incidence, and DALYs also marginally surpass expected figures. In 21 regions, the syphilis burden among WCBA experiences a significant reduction when the SDI dips below 0.6. The burden attains its nadir as the SDI nears 0.8. Notably, regions such as Southern Sub-Saharan Africa and Central Sub-Saharan Africa exhibit a burden that exceeds projections, whereas regions including Central Asia, North Africa and the Middle East, Central Latin America, and South Asia display a burden that falls below anticipated levels. In several nations, the burden of syphilis is higher than anticipated in Equatorial Guinea, Central African Republic, Zambia, and South Sudan; conversely, it is lower than expected in Niger, Afghanistan, Yemen, and Laos (Figures 5, 6; Supplementary Figure S7).

### 3.7 The influential factors for EAPC

**Figure 7:**
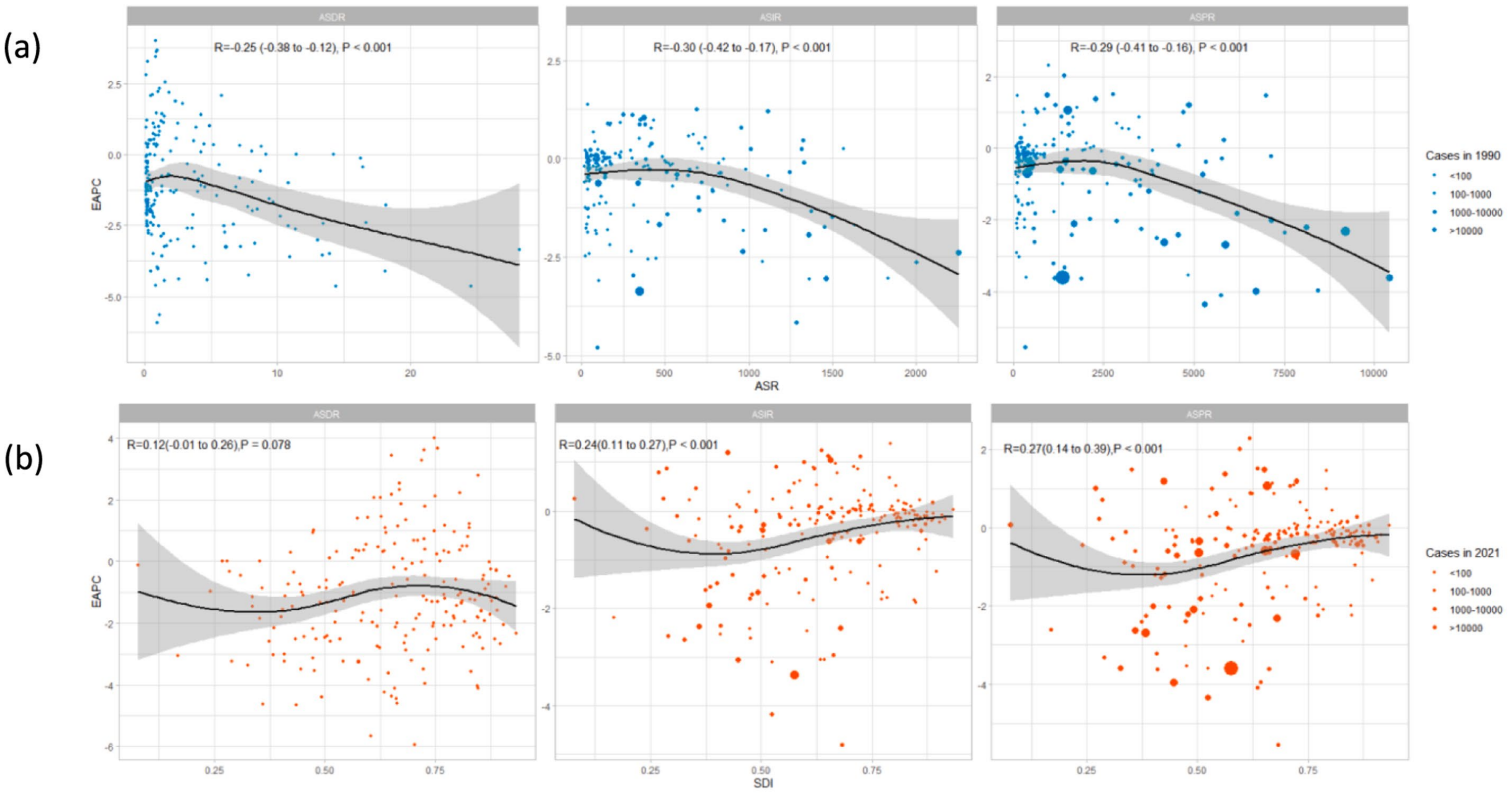
The associations between EAPC and ASR in 1990, and SDI in 2021: **(a)** The correlation between EAPC and ASIR, ASPR, ASDR in 1990 across 204 countries; **(b)** The correlation between EAPC and SDI in 2021 across 204 countries. WCBA, women of childbearing age; EAPC, estimated annual percentage change; SDI, sociodemographic index; ASR, age-standardized rate; ASIR, age-standardized incidence rate; ASPR, age-standardized prevalence rate; ASDR, age-standardized DALYs rate.

Figures 7a,b illustrate a significant association between the EAPC and ASR for incidence, prevalence, and DALYs in 1990, as well as the SDI values in 2021. The age-standardized incidence, prevalence, and DALYs rates for syphilis in 1990 indicate the baseline disease reservoir, whereas the SDI of 2021 acts as a proxy for healthcare access and quality in each nation. A significant negative association was observed between EAPC and ASR, with correlation coefficients of R = −0.30 (*p* < 0.001) for age-standardized incidence rate (ASIR), R = −0.29 (*p* < 0.001) for age-standardized prevalence rate (ASPR), and R = −0.25 (*p* < 0.001) for age-standardized DALYs rate (ASDR). In contrast, a significant positive relationship was identified between EAPC and SDI, with correlation coefficients of R = 0.24 (*p* < 0.001) for ASIR and R = 0.27 (*p* < 0.001) for ASPR, while there was no significant relationship for ASDR (*p* > 0.05). Nations with a SDI between 0.25 and 0.75 have seen a faster decline in ASR of syphilis from 1990 to 2021 (Figures 7a,b).

## 4 Discussion

The WHO declared the EMTCT of syphilis a public health priority in 2007 (14), while also launching its Global Health Sector Strategy on Sexually Transmitted Infections for 2016–2021 and the Global Health Sector Strategies on HIV, Viral Hepatitis, and Sexually Transmitted Infections for the period 2022–2030 in 2016 and 2022 (7, 24), respectively. These strategies aim to end epidemics and promote universal health coverage, ensuring access to high-quality, evidence-based health services (25). Critical to these global efforts are targeted prevention strategies, including comprehensive sexual health education, expanded access to antenatal syphilis screening, and effective treatment of pregnant women diagnosed with syphilis. Several countries have implemented innovative local initiatives to address syphilis prevention within specific communities. For instance, the province of Ontario in Canada has effectively established a community-based outreach program that offers on-site syphilis testing and treatment in underserved regions (26). These localized strategies frequently integrate culturally sensitive methods and tackle specific barriers to healthcare access, thereby showcasing a dedication to customized interventions designed to maximize effectiveness. Further research is required to provide more comprehensive insights into these local prevention strategies. Women, particularly those of childbearing age from low-and middle-income countries, are central to these efforts, as syphilis damages reproductive health and leads to APOs, endangering infants and young children (27). A thorough understanding of the prevalence trends of syphilis in WCBA is crucial for assessing the potential to achieve related health goals, driven by the urgent need to address the rising burden of syphilis among this population globally. Currently, there is a lack of comprehensive analyses on the prevalence, incidence, and DALYs related to syphilis in WCBA across different countries and regions globally, as previous studies have primarily focused on analyzing all STIs or syphilis in the context of global changes (4, 5, 12, 27, 28). The prompt enhancement and regular updating of statistics regarding the burden of syphilis among the global population of WCBA are crucial for effective prevention, control, and treatment, thereby enabling policymakers to comprehend the situation and devise appropriate strategies. This study offers the first comprehensive estimation of the prevalence, incidence, and DALYs associated with syphilis in WCBA over the past 32 years utilizing GBD 2021 database on a global scale, while also providing an in-depth analysis of syphilis trends from 1990 to 2021 to elucidate contributing factors and identify regions and demographics that necessitate immediate attention.

From a global perspective, the overall trends in the incidence, prevalence, and DALYs of syphilis among WCBA reveal a complex interplay of factors contributing to both increases and decreases. The observed increase in the absolute numbers of new syphilis cases, prevalent cases, and DALYs among WCBA globally is a matter of concern, with a percentage change of 46.94, 45.85, and 16.08%, respectively, over the past 32 years. This growth may be associated with the global population increase of 45% (29), which is consistent with recent studies (30, 31). While the increasing trend in syphilis cases may be attributed to factors such as heightened sexual risk behaviors, enhanced global syphilis screening and detection efforts, and unequal distribution of medical resources (32). Conversely, the decline in ASR for incidence, prevalence, and DALYs, possibly attributed to a decrease in the proportion of WCBA in the global population from 25.57% in 1990 to 24.67% in 2021 (29), indicates progress in the management and control of syphilis, likely resulting from enhanced awareness, improved healthcare infrastructure, better access to healthcare, and the implementation of targeted prevention efforts and more effective treatment protocols.

An examination of syphilis trends within the five SDI regions offers additional perspective on the disparities in disease burden. The variance in syphilis burden across the SDI regions is notable, with the low-middle SDI region experiencing the highest number of new and existing cases of syphilis. Conversely, the regions with low SDI sustain the most substantial burden in terms of DALYs. These trends highlight the impact of socio-economic factors on health outcomes, as regions with lower SDI often face challenges such as limited access to healthcare, inadequate health education, and higher rates of poverty, all of which contribute to the sustained high burden of syphilis; this pattern is consistent with the broader trend of infectious diseases being more prevalent in settings with limited resources and less developed healthcare systems (33). In contrast, while high-middle SDI regions have generally benefited from better healthcare access and resources that allow for more effective management of syphilis cases (8), the observed upward trend in incidence and prevalence may reflect changes in sexual behavior, increased testing, or other sociocultural factors that warrant further investigation (34). This dichotomy underscores the importance of developing tailored public health strategies that address the unique challenges and social determinants of health specific to different SDI regions in order to effectively tackle the disparities they face.

At the level of individual countries and the 21 analyzed regions, the trends in syphilis burden reveal significant variations. The variation in syphilis trends across GBD regions emphasizes the importance of regional-specific strategies. The decrease in syphilis burden in high SDI regions like High-Income Asia Pacific and Western Europe may be attributed to effective public health interventions and economic development (35). Conversely, the increase in regions such as Southern Latin America, Southeast Asia, and the Caribbean suggests a need for intensified prevention and control efforts, particularly in light of the potential for syphilis to spread in the context of limited healthcare resources and infrastructure (36). Furthermore, approximately 60% of countries reported increasing trends in syphilis cases, particularly in high SDI nations like Qatar and Kuwait, where increases exceeded 500%. This paradox indicates that even in regions with robust healthcare systems, factors such as increased sexual activity, social stigma surrounding testing, and underreporting may contribute to rising cases (37). In contrast, countries like Georgia and Latvia, despite their high SDI status, experienced decreases in syphilis cases, likely due to effective public health campaigns, a finding that aligns with the study by Fu et al. (4). These mixed trends highlight the heterogeneity in syphilis control and the need for localized strategies, continuous monitoring, and evaluation of public health initiatives (12), particularly as a notable polarization trend emerges among countries in high SDI regions, with differences reaching up to seven-fold between some nations. Countries experiencing significant increases may need to reevaluate their prevention strategies, while those with decreasing trends provide valuable lessons on effective control measures that could be adapted elsewhere.

Age-specific trends in syphilis incidence, prevalence, and DALYs reveal significant shifts in the disease’s demographic profile, particularly an increase in cases among older age groups, especially those aged 45–49. Additionally, there is a trend of transition in the proportion of syphilis cases among WCBA from younger age groups to older age groups, with the most significant increase observed in the 45–49 age group. This trend may be influenced by changing sexual behaviors, increased longevity, and the potential resurgence of syphilis in populations previously deemed at lower risk (38). Conversely, the decline in younger age groups suggests that public health initiatives targeting adolescents and young adults are yielding positive results (39). However, the rising burden among older populations necessitates a reevaluation of prevention strategies to ensure they are inclusive and address the needs of all age groups. Notably, the age patterns observed among WCBA in high-middle SDI regions further underscore the importance of considering these trends when designing targeted interventions and allocating resources to ensure effective prevention efforts across all demographics.

The correlation between ASR for incidence, prevalence, and DALYs and SDI underscores the significant impact of socio-economic conditions on the burden of syphilis, with regions and countries exhibiting lower SDI values tending to experience higher rates of the disease. This indicates that socio-economic factors play a crucial role in influencing health outcomes (16). The unexpected burden of syphilis in certain nations, such as Equatorial Guinea and the Central African Republic, highlights the necessity for targeted interventions and resource allocation (40). Conversely, nations with higher SDI values, where the burden is lower than anticipated, may benefit from comprehensive healthcare systems that provide better access to preventive services and treatment (27). While the negative correlation between syphilis burden and SDI emphasizes the role of economic development in reducing disease incidence and prevalence, the persistently high global burden of syphilis in some regions suggests that economic development alone is insufficient for effective control. Therefore, additional factors, such as health education, healthcare infrastructure, and public health policies, must be considered in the development of comprehensive syphilis control strategies (41).

We identified that the extent of variations in ASR, namely EAPC, during the period from 1990 to 2021 was significantly negatively related to baseline ASR, elucidating the dynamics of syphilis tendencies. For those nations having low ASR in 1990, the likelihood of syphilis growth was greater. This outcome can be accounted for as follows: Firstly, the lower the baseline ASR, the more significant the variation in ASR, namely the reservoir effect. Secondly, countries with low ASR were not as likely to regard syphilis as a top priority in disease prevention programs because of its restricted public health significance in comparison with other public health matters, which demonstrated the importance of the baseline disease reservoir and healthcare accessibility in determining the course of the syphilis burden. Furthermore, countries with higher baseline ASR and lower SDI values experienced a more rapid rise in the syphilis burden, indicating the necessity of targeted interferences in these circumstances. On the contrary, countries with lower baseline ASR and higher SDI values presented a decrease in the syphilis burden, suggesting that enhancements in healthcare accessibility and quality could be conducive to syphilis control.

## 5 Limitation

This study is confronted with several limitations. Primarily, the dearth of comprehensive data hindered our ability to conduct a thorough analysis of syphilis trends. Not all syphilis cases are reported to health authorities since many physicians might not consistently diagnose or report the disease. Moreover, syphilis is often referred to as a “master of disguise” due to its varied and atypical symptoms, which can imitate various other diseases. This characteristic may lead to misdiagnosis by less-experienced healthcare providers, further contributing to underreporting. Consequently, the true burden of syphilis among WCBA may be underestimated, limiting the precision of our findings and the effectiveness of public health interventions aimed at tackling this critical issue.

## 6 Conclusion

The overall burden of syphilis among WCBA in terms of ASR for incidence, prevalence, and DALYs has significantly increased globally over the past 32 years. This rise is particularly pronounced in low-and middle-SDI regions, highlighting disparities in access to healthcare and preventive services. The analysis indicates that socio-economic factors play a crucial role in the distribution of syphilis, with regions of lower SDI facing the highest burdens. Additionally, the demographic shift shows a troubling increase in cases among older age groups, necessitating a reevaluation of current public health strategies to encompass all age demographics. To effectively address these challenges, urgent interdisciplinary efforts are needed, including enhanced education, targeted policies, and improved healthcare access. Meeting the Sustainable Development Goals requires a concerted investment in syphilis prevention and treatment resources for WCBA, particularly in underserved regions. By prioritizing these actions, we can work toward reducing the incidence of syphilis and ultimately improving maternal and child health outcomes globally.

## Data Availability

The original contributions presented in the study are included in the article/Supplementary material, further inquiries can be directed to the corresponding author.

## Footnotes

^1^ https://vizhub.healthdata.org/gbd-results/
