# Supplementary material for: Global, regional, and national burden and trends of syphilis among women of childbearing age from 1990 to 2021
Source: Front Public Health. 2025 Jul 8;13:1580964. doi: 10.3389/fpubh.2025.1580964 (PMC12279697; doi:10.3389/fpubh.2025.1580964)
Supplement: Supplementary file 1 [file Data_Sheet_1.docx]

**Supplementary Document**

*Global trends and burden of syphilis among women of childbearing age from 1990 to 2021*

*Huang et al.*

**Content**

[**Supplementary Texts 3**](#_Toc2970)

[Supplementary Methods Detailed process description for extracting data from the GBD 2021 database. 3](#_Toc1113)

[**Supplementary Figures 4**](#_Toc7996)

[Fig.S1 The logical flowchart of data extraction and analysis for this study 4](#_Toc24223)

[Fig.S2 The cluster dendrogram based on EAPC prevalence, incidence, and DALYs in the countries or territories 5](#_Toc20993)

[Fig.S3 Temporal trend of syphilis burden in WCBA in global and regions 6](#_Toc32300)

[Fig.S4 Temporal trend of syphilis prevalence in WCBA globally 7](#_Toc21990)

[Fig.S5 Temporal trend of syphilis burden in WCBA globally 8](#_Toc11387)

[Fig.S6 Temporal trend of syphilis burden in WCBA by age pattern in different regions 9](#_Toc30757)

[Fig.S7 The association between syphilis burden and SDI 10](#_Toc11484)

[**Supplementary Tables 1**](#_Toc30147)**1**

[Table S1 The incidence of syphilis cases and rates among WCBA in 1990 and 2021 across 204 countries, and the trends from 1990 to 2021 1](#_Toc6995)1

[Table S2 The prevalence of syphilis cases and rates among WCBA in 1990 and 2021 across 204 countries, and the trends from 1990 to 2021 1](#_Toc18825)9

[Table S3 The DALY of syphilis cases and rates among WCBA in 1990 and 2021 across 204 countries, and the trends from 1990 to 2021 2](#_Toc17195)8

**Supplementary Texts**

**Supplementary Methods** Detailed process description for extracting data from the GBD 2021 database.

To extract the raw data, we need to follow a process. First, click on the website above to enter the data retrieval interface, where different GBD evaluation options can be selected from the drop-down menu in "GBD Estimate". By default, " Cause of death or injury" is selected, in which specific options can be chosen according to the content of your study. To investigate the current status and temporal trends of syphilis among WCBA, we extracted estimates and 95% Uncertainty Intervals (UI) for syphilis incidence, prevalence, and DALYs across seven age categories (15-19, 20-24, 25-29, 30-34, 35-39, 40-44, and 45-49 years) at the global level, as well as across 5 SDI regions and 204 countries and territories, using data from the GBD 2021. In the "Metric" drop-down menu, we can select different measures, such as Number, Rate.The analysis was based on a study period from 1990 to 2021, with the location criteria including "Global," the 21 designated geographic locations, SDI areas, and the 204 countries or territories. The focus was specifically on the cause of "syphilis" among females, and we utilized "Incidence," "Prevalence," and "DALYs" as key measure to assess the burden of the disease. After selecting the above conditions, click "Search" to retrieve the information, or you can choose to download the CSV file directly by clicking the "Download CSV" button.

**Supplementary Figures**


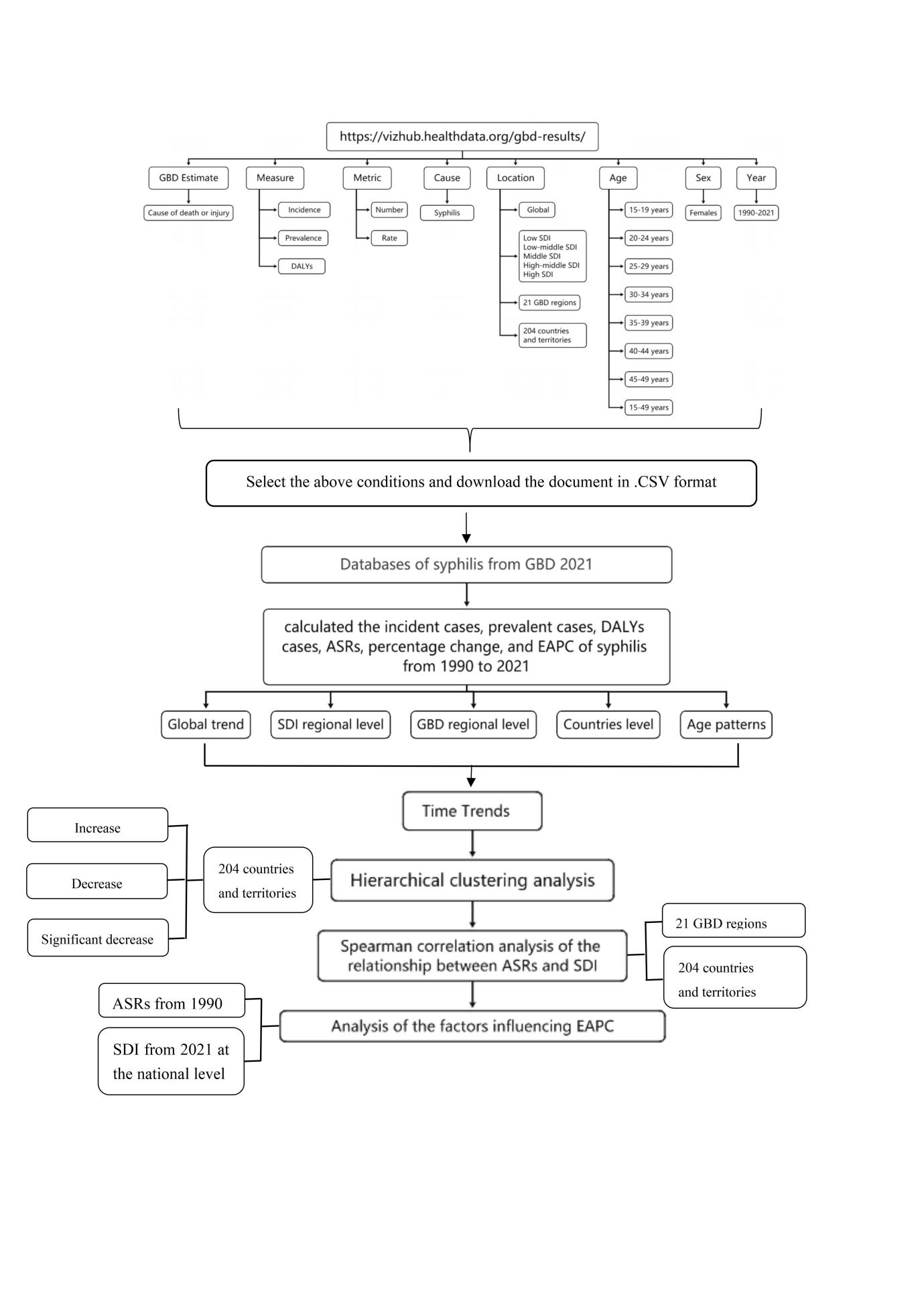


**Fig.S1 The logical flowchart of data extraction and analysis for this study**

**
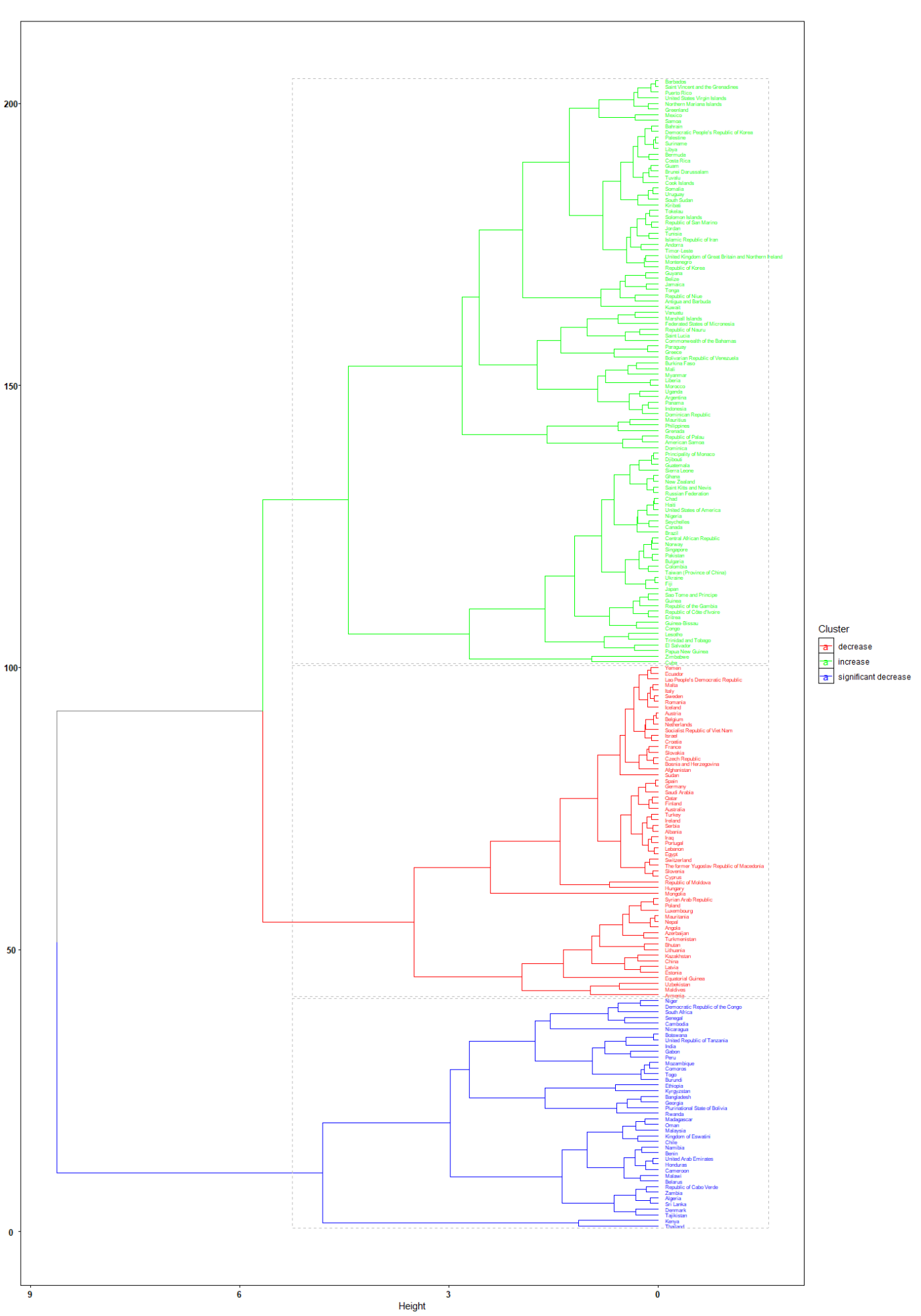
**

**Fig.S2 The cluster dendrogram based on EAPC prevalence, incidence, and DALYs in the countries or territories**


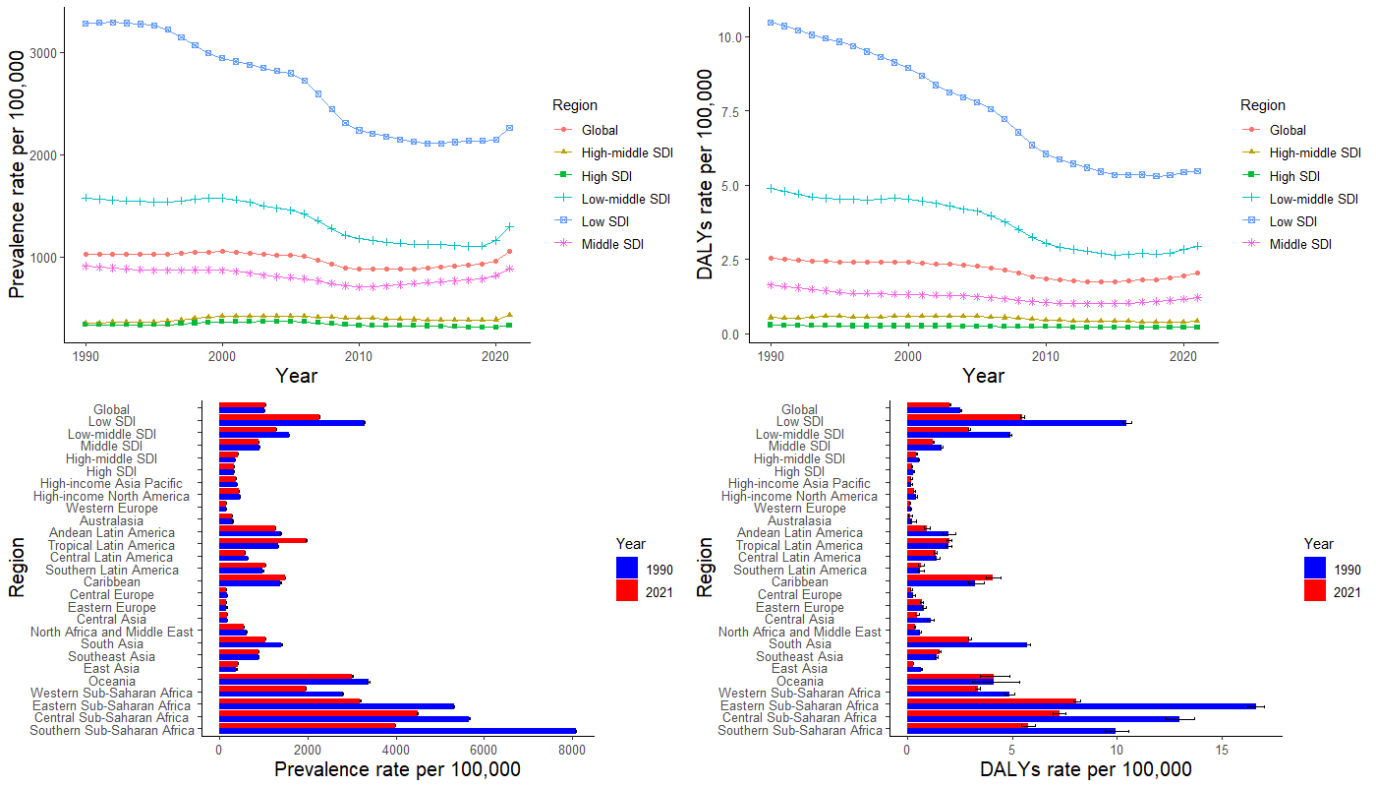


(d)

(c)

(b)

(a)

**Fig.S3 Temporal trend of syphilis burden in WCBA in global and regions:**

**(a)** The rates of prevalence from 1990 to 2021; **(b)** The rates of DALYs from 1990 to 2021; **(c)** Prevalence rate per 100,000 population in 1990 and 2021 among regions; **(d)** DALYs rate per 100,000 population in 1990 and 2021 among regions. WCBA, Women of Childbearing Age; DALYs, Disability-Adjusted Life Years.


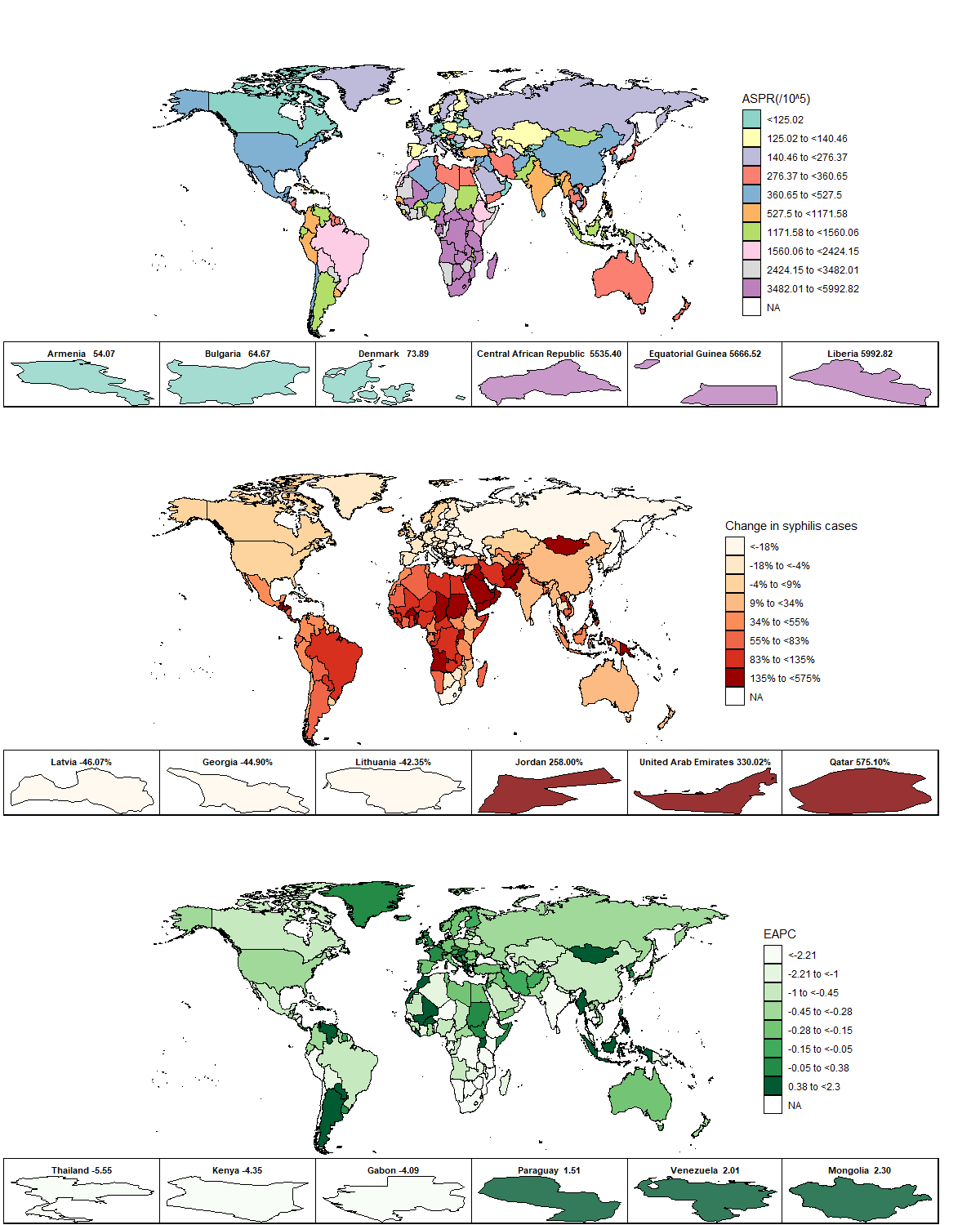


(c)

(b)

(a)

**Fig.S4 Temporal trend of syphilis prevalence in WCBA globally:**

**(a)** Age-Standardized prevalent rate across 204 countries in 2021; **(b)** Percentage change in prevalent cases across 204 countries in 1990 and 2021; **(c)** EAPC in prevalent rates across 204 countries from 1990 to 2021. WCBA, Women of Childbearing Age; EAPC, Estimated Annual Percentage Change; ASPR, Age-Standardized Prevalent Rate.


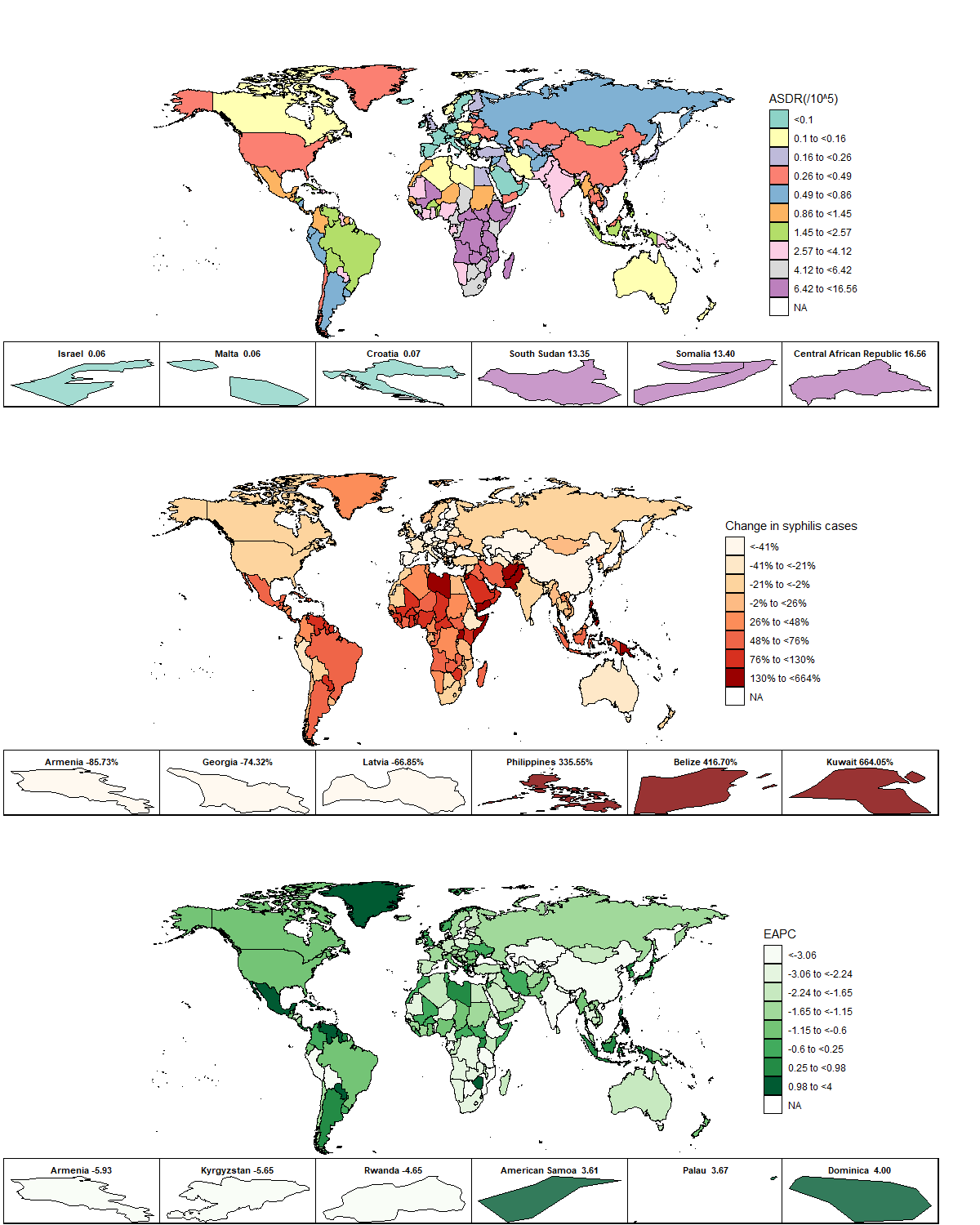


(c)

(b)

(a)

**Fig.S5 Temporal trend of syphilis burden in WCBA globally:**

**(a)** Age-Standardized DALYs rate across 204 countries in 2021; **(b)** Percentage change in DALYs cases across 204 countries in 1990 and 2021; **(c)** EAPC in DALYs rates across 204 countries from 1990 to 2021. WCBA, Women of Childbearing Age; EAPC, Estimated Annual Percentage Change; DALYs, Disability-Adjusted Life Years; ASDR, Age-Standardized DALYs Rate.


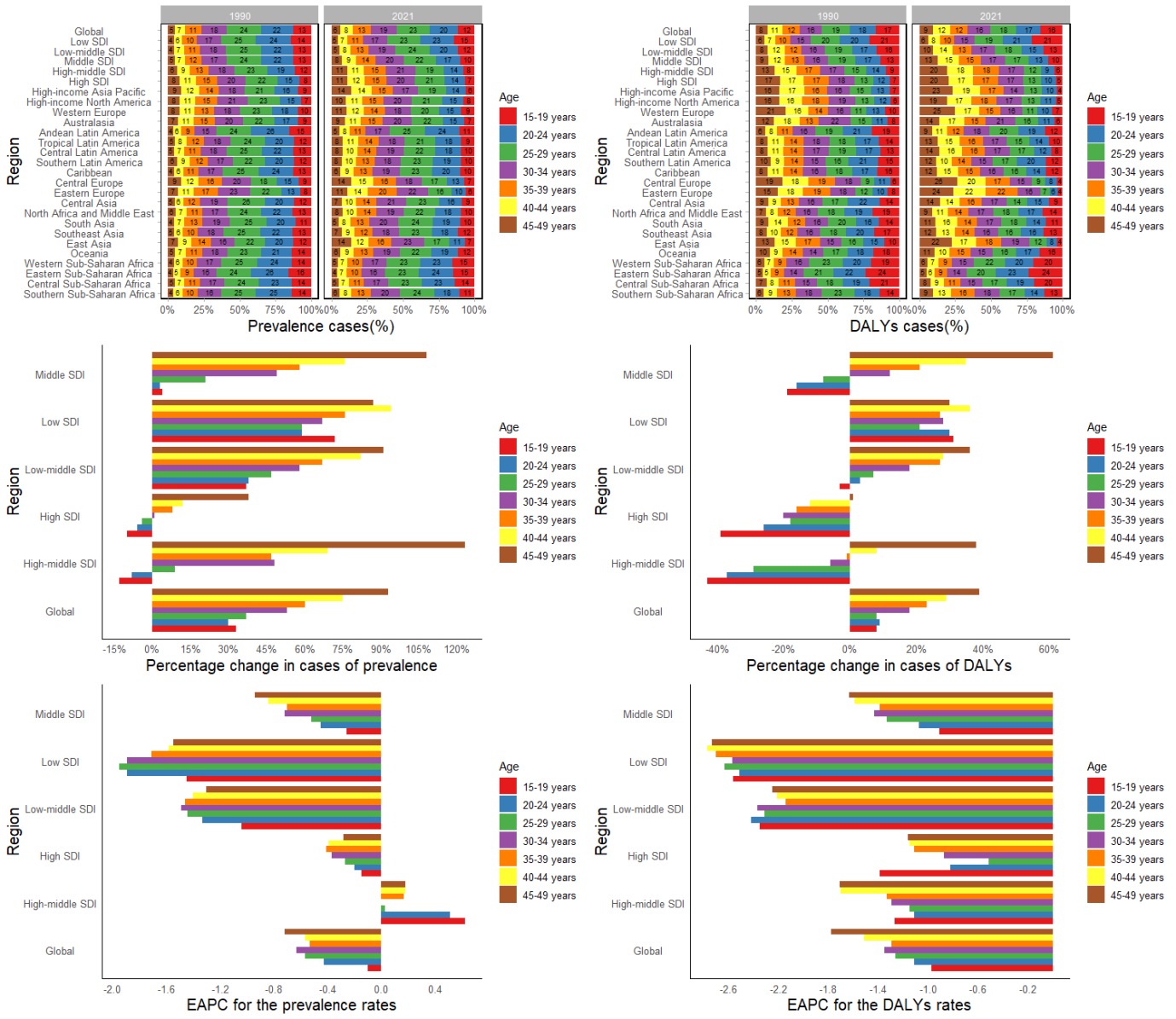


(f)

(e)

(c)

(a)

(d)

(b)

**Fig.S6 Temporal trend of syphilis burden in WCBA by age pattern in different regions:**

**(a)** The distribution of prevalent cases across 7 age groups as percentages globally, in 5 territories, and 21 GBD regions in 1990 and 2021; **(b)** The distribution of DALYs cases across 7 age groups as percentages globally, in 5 territories, and 21 GBD regions in 1990 and 2021; **(c)** Percentage change in prevalent cases of 7 age groups globally and in 5 territories in 1990 and 2021; **(d)** Percentage change in DALYs cases of 7 age groups globally and in 5 territories in 1990 and 2021; **(e)** EAPC of prevalent rates of 7 age groups globally and in 5 territories from 1990 to 2021; **(f)** EAPC of DALYs rates of 7 age groups globally and in 5 territories from 1990 to 2021. WCBA, Women of Childbearing Age; EAPC, Estimated Annual Percentage Change; DALYs, Disability-Adjusted Life Years; SDI, Socio-Demographic Index; GBD, Global Burden of Disease.


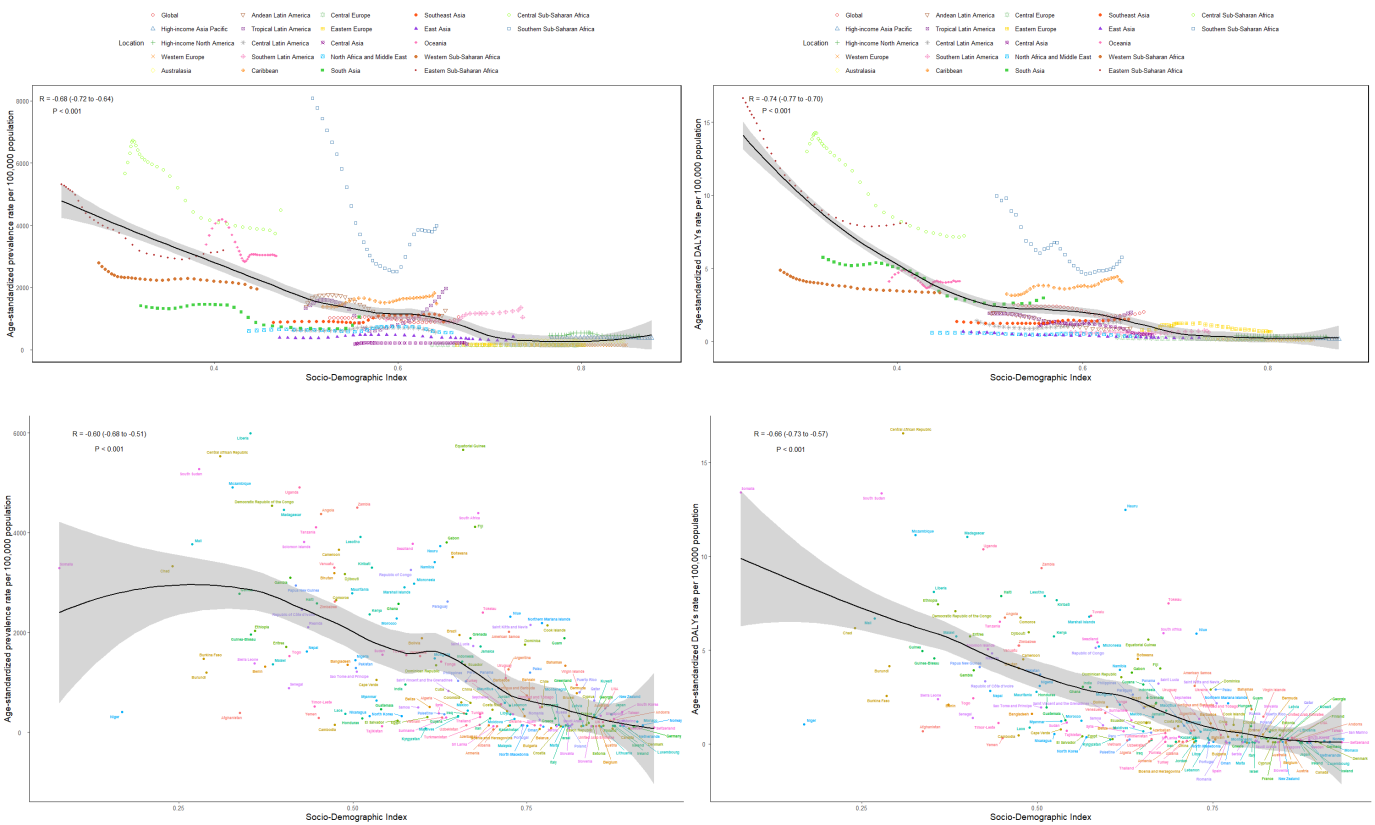


(d)

(b)

(a)

(c)

**Fig.S7 The association between syphilis burden and SDI:**

**(a)** The associations between the SDI and Age-standardized prevalence rates per 100,000 population of syphilis in WCBA across 21 GBD regions; **(b)** The associations between the SDI and Age-standardized DALYs rates per 100,000 population of syphilis in WCBA across 21 GBD regions; **(c)** The associations between the SDI and prevalence rates per 100,000 population of syphilis in WCBA across 204 countries; **(d)** The associations between the SDI and DALYs rates per 100,000 population of syphilis in WCBA across 204 countries. WCBA, Women of Childbearing Age; SDI, Socio-Demographic Index; GBD, Global Burden of Disease.

# Supplementary Tables

## Table S1 The incidence of syphilis cases and rates among WCBA in 1990 and 2021 across 204 countries, and the trends from 1990 to 2021

| **Location** | **1990** | |  | **2021** | |  | **1990-2021** | |
| --- | --- | --- | --- | --- | --- | --- | --- | --- |
|  | **Cases number** | **ASR per 100,000** |  | **Cases number** | **ASR per 100,000** |  | **EAPC** | **Cases change(%)** |
|  | **No.  (95% UI)** | **No. (95% CI)** |  | **No.  (95% UI)** | **No. (95% CI)** |  | **No. (95% CI)** | **No. (95% UI)** |
| American Samoa | 63(44,88) | 484.75(369.27,633.14) |  | 56(39,76) | 479.65(360.66,627.28) |  | 0.04(0.01,0.07) | -12.45(-21.59,-1.59) |
| Antigua and Barbuda | 35(24,48) | 205.34(142.47,291.41) |  | 51(35,67) | 213.82(158.78,282.72) |  | 0.13(0.04,0.21) | 43.44(31.75,57.84) |
| Egypt | 11833(7962,16935) | 86.79(85.21,88.41) |  | 23102(15473,32202) | 86.97(85.85,88.11) |  | 0.10(0.05,0.15) | 95.22(80.48,112.40) |
| Argentina | 24637(18067,32832) | 301.71(297.94,305.51) |  | 38325(26693,51967) | 324.28(321.04,327.54) |  | 1.12(0.69,1.55) | 55.56(39.27,74.82) |
| Australia | 4031(2785,5439) | 90.12(87.36,92.95) |  | 5043(3489,6766) | 85.49(83.13,87.91) |  | -0.21(-0.26,-0.15) | 25.08(15.09,36.22) |
| Barbados | 62(43,83) | 87.20(66.68,112.78) |  | 68(49,89) | 97.77(75.72,124.48) |  | 0.11(0.04,0.18) | 9.47(-2.15,22.53) |
| Belize | 68(47,93) | 149.20(114.04,195.04) |  | 187(130,249) | 149.69(128.84,173.22) |  | -0.17(-0.32,-0.02) | 174.51(152.45,203.72) |
| Bermuda | 40(28,54) | 233.74(166.30,322.25) |  | 29(20,39) | 232.01(153.41,339.47) |  | 0.03(0.00,0.06) | -28.07(-35.15,-19.91) |
| Venezuela | 18228(12479,24884) | 351.27(346.07,356.55) |  | 22642(15949,30990) | 344.65(340.14,349.21) |  | 1.00(0.70,1.30) | 24.22(10.67,41.53) |
| Bosnia and Herzegovina | 402(276,542) | 34.01(30.76,37.54) |  | 248(174,340) | 34.98(30.69,39.74) |  | 0.11(0.09,0.13) | -38.28(-44.46,-31.50) |
| Brunei | 67(46,89) | 92.95(71.34,120.97) |  | 114(79,149) | 90.72(74.74,109.32) |  | -0.12(-0.15,-0.08) | 70.20(54.34,89.61) |
| Burkina Faso | 8380(5819,11599) | 380.20(371.84,388.72) |  | 22684(15199,30466) | 382.76(377.61,387.99) |  | 0.88(0.57,1.18) | 170.70(140.57,215.79) |
| Canada | 1923(1348,2596) | 26.02(24.86,27.22) |  | 2048(1453,2752) | 25.13(24.04,26.25) |  | -0.59(-1.35,0.18) | 6.48(-0.43,15.68) |
| Central African Republic | 9137(6331,12418) | 1329.96(1301.82,1358.65) |  | 18877(13104,25609) | 1269.23(1250.68,1288.02) |  | -0.10(-0.37,0.16) | 106.60(89.75,124.74) |
| Dominica | 75(51,101) | 408.18(318.34,518.78) |  | 68(47,93) | 409.61(317.88,520.23) |  | 0.32(0.19,0.45) | -9.06(-19.00,-0.53) |
| The Bahamas | 268(185,364) | 344.86(304.05,390.50) |  | 377(266,509) | 353.86(318.99,391.56) |  | 0.51(0.33,0.70) | 40.74(27.53,56.83) |
| Cook Islands | 24(17,33) | 501.30(319.13,762.45) |  | 21(14,28) | 480.83(295.91,743.95) |  | -0.10(-0.13,-0.07) | -15.15(-21.65,-7.81) |
| Czechia | 879(611,1174) | 35.09(32.79,37.52) |  | 795(567,1097) | 36.48(33.87,39.25) |  | 0.14(0.12,0.16) | -9.55(-17.65,-0.53) |
| North Korea | 5106(3582,6861) | 87.83(85.41,90.31) |  | 5864(4105,7917) | 89.25(86.97,91.57) |  | 0.06(0.04,0.09) | 14.84(4.29,26.65) |
| Sao Tome and Principe | 97(66,133) | 352.22(282.00,438.20) |  | 192(133,260) | 326.90(281.50,378.40) |  | -0.52(-0.82,-0.23) | 97.57(77.70,119.40) |
| DR Congo | 125281(91436,166377) | 1356.00(1348.20,1363.83) |  | 262469(182173,354313) | 1141.46(1136.95,1145.98) |  | -1.94(-2.29,-1.59) | 109.50(82.46,131.69) |
| Timor-Leste | 278(185,390) | 136.10(120.23,153.89) |  | 508(332,715) | 132.63(120.88,145.37) |  | 0.13(0.06,0.21) | 82.68(67.39,99.08) |
| Sri Lanka | 2032(1412,2769) | 42.37(40.53,44.28) |  | 2172(1539,2857) | 39.13(37.50,40.81) |  | -1.27(-1.81,-0.72) | 6.86(-2.63,17.16) |
| Dominican Republic | 7150(4959,9733) | 345.94(337.64,354.43) |  | 10672(7425,14466) | 358.29(351.51,365.18) |  | 0.87(0.67,1.08) | 49.27(34.75,66.20) |
| Uruguay | 2242(1571,3026) | 295.39(283.28,307.90) |  | 2403(1683,3215) | 293.56(281.92,305.57) |  | 0.22(0.00,0.43) | 7.17(-1.24,15.34) |
| Ethiopia | 119843(75803,167887) | 962.45(956.80,968.14) |  | 151574(100413,207856) | 513.37(510.69,516.06) |  | -2.37(-2.75,-1.98) | 26.48(17.69,38.48) |
| Nepal | 24861(17148,33397) | 509.56(503.10,516.09) |  | 41967(28881,56087) | 439.60(435.36,443.87) |  | -0.34(-0.41,-0.26) | 68.81(55.25,87.09) |
| Germany | 7012(4896,9664) | 35.65(34.81,36.51) |  | 5673(4013,7694) | 34.53(33.63,35.46) |  | -0.23(-0.32,-0.13) | -19.10(-26.61,-10.86) |
| Nigeria | 124110(82433,172047) | 567.41(564.08,570.77) |  | 238393(159521,328712) | 391.72(390.08,393.36) |  | -0.39(-0.68,-0.09) | 92.08(84.99,99.01) |
| Somalia | 18034(12634,23873) | 1010.76(995.76,1025.97) |  | 42101(29152,57335) | 806.53(798.53,814.61) |  | 0.26(-0.39,0.91) | 133.45(111.34,160.42) |
| Federated States of Micronesia | 167(116,228) | 670.14(568.19,789.90) |  | 177(122,241) | 649.05(555.83,754.35) |  | 0.61(0.39,0.84) | 5.89(-5.38,15.25) |
| Brazil | 136858(91381,187255) | 334.46(332.66,336.27) |  | 290599(210019,370712) | 502.99(501.16,504.83) |  | -0.62(-2.08,0.87) | 112.34(85.19,147.33) |
| France | 8219(5729,11071) | 56.73(55.50,57.97) |  | 7513(5238,10340) | 55.08(53.83,56.35) |  | 0.10(-0.02,0.22) | -8.59(-15.40,-1.39) |
| Gabon | 3139(2251,4124) | 1362.23(1312.06,1414.23) |  | 5271(3644,7178) | 1011.62(984.01,1039.90) |  | -3.06(-3.72,-2.39) | 67.91(50.20,84.73) |
| Georgia | 1181(810,1595) | 83.88(79.14,88.84) |  | 592(407,803) | 79.63(73.23,86.47) |  | -1.84(-2.32,-1.36) | -49.82(-54.16,-43.87) |
| Luxembourg | 41(28,56) | 42.42(30.38,58.24) |  | 60(42,82) | 39.88(30.22,52.02) |  | -0.19(-0.26,-0.13) | 43.64(29.60,55.25) |
| Greenland | 8(5,11) | 50.07(20.72,105.93) |  | 6(5,9) | 49.42(18.72,108.15) |  | -0.05(-0.09,-0.02) | -19.23(-27.52,-10.66) |
| Grenada | 91(62,125) | 431.39(344.65,538.11) |  | 121(83,165) | 461.78(382.75,553.20) |  | 0.98(0.78,1.18) | 33.26(20.86,47.16) |
| Guam | 184(128,249) | 499.23(428.61,580.60) |  | 174(122,235) | 482.69(413.43,560.61) |  | -0.05(-0.09,-0.02) | -5.59(-13.87,3.01) |
| Jordan | 1320(881,1861) | 145.84(137.51,154.64) |  | 4584(3128,6176) | 143.94(139.76,148.22) |  | -0.01(-0.04,0.03) | 247.28(212.24,283.59) |
| Greece | 945(782,1105) | 37.57(35.21,40.05) |  | 851(590,1164) | 42.55(39.66,45.61) |  | 1.39(0.96,1.81) | -9.90(-31.58,14.98) |
| Hungary | 1749(1240,2329) | 71.23(67.90,74.69) |  | 1473(1047,1917) | 73.29(69.47,77.28) |  | 0.68(0.34,1.02) | -15.78(-27.67,-2.05) |
| Papua New Guinea | 7329(5184,9910) | 695.26(678.94,711.95) |  | 17670(12239,24078) | 645.80(636.24,655.49) |  | -0.97(-1.35,-0.60) | 141.08(114.35,170.77) |
| Samoa | 58(40,78) | 147.67(110.38,195.77) |  | 74(51,97) | 146.92(114.92,185.86) |  | -0.48(-0.64,-0.31) | 28.23(16.73,42.57) |
| Ireland | 360(251,482) | 40.54(36.45,45.00) |  | 445(318,602) | 40.39(36.66,44.41) |  | -0.02(-0.11,0.07) | 23.42(11.42,36.51) |
| Afghanistan | 2215(1509,3043) | 99.11(94.63,103.80) |  | 7854(5285,10794) | 102.63(100.24,105.06) |  | 0.23(0.16,0.30) | 254.56(218.06,294.43) |
| Iran | 9628(6409,13527) | 73.46(71.93,75.02) |  | 16979(11667,23530) | 75.05(73.90,76.21) |  | -0.01(-0.10,0.08) | 76.36(52.53,98.59) |
| Mauritania | 4182(2917,5587) | 819.61(794.05,845.95) |  | 7473(5101,10307) | 644.32(629.34,659.62) |  | -0.37(-0.55,-0.20) | 78.72(59.57,99.52) |
| Pakistan | 100075(66169,141400) | 409.47(406.87,412.10) |  | 229838(152722,324186) | 363.66(362.16,365.17) |  | -0.28(-0.44,-0.13) | 129.66(116.72,144.03) |
| Jamaica | 4664(3441,5947) | 692.69(672.24,713.75) |  | 3451(2385,4649) | 436.42(421.93,451.29) |  | -0.23(-0.63,0.16) | -26.01(-43.30,-6.75) |
| Japan | 33748(23698,45625) | 109.36(108.18,110.54) |  | 23890(16786,32254) | 102.00(100.68,103.34) |  | -0.27(-0.33,-0.20) | -29.21(-32.42,-26.03) |
| Bahrain | 217(147,299) | 172.21(148.93,199.32) |  | 559(389,761) | 171.67(157.71,186.58) |  | 0.02(-0.02,0.05) | 157.21(130.39,192.92) |
| Belgium | 1009(691,1374) | 41.29(38.77,43.94) |  | 955(655,1294) | 39.89(37.38,42.53) |  | -0.10(-0.17,-0.04) | -5.36(-13.09,4.07) |
| Bhutan | 1384(938,1880) | 872.65(825.00,923.00) |  | 1686(1174,2297) | 781.37(744.39,819.89) |  | -0.69(-0.82,-0.56) | 21.80(9.37,35.49) |
| Cambodia | 1251(864,1722) | 46.40(43.79,49.15) |  | 2063(1414,2779) | 44.31(42.41,46.28) |  | -2.42(-3.16,-1.67) | 64.87(48.32,82.59) |
| Denmark | 314(219,426) | 24.39(21.76,27.26) |  | 299(209,406) | 24.03(21.36,26.94) |  | -1.24(-1.68,-0.79) | -4.91(-12.62,3.29) |
| Eswatini | 3632(2430,4771) | 1634.56(1579.15,1691.89) |  | 3198(2181,4293) | 946.19(913.09,980.41) |  | -1.69(-2.12,-1.25) | -11.95(-34.50,14.44) |
| Lesotho | 4772(3273,6341) | 1194.80(1160.54,1229.91) |  | 5202(3567,7145) | 953.14(926.80,980.16) |  | -0.81(-0.99,-0.63) | 9.00(-6.55,22.67) |
| Morocco | 28772(19343,41084) | 417.33(412.38,422.33) |  | 38472(26151,53803) | 396.75(392.79,400.73) |  | 0.40(-0.17,0.97) | 33.71(18.16,51.81) |
| Norway | 467(324,641) | 44.29(40.36,48.51) |  | 496(345,680) | 41.85(38.22,45.75) |  | -0.16(-0.24,-0.09) | 6.16(2.38,10.42) |
| Saudi Arabia | 1682(1154,2243) | 48.83(46.42,51.37) |  | 5075(3540,6760) | 50.26(48.86,51.68) |  | -0.15(-0.26,-0.04) | 201.80(166.60,245.22) |
| Spain | 4259(2945,5820) | 43.62(42.32,44.95) |  | 3781(2615,5160) | 41.82(40.45,43.22) |  | -0.19(-0.29,-0.10) | -11.22(-21.88,0.26) |
| Sweden | 863(588,1202) | 43.04(40.20,46.03) |  | 868(601,1216) | 40.71(38.02,43.55) |  | -0.18(-0.24,-0.12) | 0.61(-6.01,7.40) |
| Thailand | 16107(10961,21939) | 94.36(92.89,95.85) |  | 13532(9535,18120) | 89.26(87.74,90.80) |  | -4.79(-6.17,-3.39) | -15.99(-23.97,-4.85) |
| Netherlands | 1658(1152,2307) | 41.63(39.64,43.69) |  | 1422(977,1938) | 39.69(37.65,41.82) |  | -0.15(-0.22,-0.08) | -14.24(-21.85,-6.04) |
| Tonga | 88(61,120) | 372.30(295.94,464.57) |  | 95(67,130) | 366.40(295.89,449.66) |  | -0.21(-0.37,-0.04) | 8.15(-0.47,16.73) |
| Kyrgyzstan | 274(188,370) | 24.65(21.71,28.00) |  | 414(285,562) | 23.50(21.28,25.89) |  | -2.47(-3.21,-1.72) | 50.90(35.60,64.74) |
| Laos | 1029(697,1413) | 98.26(92.18,104.70) |  | 2014(1381,2742) | 96.89(92.68,101.27) |  | 0.01(-0.02,0.03) | 95.64(78.88,114.83) |
| Lebanon | 1022(702,1404) | 130.90(122.89,139.34) |  | 1925(1350,2612) | 131.51(125.60,137.65) |  | 0.05(0.02,0.08) | 88.38(68.08,113.43) |
| Malaysia | 2119(1451,2873) | 44.50(42.59,46.49) |  | 3614(2512,4851) | 41.80(40.45,43.19) |  | -1.48(-1.89,-1.07) | 70.59(56.53,89.69) |
| Mongolia | 1010(668,1447) | 176.17(164.89,188.19) |  | 1649(1101,2240) | 205.63(195.77,215.88) |  | 0.89(0.53,1.25) | 63.25(35.07,104.47) |
| Montenegro | 55(38,75) | 35.15(26.49,45.90) |  | 51(37,71) | 36.07(26.75,47.74) |  | 0.09(0.07,0.12) | -8.35(-16.42,0.52) |
| New Zealand | 835(569,1144) | 91.51(85.40,97.96) |  | 996(686,1369) | 83.66(78.53,89.06) |  | -0.30(-0.36,-0.24) | 19.33(11.85,26.62) |
| North Macedonia | 174(119,236) | 34.00(29.14,39.48) |  | 181(125,249) | 35.01(30.01,40.69) |  | 0.11(0.09,0.13) | 3.94(-5.41,14.05) |
| Northern Mariana Islands | 81(56,109) | 546.99(430.49,694.82) |  | 57(40,77) | 520.63(392.04,680.06) |  | -0.13(-0.16,-0.11) | -29.45(-36.13,-20.17) |
| Palestine | 482(319,668) | 101.13(91.76,111.36) |  | 1392(947,1919) | 101.34(95.97,106.97) |  | 0.04(0.03,0.06) | 188.78(159.90,222.54) |
| Algeria | 6724(4538,9340) | 107.28(104.59,110.03) |  | 11887(8288,15896) | 107.11(105.18,109.06) |  | -1.15(-1.87,-0.44) | 76.78(53.46,101.06) |
| Bangladesh | 124482(85579,165413) | 466.33(463.62,469.06) |  | 183225(128062,246956) | 386.25(384.48,388.03) |  | -1.67(-1.98,-1.36) | 47.19(33.86,67.76) |
| China | 324579(221229,445920) | 98.73(98.38,99.08) |  | 340165(238060,459718) | 109.91(109.53,110.29) |  | -0.62(-1.11,-0.13) | 4.80(-8.16,16.50) |
| Bolivia | 8604(5793,11728) | 514.14(503.09,525.41) |  | 15804(10852,21566) | 487.90(480.30,495.60) |  | -1.41(-1.88,-0.94) | 83.68(66.47,102.24) |
| Portugal | 1032(715,1393) | 40.79(38.34,43.37) |  | 879(619,1174) | 40.67(37.96,43.54) |  | 0.03(-0.02,0.08) | -14.85(-23.21,-5.35) |
| Andorra | 6(4,9) | 41.84(15.72,92.96) |  | 7(5,10) | 40.90(16.27,88.46) |  | -0.10(-0.17,-0.04) | 17.31(5.05,32.70) |
| Monaco | 3(2,4) | 40.27(6.77,149.86) |  | 3(2,4) | 39.45(6.67,131.43) |  | -0.08(-0.13,-0.03) | -1.41(-8.15,6.29) |
| Puerto Rico | 2258(1579,3057) | 233.64(224.08,243.50) |  | 1700(1208,2275) | 234.28(223.22,245.76) |  | 0.02(-0.01,0.05) | -24.72(-30.56,-18.61) |
| Albania | 305(208,419) | 35.01(31.07,39.39) |  | 217(150,305) | 35.26(30.71,40.32) |  | 0.04(0.03,0.06) | -28.68(-35.60,-20.17) |
| Angola | 30499(21391,41558) | 1243.30(1228.88,1257.87) |  | 91185(63245,123893) | 1107.69(1100.31,1115.12) |  | -0.33(-0.42,-0.24) | 198.98(172.06,227.14) |
| Armenia | 171(118,233) | 18.98(16.17,22.24) |  | 126(88,172) | 17.44(14.46,20.93) |  | -0.61(-0.73,-0.48) | -26.24(-33.38,-18.36) |
| Austria | 821(562,1139) | 40.94(38.17,43.87) |  | 750(523,1027) | 39.51(36.69,42.51) |  | -0.09(-0.16,-0.02) | -8.61(-17.84,-0.37) |
| Azerbaijan | 912(623,1242) | 44.90(41.89,48.11) |  | 1097(765,1494) | 40.39(38.00,42.91) |  | -0.44(-0.50,-0.38) | 20.21(5.99,36.02) |
| Belarus | 683(480,934) | 26.57(24.60,28.65) |  | 514(355,686) | 25.28(23.06,27.68) |  | -1.77(-2.29,-1.25) | -24.85(-33.15,-16.12) |
| Benin | 4708(3227,6478) | 396.96(385.23,409.03) |  | 12375(8462,16598) | 353.00(346.57,359.53) |  | -1.62(-2.10,-1.13) | 162.85(137.54,201.55) |
| Botswana | 6782(5240,8635) | 1834.39(1788.87,1881.06) |  | 6299(4434,8443) | 911.02(888.60,933.92) |  | -3.05(-4.29,-1.79) | -7.13(-29.01,17.14) |
| Bulgaria | 415(290,569) | 20.33(18.41,22.40) |  | 275(195,378) | 20.11(17.71,22.78) |  | -0.20(-0.28,-0.13) | -33.77(-39.79,-27.20) |
| Burundi | 5430(3803,7361) | 400.44(389.44,411.73) |  | 12160(8493,16346) | 360.63(353.99,367.40) |  | -2.56(-3.60,-1.51) | 123.95(106.57,147.15) |
| Cabo Verde | 270(186,368) | 311.93(273.40,355.72) |  | 460(319,622) | 297.42(270.72,326.32) |  | -1.18(-1.60,-0.75) | 70.64(53.87,87.92) |
| Cameroon | 39827(28304,51863) | 1452.56(1437.77,1467.49) |  | 65959(45652,89526) | 773.33(767.26,779.44) |  | -1.72(-2.10,-1.35) | 65.61(35.13,100.87) |
| Chad | 11973(8405,16040) | 832.29(816.92,847.92) |  | 30263(20549,41532) | 705.99(697.63,714.44) |  | -0.36(-0.57,-0.16) | 152.76(126.13,185.41) |
| Chile | 5881(4099,8043) | 155.12(151.14,159.18) |  | 7135(5010,9619) | 153.01(149.46,156.62) |  | -1.82(-2.61,-1.01) | 21.32(10.50,34.69) |
| Colombia | 16376(11193,22305) | 176.01(173.25,178.80) |  | 22210(15438,30008) | 166.94(164.75,169.16) |  | -0.13(-0.24,-0.01) | 35.63(22.16,48.32) |
| Costa Rica | 1140(786,1525) | 137.24(129.21,145.75) |  | 1710(1194,2312) | 131.55(125.38,137.97) |  | -0.01(-0.04,0.03) | 49.99(38.47,64.48) |
| Croatia | 419(293,568) | 35.13(31.84,38.69) |  | 307(215,424) | 35.29(31.38,39.58) |  | 0.01(-0.01,0.04) | -26.83(-33.38,-20.75) |
| Cuba | 5592(3813,7569) | 173.94(169.34,178.65) |  | 4245(3114,5490) | 177.51(172.15,183.00) |  | -1.40(-2.15,-0.65) | -24.10(-33.90,-13.34) |
| Cyprus | 80(55,110) | 40.41(32.06,50.32) |  | 134(93,188) | 39.77(33.07,47.65) |  | -0.06(-0.12,0.01) | 66.75(51.00,85.02) |
| Ivory Coast | 24120(17442,32478) | 784.30(773.89,794.85) |  | 39934(27412,55866) | 561.70(556.08,567.37) |  | -0.82(-1.03,-0.62) | 65.56(40.13,90.44) |
| Djibouti | 905(624,1255) | 848.46(791.30,909.57) |  | 2675(1881,3601) | 822.20(791.26,854.13) |  | -0.02(-0.19,0.15) | 195.68(156.35,233.36) |
| Ecuador | 10719(7328,14610) | 383.95(376.52,391.53) |  | 18273(12623,24845) | 375.16(369.73,380.66) |  | -0.01(-0.04,0.01) | 70.47(56.01,87.26) |
| El Salvador | 1029(707,1396) | 74.34(69.70,79.25) |  | 1280(881,1714) | 70.27(66.46,74.26) |  | -1.19(-1.54,-0.84) | 24.40(12.56,41.02) |
| Equatorial Guinea | 1636(1141,2191) | 1568.42(1490.96,1649.39) |  | 5502(3784,7399) | 1405.54(1367.73,1444.33) |  | 0.24(0.06,0.42) | 236.35(203.70,269.21) |
| Estonia | 153(107,205) | 40.46(34.27,47.48) |  | 96(65,133) | 36.07(29.04,44.42) |  | -0.30(-0.38,-0.22) | -37.22(-43.89,-31.78) |
| Fiji | 2344(1598,3211) | 1113.41(1068.16,1160.35) |  | 2013(1402,2701) | 872.72(834.97,911.79) |  | -0.44(-0.59,-0.29) | -14.10(-29.66,1.10) |
| Finland | 510(359,688) | 41.54(37.97,45.38) |  | 439(303,592) | 39.81(36.15,43.77) |  | -0.13(-0.21,-0.05) | -13.96(-19.76,-8.26) |
| Ghana | 24733(17579,32963) | 646.80(638.47,655.23) |  | 58260(41178,80507) | 601.15(596.21,606.12) |  | -0.28(-0.58,0.02) | 135.56(114.09,156.88) |
| Guatemala | 2469(1701,3385) | 126.69(121.57,132.01) |  | 5998(4190,8099) | 130.09(126.77,133.49) |  | -0.06(-0.29,0.16) | 142.97(118.54,164.42) |
| Guinea | 10396(7349,13908) | 717.35(703.38,731.57) |  | 22121(15344,30587) | 613.80(605.48,622.22) |  | -0.60(-0.87,-0.33) | 112.78(89.86,135.11) |
| Guinea-Bissau | 1424(1026,1906) | 567.40(537.21,599.18) |  | 2621(1820,3612) | 466.14(447.97,485.01) |  | -0.51(-0.63,-0.40) | 84.08(67.78,105.35) |
| Guyana | 194(135,260) | 89.18(76.64,103.65) |  | 205(142,272) | 97.73(84.69,112.34) |  | -0.08(-0.28,0.11) | 5.65(-3.96,16.37) |
| Haiti | 10837(7445,14882) | 638.14(625.91,650.60) |  | 20154(14184,26842) | 551.01(543.39,558.72) |  | -0.42(-0.68,-0.15) | 85.97(55.80,111.61) |
| Honduras | 992(687,1327) | 90.27(84.50,96.40) |  | 2441(1705,3266) | 83.54(80.21,86.99) |  | -1.91(-2.64,-1.17) | 146.10(123.83,171.27) |
| Iceland | 28(19,37) | 41.67(27.57,61.03) |  | 31(22,42) | 39.43(26.75,56.34) |  | -0.17(-0.22,-0.11) | 11.87(4.69,22.00) |
| India | 730995(488118,984974) | 345.04(344.24,345.85) |  | 974483(665483,1316210) | 251.22(250.72,251.72) |  | -3.37(-4.25,-2.48) | 33.31(29.55,37.32) |
| Indonesia | 193724(128842,269008) | 374.88(373.17,376.59) |  | 267953(185079,368884) | 357.89(356.54,359.25) |  | 1.04(0.70,1.39) | 38.32(29.48,48.61) |
| Iraq | 4404(3019,6081) | 102.38(99.23,105.62) |  | 11017(7595,14999) | 101.19(99.29,103.12) |  | -0.04(-0.05,-0.03) | 150.17(130.23,173.38) |
| Italy | 6335(4379,8647) | 44.18(43.10,45.28) |  | 4754(3340,6540) | 42.14(40.92,43.39) |  | -0.15(-0.21,-0.08) | -24.96(-29.17,-20.09) |
| Kazakhstan | 1712(1180,2340) | 39.92(38.03,41.90) |  | 1862(1310,2498) | 40.15(38.33,42.04) |  | -0.50(-0.80,-0.21) | 8.79(-0.92,19.77) |
| Kenya | 74234(49118,101654) | 1286.25(1276.35,1296.22) |  | 85282(58006,116009) | 604.83(600.66,609.03) |  | -4.17(-5.00,-3.33) | 14.88(7.30,22.22) |
| Kiribati | 145(101,195) | 720.54(605.29,855.52) |  | 225(160,306) | 680.12(593.67,776.95) |  | 0.24(0.08,0.41) | 55.20(42.23,72.33) |
| South Korea | 16362(11323,21974) | 124.35(122.43,126.30) |  | 13556(9446,18112) | 122.14(120.03,124.28) |  | 0.17(0.08,0.27) | -17.15(-26.00,-6.22) |
| Latvia | 262(182,354) | 40.74(35.94,46.02) |  | 137(96,187) | 36.94(30.83,44.03) |  | -0.33(-0.37,-0.28) | -47.86(-53.53,-42.83) |
| Liberia | 8237(5658,11096) | 1324.93(1295.18,1355.36) |  | 16708(11418,22306) | 1106.95(1089.90,1124.24) |  | 0.47(0.15,0.79) | 102.84(81.40,127.11) |
| Lithuania | 329(228,455) | 35.53(31.79,39.59) |  | 188(132,254) | 33.61(28.90,38.96) |  | -0.78(-1.00,-0.56) | -43.06(-47.89,-37.99) |
| Madagascar | 45691(31290,60062) | 1501.96(1487.60,1516.45) |  | 84630(59687,114815) | 1072.92(1065.48,1080.40) |  | -1.49(-1.66,-1.33) | 85.22(47.32,121.39) |
| Malawi | 21313(17101,26219) | 851.22(839.22,863.40) |  | 21382(17448,25555) | 398.35(392.74,404.05) |  | -1.56(-1.95,-1.17) | 0.32(-17.21,24.16) |
| Maldives | 62(42,85) | 118.89(89.37,157.21) |  | 100(70,133) | 87.77(71.15,107.37) |  | -0.95(-1.05,-0.85) | 60.95(38.15,84.88) |
| Mali | 19826(14054,26632) | 952.71(939.12,966.48) |  | 47754(32139,65232) | 786.62(779.22,794.09) |  | 0.79(0.42,1.18) | 140.87(108.39,171.58) |
| Malta | 32(23,43) | 35.06(23.94,49.82) |  | 31(22,43) | 34.90(23.53,50.51) |  | -0.09(-0.14,-0.03) | -2.95(-10.22,5.78) |
| Mauritius | 539(367,738) | 169.78(155.59,185.12) |  | 479(330,644) | 155.00(141.39,169.61) |  | 0.85(0.54,1.17) | -11.10(-19.59,-1.19) |
| Moldova | 923(635,1238) | 81.19(76.00,86.65) |  | 562(397,743) | 67.17(61.50,73.27) |  | 0.43(0.05,0.81) | -39.08(-46.23,-31.06) |
| Mozambique | 70275(53365,91150) | 2002.83(1987.66,2018.11) |  | 98611(66766,134391) | 1167.37(1159.74,1175.04) |  | -2.64(-3.00,-2.29) | 40.32(9.79,77.20) |
| Namibia | 3531(2424,4824) | 966.63(933.36,1001.01) |  | 6111(4236,8557) | 875.63(853.58,898.16) |  | -1.83(-2.66,-1.00) | 73.05(57.13,91.52) |
| Nauru | 19(13,27) | 739.07(440.98,1202.70) |  | 22(14,30) | 722.33(447.81,1125.47) |  | 0.53(0.36,0.70) | 13.11(2.81,22.60) |
| Nicaragua | 990(681,1354) | 102.52(95.91,109.57) |  | 1785(1242,2388) | 96.10(91.68,100.70) |  | -3.10(-4.29,-1.89) | 80.31(60.90,96.56) |
| Niue | 3(2,3) | 507.36(85.56,1681.47) |  | 2(1,3) | 488.28(51.35,1932.36) |  | -0.09(-0.12,-0.07) | -27.91(-32.79,-22.83) |
| Palau | 13(9,18) | 303.98(160.06,543.16) |  | 10(7,14) | 292.13(137.96,553.51) |  | 0.00(-0.03,0.03) | -21.12(-29.42,-11.07) |
| Panama | 1507(1034,2063) | 230.92(219.15,243.25) |  | 2486(1726,3334) | 229.27(220.33,238.49) |  | 0.91(0.61,1.20) | 64.98(48.15,82.85) |
| Paraguay | 6927(4770,9466) | 688.59(672.08,705.46) |  | 12825(9117,17165) | 657.03(645.65,668.57) |  | 1.26(0.91,1.61) | 85.14(67.32,106.40) |
| Peru | 18547(12597,25144) | 309.69(305.13,314.32) |  | 29042(20191,39060) | 298.15(294.72,301.60) |  | -2.96(-3.67,-2.24) | 56.58(44.19,74.79) |
| Poland | 3894(2711,5354) | 41.83(40.51,43.18) |  | 3219(2266,4442) | 37.86(36.51,39.24) |  | -0.31(-0.32,-0.29) | -17.33(-21.37,-13.62) |
| Rwanda | 12378(8691,17317) | 702.82(689.91,715.96) |  | 21784(15210,29310) | 584.25(576.36,592.24) |  | -1.31(-1.70,-0.91) | 75.99(56.52,97.45) |
| San Marino | 3(2,3) | 39.80(6.65,130.97) |  | 3(2,4) | 39.42(6.57,130.68) |  | -0.05(-0.10,0.01) | 3.57(-8.13,13.32) |
| Senegal | 5485(3833,7513) | 297.07(288.90,305.47) |  | 10549(7249,14050) | 254.75(249.78,259.80) |  | -2.35(-2.84,-1.85) | 92.31(71.93,116.15) |
| Serbia | 818(569,1136) | 35.19(32.82,37.69) |  | 701(496,961) | 35.48(32.87,38.24) |  | 0.02(0.00,0.04) | -14.30(-21.01,-3.55) |
| Seychelles | 20(14,27) | 102.59(61.54,166.56) |  | 20(14,27) | 86.48(52.94,133.75) |  | -0.57(-0.71,-0.43) | 0.20(-9.69,10.77) |
| Sierra Leone | 4334(3005,5997) | 398.12(385.86,410.77) |  | 8968(6116,12248) | 363.21(355.43,371.15) |  | -0.17(-0.30,-0.04) | 106.92(87.13,130.52) |
| Singapore | 1030(707,1394) | 106.50(100.05,113.30) |  | 1394(984,1871) | 101.79(96.10,107.79) |  | -0.18(-0.23,-0.13) | 35.31(17.87,53.61) |
| Slovenia | 176(123,236) | 35.42(30.38,41.08) |  | 142(98,195) | 35.41(29.63,42.08) |  | 0.01(-0.01,0.03) | -19.72(-26.99,-10.92) |
| South Africa | 238011(170732,310701) | 2252.71(2243.41,2262.03) |  | 161110(108967,221402) | 1033.37(1028.31,1038.44) |  | -2.40(-3.49,-1.30) | -32.31(-40.79,-24.02) |
| South Sudan | 18837(13134,25504) | 1313.46(1293.65,1333.59) |  | 30033(20333,41545) | 1219.23(1204.97,1233.63) |  | 0.26(-0.09,0.60) | 59.44(42.61,78.87) |
| Sudan | 16592(11183,22961) | 325.68(320.55,330.88) |  | 37520(25568,52337) | 312.32(309.10,315.56) |  | -0.12(-0.43,0.19) | 126.13(99.73,148.49) |
| Suriname | 84(58,111) | 82.60(65.52,103.22) |  | 125(90,163) | 86.30(71.82,102.87) |  | 0.07(-0.04,0.19) | 48.13(33.60,65.22) |
| Tajikistan | 500(337,684) | 37.82(34.35,41.67) |  | 925(641,1247) | 35.00(32.76,37.37) |  | -1.28(-1.67,-0.89) | 85.13(65.82,104.62) |
| Congo (Brazzaville) | 5902(4040,8125) | 983.82(957.54,1010.77) |  | 12982(8926,17304) | 880.51(865.30,895.94) |  | -0.28(-0.39,-0.17) | 119.95(101.26,144.01) |
| The Gambia | 1969(1371,2715) | 781.15(744.86,819.30) |  | 4663(3179,6618) | 692.54(672.01,713.68) |  | -0.77(-0.95,-0.60) | 136.79(116.65,159.06) |
| Marshall Islands | 68(48,95) | 640.95(490.74,840.47) |  | 96(66,128) | 633.55(512.49,776.33) |  | 0.56(0.26,0.87) | 40.33(27.87,54.93) |
| Niger | 3206(2208,4343) | 172.70(166.53,179.09) |  | 7374(5129,10012) | 127.74(124.64,130.92) |  | -2.20(-2.60,-1.79) | 130.01(107.44,155.71) |
| Philippines | 42178(28238,57804) | 250.55(248.09,253.02) |  | 79665(53670,109769) | 260.25(258.44,262.08) |  | 1.11(0.80,1.42) | 88.88(80.06,100.03) |
| Myanmar | 18208(12104,25513) | 158.53(156.17,160.92) |  | 23839(16133,32137) | 154.97(153.01,156.96) |  | 0.91(0.66,1.15) | 30.92(18.29,46.24) |
| Trinidad and Tobago | 477(332,648) | 148.09(134.95,162.32) |  | 488(347,652) | 149.21(136.14,163.26) |  | -1.13(-1.51,-0.75) | 2.44(-8.21,15.42) |
| Tunisia | 2213(1483,3046) | 101.52(97.20,106.02) |  | 3055(2168,4126) | 102.82(99.18,106.57) |  | 0.07(0.06,0.08) | 38.05(22.50,57.88) |
| Turkey | 23018(15789,32282) | 153.31(151.29,155.36) |  | 32480(22569,44490) | 152.45(150.79,154.12) |  | 0.03(0.00,0.05) | 41.11(25.87,58.01) |
| Uganda | 47973(33554,64507) | 1116.68(1105.99,1127.46) |  | 133753(92202,183226) | 1148.11(1141.66,1154.60) |  | 1.20(0.68,1.72) | 178.81(149.34,213.90) |
| Uzbekistan | 2473(1677,3325) | 46.34(44.42,48.34) |  | 3616(2482,4971) | 40.44(39.13,41.79) |  | -0.58(-0.63,-0.53) | 46.25(31.02,63.81) |
| Vanuatu | 267(188,364) | 700.78(616.63,795.73) |  | 578(400,795) | 698.60(642.09,759.23) |  | 0.38(0.23,0.54) | 115.95(95.00,138.63) |
| Yemen | 2286(1563,3243) | 79.34(76.00,82.82) |  | 6974(4709,9684) | 81.03(79.11,83.01) |  | 0.08(0.05,0.11) | 205.14(183.61,235.10) |
| Zambia | 28886(22717,36538) | 1373.55(1356.54,1390.76) |  | 58299(40593,78046) | 1075.54(1066.49,1084.68) |  | -1.33(-1.61,-1.06) | 101.83(54.83,156.28) |
| Zimbabwe | 29748(19293,40719) | 1097.21(1084.05,1110.54) |  | 30428(21358,41341) | 703.51(695.46,711.65) |  | -1.80(-2.37,-1.24) | 2.28(-24.73,29.82) |
| Romania | 4095(2881,5513) | 73.11(70.87,75.41) |  | 2517(1765,3388) | 65.45(62.85,68.14) |  | -0.20(-0.37,-0.02) | -38.54(-45.22,-30.85) |
| Russia | 17125(11674,23954) | 45.55(44.86,46.24) |  | 13846(9619,18719) | 42.67(41.93,43.41) |  | -0.24(-0.34,-0.14) | -19.15(-25.64,-13.36) |
| Saint Kitts and Nevis | 59(39,81) | 533.36(400.81,712.61) |  | 75(51,103) | 489.59(384.50,615.63) |  | -0.18(-0.23,-0.14) | 26.79(13.74,41.26) |
| Saint Lucia | 155(105,209) | 416.60(351.14,493.37) |  | 192(136,255) | 431.48(372.29,497.85) |  | 0.78(0.56,1.00) | 23.86(9.25,41.13) |
| Saint Vincent and the Grenadines | 66(45,91) | 229.62(175.06,300.41) |  | 64(45,87) | 233.47(179.80,298.63) |  | 0.08(0.07,0.09) | -2.46(-11.94,8.93) |
| Slovakia | 294(207,403) | 22.29(19.81,25.01) |  | 291(208,397) | 23.78(21.04,26.82) |  | 0.20(0.14,0.26) | -0.96(-11.96,9.78) |
| Viet Nam | 16070(11033,22221) | 85.04(83.68,86.43) |  | 20845(14597,28207) | 84.36(83.21,85.53) |  | -0.03(-0.07,0.01) | 29.72(17.72,46.61) |
| Solomon Islands | 675(466,922) | 819.97(755.79,889.26) |  | 1415(975,1926) | 787.59(746.65,830.37) |  | -0.12(-0.34,0.10) | 109.52(88.38,130.83) |
| Eritrea | 4582(3147,6531) | 546.84(530.56,563.55) |  | 8523(6004,11504) | 487.83(477.36,498.51) |  | -0.69(-0.91,-0.47) | 86.01(67.70,108.96) |
| Israel | 496(340,686) | 40.18(36.70,43.94) |  | 863(599,1164) | 39.13(36.56,41.84) |  | -0.05(-0.13,0.03) | 73.72(57.36,89.42) |
| Kuwait | 456(312,628) | 102.35(92.83,112.88) |  | 1407(997,1903) | 101.33(95.80,107.15) |  | 0.00(-0.01,0.01) | 208.27(168.64,259.66) |
| Libya | 993(672,1369) | 101.71(95.04,108.83) |  | 2019(1423,2712) | 103.18(98.72,107.79) |  | 0.09(0.08,0.11) | 103.34(79.04,135.57) |
| Qatar | 140(94,197) | 172.96(144.72,206.64) |  | 905(601,1263) | 168.37(156.82,180.66) |  | -0.08(-0.12,-0.04) | 545.80(484.78,618.16) |
| Oman | 122(84,162) | 34.53(28.49,41.72) |  | 352(247,477) | 34.48(30.91,38.38) |  | -1.66(-2.25,-1.08) | 188.59(160.33,223.38) |
| Switzerland | 536(369,723) | 30.60(28.04,33.35) |  | 549(382,749) | 29.34(26.89,31.98) |  | 0.03(-0.05,0.11) | 2.45(-6.54,12.15) |
| Syria | 3452(2319,4699) | 117.27(113.16,121.52) |  | 4132(2888,5513) | 109.06(105.50,112.72) |  | -0.26(-0.28,-0.23) | 19.69(4.36,33.96) |
| Taiwan (province of China) | 4835(3361,6400) | 85.11(82.70,87.59) |  | 4483(3149,5996) | 81.90(79.44,84.43) |  | -0.11(-0.23,0.01) | -7.27(-19.99,6.40) |
| Togo | 4112(2837,5691) | 445.98(431.88,460.52) |  | 8807(6092,11834) | 392.53(384.29,400.92) |  | -2.53(-3.22,-1.84) | 114.21(91.42,142.33) |
| Tokelau | 2(1,3) | 520.89(55.17,2267.41) |  | 2(1,2) | 494.43(39.80,2153.61) |  | -0.12(-0.14,-0.10) | -16.36(-22.80,-9.55) |
| Turkmenistan | 478(325,660) | 50.32(45.66,55.45) |  | 548(378,730) | 42.84(39.33,46.60) |  | -0.54(-0.63,-0.45) | 14.64(2.51,25.49) |
| Tuvalu | 9(6,14) | 381.67(178.31,730.39) |  | 11(7,15) | 356.09(174.21,660.17) |  | -0.19(-0.21,-0.16) | 12.70(4.19,21.68) |
| Ukraine | 5028(3477,6951) | 39.86(38.76,40.98) |  | 3655(2511,4964) | 38.17(36.89,39.49) |  | -0.45(-0.61,-0.29) | -27.32(-34.37,-18.52) |
| Comoros | 1266(852,1750) | 1082.40(1021.02,1147.19) |  | 1442(999,1977) | 707.46(671.12,745.38) |  | -2.62(-3.10,-2.13) | 13.88(-6.70,36.25) |
| United Arab Emirates | 138(96,188) | 37.96(31.57,45.82) |  | 588(418,812) | 38.17(34.81,41.81) |  | -1.88(-2.59,-1.17) | 324.77(253.76,420.12) |
| UK | 7715(5299,10665) | 53.99(52.78,55.21) |  | 7898(5432,10765) | 52.60(51.44,53.79) |  | 0.23(0.03,0.42) | 2.37(-0.69,5.57) |
| Mexico | 32202(21592,44238) | 138.94(137.37,140.53) |  | 42299(29424,57141) | 120.66(119.51,121.81) |  | -0.47(-0.90,-0.04) | 31.35(22.15,41.16) |
| Tanzania | 103623(80538,128940) | 1459.95(1450.59,1469.38) |  | 165248(116006,223811) | 1022.07(1017.00,1027.16) |  | -3.05(-3.64,-2.46) | 59.47(19.62,109.61) |
| USA | 96380(67013,132145) | 142.95(142.04,143.86) |  | 100377(70454,136430) | 134.05(133.22,134.88) |  | -0.41(-0.69,-0.12) | 4.15(1.75,6.92) |
| Virgin Islands | 66(47,88) | 236.36(182.61,301.40) |  | 40(29,52) | 243.48(173.07,334.39) |  | 0.16(0.14,0.19) | -39.77(-44.06,-34.45) |

ASR, age standardized rate; CI, confidence interval; EAPC, estimated annual percentage change; UI, uncertainty interval; SDI, socio-demographic index.

## Table S2 The prevalence of syphilis cases and rates among WCBA in 1990 and 2021 across 204 countries, and the trends from 1990 to 2021

| **Location** | **1990** | |  | **2021** | |  | **1990-2021** | |
| --- | --- | --- | --- | --- | --- | --- | --- | --- |
|  | **Cases number** | **ASR per 100,000** |  | **Cases number** | **ASR per 100,000** |  | **EAPC** | **Cases change(%)** |
|  | **No.  (95% UI)** | **No. (95% CI)** |  | **No.  (95% UI)** | **No. (95% CI)** |  | **No. (95% CI)** | **No. (95% UI)** |
| American Samoa | 247(173,337) | 1993.25(1744.29,2274.30) |  | 231(172,306) | 2013.59(1759.47,2295.70) |  | -0.04(-0.06,-0.01) | -6.71(-16.45,5.72) |
| Antigua and Barbuda | 128(89,174) | 756.89(629.47,906.52) |  | 176(123,239) | 733.35(628.66,851.40) |  | -0.18(-0.27,-0.09) | 38.04(25.48,52.81) |
| Egypt | 42416(28543,60721) | 322.47(319.35,325.62) |  | 78523(53169,110814) | 300.19(298.08,302.31) |  | -0.20(-0.24,-0.17) | 85.13(66.31,105.29) |
| Argentina | 94056(70143,121145) | 1174.05(1166.55,1181.60) |  | 149525(109479,202079) | 1258.08(1251.71,1264.48) |  | 1.20(0.68,1.73) | 58.97(40.81,77.35) |
| Australia | 13078(9141,17262) | 291.17(286.20,296.22) |  | 16721(11764,22316) | 276.65(272.44,280.91) |  | -0.20(-0.22,-0.17) | 27.86(14.42,42.24) |
| Barbados | 214(155,287) | 304.41(264.73,349.02) |  | 224(164,290) | 321.01(280.04,366.53) |  | -0.16(-0.25,-0.07) | 4.42(-6.70,15.37) |
| Belize | 231(161,318) | 532.82(462.58,613.39) |  | 614(434,834) | 503.11(463.78,545.10) |  | -0.52(-0.70,-0.35) | 165.37(140.66,195.13) |
| Bermuda | 151(105,204) | 855.81(723.18,1008.38) |  | 101(73,134) | 782.05(633.43,957.87) |  | -0.27(-0.30,-0.24) | -32.84(-40.02,-24.57) |
| Venezuela | 70122(48243,94925) | 1419.75(1408.99,1430.59) |  | 102578(73719,135530) | 1527.34(1517.93,1536.79) |  | 2.01(1.49,2.53) | 46.28(27.91,69.55) |
| Bosnia and Herzegovina | 1452(1009,1921) | 123.42(117.13,129.97) |  | 911(648,1212) | 127.20(118.94,135.94) |  | -0.01(-0.05,0.03) | -37.25(-44.90,-28.76) |
| Brunei | 223(156,297) | 316.50(274.89,364.25) |  | 400(291,517) | 318.78(288.16,351.96) |  | -0.10(-0.18,-0.03) | 79.37(61.77,101.45) |
| Burkina Faso | 31892(23676,42126) | 1513.65(1496.58,1530.90) |  | 82711(60697,112483) | 1471.33(1460.95,1481.78) |  | 0.70(0.36,1.04) | 159.35(128.19,190.78) |
| Canada | 6007(4134,8046) | 80.19(78.16,82.27) |  | 6338(4518,8374) | 76.49(74.60,78.42) |  | -0.65(-1.47,0.17) | 5.52(-2.78,16.05) |
| Central African Republic | 37194(27100,49623) | 5771.18(5710.52,5832.41) |  | 76737(56285,101731) | 5535.40(5495.27,5575.79) |  | -0.28(-0.57,0.02) | 106.31(86.11,127.18) |
| Dominica | 285(200,383) | 1651.02(1459.57,1863.13) |  | 289(212,382) | 1753.73(1557.34,1968.62) |  | 0.44(0.33,0.55) | 1.68(-9.60,15.51) |
| The Bahamas | 1007(709,1363) | 1338.56(1255.49,1426.44) |  | 1419(1027,1908) | 1332.81(1264.29,1404.12) |  | 0.43(0.23,0.63) | 41.00(24.93,57.90) |
| Cook Islands | 95(69,128) | 2030.91(1635.91,2501.97) |  | 92(69,119) | 2140.84(1723.59,2633.02) |  | 0.13(0.09,0.18) | -3.89(-11.82,6.43) |
| Czechia | 3029(2130,4082) | 119.91(115.63,124.31) |  | 2734(1939,3688) | 120.62(115.94,125.46) |  | -0.01(-0.04,0.01) | -9.76(-18.70,2.24) |
| North Korea | 18209(13032,24317) | 320.70(316.00,325.46) |  | 21475(15523,28763) | 324.78(320.43,329.17) |  | 0.00(-0.02,0.02) | 17.94(6.00,30.83) |
| Sao Tome and Principe | 356(257,477) | 1368.55(1222.67,1529.96) |  | 690(501,928) | 1212.76(1122.51,1309.10) |  | -0.98(-1.33,-0.63) | 93.95(74.31,114.22) |
| DR Congo | 507182(388954,658919) | 5866.45(5849.70,5883.24) |  | 976144(704720,1315541) | 4541.14(4531.86,4550.44) |  | -2.69(-3.15,-2.23) | 92.46(67.78,115.46) |
| Timor-Leste | 1087(756,1478) | 553.52(520.31,588.65) |  | 1865(1310,2533) | 519.69(495.41,544.98) |  | -0.06(-0.12,0.00) | 71.49(56.27,89.58) |
| Sri Lanka | 6525(4620,8910) | 138.44(135.07,141.88) |  | 6871(4891,9186) | 123.63(120.72,126.60) |  | -1.51(-2.10,-0.93) | 5.31(-4.68,16.46) |
| Dominican Republic | 25837(18376,35650) | 1321.06(1304.35,1337.98) |  | 39724(28897,53545) | 1347.29(1334.03,1360.65) |  | 0.88(0.62,1.15) | 53.74(37.69,74.16) |
| Uruguay | 8352(5903,11197) | 1111.58(1087.85,1135.70) |  | 8932(6414,11878) | 1083.56(1061.16,1106.33) |  | 0.10(-0.11,0.31) | 6.95(-1.64,19.51) |
| Ethiopia | 490057(351278,659800) | 4182.35(4170.19,4194.54) |  | 572773(417979,777663) | 2028.33(2022.88,2033.79) |  | -2.62(-3.00,-2.24) | 16.88(8.50,26.68) |
| Nepal | 89423(62664,118695) | 1933.57(1920.62,1946.60) |  | 150064(105786,208226) | 1616.73(1608.48,1625.01) |  | -0.60(-0.61,-0.58) | 67.81(51.14,86.74) |
| Germany | 22868(15943,31484) | 114.75(113.26,116.27) |  | 18787(13352,24923) | 111.05(109.44,112.67) |  | -0.26(-0.34,-0.17) | -17.85(-25.27,-9.01) |
| Nigeria | 449788(318612,612903) | 2202.03(2195.24,2208.85) |  | 839886(606494,1143698) | 1446.29(1443.08,1449.51) |  | -0.63(-0.88,-0.38) | 86.73(76.95,97.17) |
| Somalia | 77827(57342,102602) | 4575.30(4542.64,4608.18) |  | 160290(119039,210880) | 3295.59(3278.86,3312.41) |  | 0.07(-0.67,0.82) | 105.96(83.70,127.42) |
| Federated States of Micronesia | 693(497,923) | 3014.61(2784.72,3262.32) |  | 774(567,1037) | 2978.33(2769.87,3198.97) |  | 0.55(0.35,0.75) | 11.80(0.48,24.90) |
| Brazil | 515199(367207,690235) | 1302.22(1298.61,1305.85) |  | 1141825(922392,1424423) | 1949.27(1945.69,1952.86) |  | -0.60(-2.05,0.86) | 121.63(89.15,165.94) |
| France | 27620(19503,36257) | 189.76(187.53,192.02) |  | 26019(18018,35100) | 188.19(185.90,190.51) |  | 0.16(0.09,0.24) | -5.80(-13.89,3.33) |
| Gabon | 12177(9049,15884) | 5744.99(5637.15,5854.72) |  | 18934(13553,25999) | 3810.29(3755.26,3866.01) |  | -4.09(-4.90,-3.28) | 55.49(39.09,71.72) |
| Georgia | 4418(3121,6070) | 312.97(303.75,322.40) |  | 2434(1724,3263) | 312.78(300.28,325.72) |  | -1.99(-2.54,-1.43) | -44.90(-50.57,-37.89) |
| Luxembourg | 137(97,179) | 137.84(115.49,163.77) |  | 199(140,271) | 128.52(110.99,148.39) |  | -0.22(-0.26,-0.19) | 45.66(30.90,61.23) |
| Greenland | 35(24,48) | 237.25(162.82,336.71) |  | 32(23,42) | 254.00(173.40,360.77) |  | 0.16(0.03,0.28) | -8.03(-20.88,8.36) |
| Grenada | 349(243,478) | 1754.56(1569.65,1959.13) |  | 489(351,649) | 1888.52(1723.84,2065.42) |  | 1.14(0.88,1.39) | 39.99(26.59,57.10) |
| Guam | 694(497,950) | 1919.39(1776.71,2072.63) |  | 673(479,893) | 1884.02(1744.02,2032.59) |  | -0.05(-0.07,-0.04) | -2.98(-12.24,8.74) |
| Jordan | 4613(3157,6388) | 555.45(538.44,572.95) |  | 16515(11649,22333) | 535.31(527.10,543.62) |  | -0.14(-0.19,-0.09) | 258.00(219.02,297.80) |
| Greece | 3065(2716,3442) | 121.90(117.62,126.30) |  | 2909(2027,3856) | 140.56(135.35,145.94) |  | 1.47(1.05,1.88) | -5.11(-29.17,22.47) |
| Hungary | 6587(4632,8679) | 263.00(256.63,269.51) |  | 5880(4593,7246) | 280.61(273.24,288.14) |  | 1.00(0.62,1.37) | -10.74(-24.49,9.67) |
| Papua New Guinea | 31612(23406,42076) | 3231.55(3194.88,3268.61) |  | 78004(55656,105313) | 2945.66(2924.85,2966.60) |  | -1.29(-1.75,-0.83) | 146.75(118.23,176.52) |
| Samoa | 185(133,250) | 491.89(420.27,574.08) |  | 246(180,324) | 496.13(435.17,563.90) |  | -0.60(-0.82,-0.39) | 33.04(22.23,46.88) |
| Ireland | 1169(821,1537) | 133.43(125.86,141.35) |  | 1466(1042,1955) | 130.62(123.90,137.63) |  | -0.10(-0.15,-0.05) | 25.39(11.88,42.26) |
| Afghanistan | 8268(5977,11014) | 404.42(394.99,414.06) |  | 27867(19596,38345) | 389.09(384.28,393.95) |  | -0.11(-0.17,-0.05) | 237.04(201.40,273.51) |
| Iran | 34882(24400,48829) | 279.26(276.21,282.35) |  | 65856(45826,90852) | 280.76(278.58,282.96) |  | -0.10(-0.20,-0.01) | 88.80(68.41,110.89) |
| Mauritania | 18264(13268,24300) | 3825.68(3768.46,3883.69) |  | 30217(21309,41422) | 2782.08(2749.90,2814.61) |  | -0.61(-0.82,-0.39) | 65.45(47.77,84.10) |
| Pakistan | 334851(232256,468383) | 1444.86(1439.83,1449.90) |  | 792584(550915,1119171) | 1287.39(1284.53,1290.26) |  | -0.36(-0.53,-0.18) | 136.70(125.12,150.88) |
| Jamaica | 18840(16013,21752) | 3022.13(2977.45,3067.44) |  | 13553(9647,18027) | 1716.57(1687.70,1745.84) |  | -0.26(-0.77,0.25) | -28.06(-46.91,-9.02) |
| Japan | 121108(87575,162709) | 390.15(387.93,392.38) |  | 85620(61356,115241) | 355.09(352.65,357.54) |  | -0.35(-0.41,-0.29) | -29.30(-32.56,-25.80) |
| Bahrain | 807(545,1124) | 651.40(604.62,701.91) |  | 2161(1517,2912) | 658.45(630.94,686.89) |  | 0.05(0.02,0.07) | 167.68(134.26,211.78) |
| Belgium | 3308(2307,4505) | 133.96(129.42,138.63) |  | 3170(2251,4246) | 129.53(125.03,134.17) |  | -0.12(-0.16,-0.09) | -4.17(-12.42,5.56) |
| Bhutan | 5071(3582,6738) | 3521.37(3420.12,3625.43) |  | 6812(4776,9136) | 3192.29(3116.59,3269.53) |  | -0.89(-1.08,-0.69) | 34.34(18.89,53.04) |
| Cambodia | 4311(3041,6033) | 165.20(160.16,170.37) |  | 6799(4775,9238) | 146.47(143.00,150.02) |  | -3.03(-3.87,-2.18) | 57.69(44.56,74.05) |
| Denmark | 976(678,1313) | 75.34(70.67,80.25) |  | 928(648,1234) | 73.89(69.18,78.85) |  | -1.34(-1.81,-0.87) | -4.95(-13.86,5.40) |
| Eswatini | 15205(10596,19205) | 7513.45(7388.72,7640.16) |  | 12333(8742,16837) | 3779.18(3711.47,3848.03) |  | -2.34(-2.80,-1.87) | -18.89(-40.30,7.89) |
| Lesotho | 19565(14146,26185) | 5142.46(5069.52,5216.28) |  | 20283(14553,27162) | 3912.17(3857.21,3967.83) |  | -1.21(-1.40,-1.02) | 3.67(-12.74,20.57) |
| Morocco | 142426(95973,193799) | 2267.35(2255.25,2279.52) |  | 220675(149795,304977) | 2278.54(2269.04,2288.07) |  | 1.38(0.35,2.43) | 54.94(35.61,80.44) |
| Norway | 1588(1123,2176) | 150.39(143.07,158.00) |  | 1694(1208,2325) | 140.30(133.66,147.20) |  | -0.19(-0.26,-0.13) | 6.68(2.70,10.92) |
| Saudi Arabia | 5402(3716,7644) | 163.40(158.88,168.05) |  | 16606(11565,22811) | 160.52(158.05,163.01) |  | -0.45(-0.57,-0.34) | 207.42(173.97,250.75) |
| Spain | 13768(9544,18725) | 142.66(140.29,145.07) |  | 12815(9044,17160) | 136.90(134.45,139.39) |  | -0.24(-0.39,-0.09) | -6.92(-17.35,5.31) |
| Sweden | 3124(2209,4355) | 153.99(148.60,159.53) |  | 3178(2217,4345) | 145.11(140.07,150.31) |  | -0.20(-0.25,-0.15) | 1.73(-5.11,9.13) |
| Thailand | 56346(39224,74853) | 337.82(334.99,340.66) |  | 47380(33955,63076) | 305.32(302.54,308.13) |  | -5.55(-7.08,-3.99) | -15.91(-25.08,-5.63) |
| Netherlands | 5365(3758,7293) | 133.99(130.42,137.64) |  | 4680(3293,6207) | 128.99(125.30,132.76) |  | -0.12(-0.16,-0.09) | -12.76(-21.07,-2.25) |
| Tonga | 323(229,437) | 1456.61(1296.53,1632.64) |  | 358(265,489) | 1416.08(1271.82,1572.99) |  | -0.47(-0.70,-0.24) | 11.11(0.97,20.33) |
| Kyrgyzstan | 940(654,1302) | 86.19(80.54,92.25) |  | 1358(949,1853) | 77.07(73.01,81.31) |  | -2.92(-3.73,-2.10) | 44.55(28.76,59.56) |
| Laos | 3927(2757,5370) | 395.24(382.62,408.22) |  | 7462(5281,10304) | 364.63(356.35,373.08) |  | -0.31(-0.34,-0.27) | 90.02(72.41,111.16) |
| Lebanon | 3685(2559,4964) | 485.89(470.11,502.11) |  | 7002(4933,9623) | 462.35(451.42,473.50) |  | -0.18(-0.21,-0.15) | 90.04(67.59,117.51) |
| Malaysia | 6933(4869,9514) | 149.05(145.49,152.69) |  | 11584(8153,16054) | 134.15(131.71,136.63) |  | -1.81(-2.26,-1.35) | 67.10(51.12,86.51) |
| Mongolia | 4913(3344,6800) | 965.05(936.58,994.31) |  | 12675(8365,18532) | 1484.08(1458.22,1510.30) |  | 2.30(1.64,2.97) | 157.99(102.03,304.83) |
| Montenegro | 204(146,273) | 130.18(112.91,149.48) |  | 202(150,260) | 144.53(125.09,166.26) |  | 0.15(0.05,0.24) | -1.24(-11.40,10.31) |
| New Zealand | 2984(2098,4110) | 328.27(316.58,340.30) |  | 3571(2509,5042) | 295.44(285.80,305.34) |  | -0.36(-0.41,-0.30) | 19.67(10.49,29.62) |
| North Macedonia | 629(437,837) | 123.24(113.78,133.30) |  | 669(474,898) | 127.53(117.87,137.84) |  | 0.01(-0.03,0.05) | 6.48(-3.19,19.17) |
| Northern Mariana Islands | 305(212,405) | 2130.70(1889.45,2402.14) |  | 242(180,309) | 2192.41(1919.50,2495.16) |  | 0.01(-0.08,0.10) | -20.73(-30.18,-7.43) |
| Palestine | 1663(1132,2274) | 373.13(354.37,392.78) |  | 4807(3332,6644) | 362.39(351.99,373.06) |  | -0.10(-0.12,-0.09) | 189.11(157.56,224.46) |
| Algeria | 23403(16174,32073) | 396.71(391.38,402.12) |  | 42574(29878,56991) | 376.56(372.97,380.17) |  | -1.54(-2.35,-0.71) | 81.91(58.74,108.65) |
| Bangladesh | 419017(293427,555999) | 1685.92(1680.56,1691.29) |  | 629254(445983,854986) | 1348.25(1344.91,1351.60) |  | -2.10(-2.49,-1.70) | 50.17(34.06,69.03) |
| China | 1238959(887390,1674613) | 387.83(387.13,388.54) |  | 1363093(960904,1849089) | 425.11(424.38,425.85) |  | -0.68(-1.17,-0.19) | 10.02(-0.38,20.01) |
| Bolivia | 33631(23422,45278) | 2125.53(2102.37,2148.91) |  | 60194(42491,83190) | 1882.30(1867.23,1897.46) |  | -1.98(-2.57,-1.38) | 78.98(62.65,97.58) |
| Portugal | 3481(2437,4660) | 138.48(133.92,143.17) |  | 2987(2117,3906) | 134.18(129.27,139.23) |  | -0.12(-0.15,-0.10) | -14.20(-23.08,-4.33) |
| Andorra | 33(21,52) | 234.04(160.47,331.27) |  | 39(28,52) | 231.36(161.90,322.46) |  | 0.03(-0.15,0.22) | 17.56(-12.87,46.73) |
| Monaco | 14(9,21) | 239.20(126.76,420.24) |  | 13(10,18) | 207.73(110.41,358.99) |  | -0.37(-0.47,-0.28) | -4.30(-23.76,15.76) |
| Puerto Rico | 8015(5604,10840) | 837.98(819.71,856.56) |  | 5854(4206,7878) | 797.94(777.52,818.78) |  | -0.17(-0.20,-0.15) | -26.97(-33.99,-19.82) |
| Albania | 1041(715,1400) | 122.49(114.92,130.50) |  | 764(545,1010) | 124.12(115.45,133.30) |  | -0.03(-0.06,0.00) | -26.55(-34.23,-17.40) |
| Angola | 121286(88571,161333) | 5249.48(5218.94,5280.18) |  | 338436(243356,459628) | 4375.73(4360.61,4390.90) |  | -0.72(-0.82,-0.63) | 179.04(153.47,205.77) |
| Armenia | 539(380,732) | 60.35(55.21,65.91) |  | 401(279,541) | 54.07(48.78,59.84) |  | -0.73(-0.86,-0.59) | -25.73(-34.28,-15.95) |
| Austria | 2704(1884,3626) | 134.38(129.33,139.58) |  | 2531(1762,3365) | 129.22(124.17,134.45) |  | -0.14(-0.17,-0.10) | -6.39(-14.89,3.48) |
| Azerbaijan | 3127(2149,4241) | 157.51(151.75,163.48) |  | 3883(2769,5270) | 139.00(134.60,143.52) |  | -0.53(-0.58,-0.49) | 24.18(8.31,44.25) |
| Belarus | 2309(1608,3151) | 89.13(85.51,92.87) |  | 1735(1221,2297) | 82.79(78.80,86.96) |  | -2.09(-2.68,-1.50) | -24.88(-32.79,-14.44) |
| Benin | 18458(13363,24784) | 1619.79(1595.53,1644.40) |  | 43849(31372,58892) | 1317.38(1304.62,1330.24) |  | -2.41(-3.08,-1.73) | 137.56(112.14,167.88) |
| Botswana | 28264(25397,31328) | 8427.83(8325.15,8531.70) |  | 24308(17713,33176) | 3512.79(3468.61,3557.44) |  | -3.95(-5.41,-2.47) | -13.99(-35.86,12.11) |
| Bulgaria | 1391(969,1925) | 67.67(64.14,71.35) |  | 911(639,1226) | 64.67(60.38,69.22) |  | -0.39(-0.48,-0.30) | -34.51(-40.61,-27.64) |
| Burundi | 18532(13367,25068) | 1418.01(1396.86,1439.47) |  | 38576(27737,50878) | 1197.31(1184.90,1209.84) |  | -3.32(-4.60,-2.03) | 108.16(89.46,128.46) |
| Cabo Verde | 936(659,1302) | 1136.98(1060.47,1218.74) |  | 1622(1133,2227) | 1051.35(1000.48,1104.39) |  | -1.76(-2.31,-1.21) | 73.37(56.77,92.78) |
| Cameroon | 199930(158768,244029) | 8123.74(8086.74,8160.89) |  | 291931(209229,396776) | 3654.46(3640.82,3668.15) |  | -2.21(-2.68,-1.75) | 46.02(14.22,76.24) |
| Chad | 53725(38520,70589) | 4008.84(3973.90,4044.05) |  | 129825(94580,171677) | 3327.93(3308.96,3347.00) |  | -0.46(-0.77,-0.14) | 141.65(121.17,167.38) |
| Chile | 20189(13976,27525) | 543.42(535.87,551.05) |  | 24233(17064,32303) | 510.53(504.10,517.03) |  | -2.23(-3.11,-1.34) | 20.03(8.96,33.05) |
| Colombia | 57788(40292,80046) | 646.38(640.98,651.83) |  | 80108(56991,107789) | 602.65(598.48,606.85) |  | -0.28(-0.41,-0.15) | 38.62(25.51,53.47) |
| Costa Rica | 3899(2739,5274) | 483.36(467.92,499.27) |  | 5950(4201,7930) | 453.05(441.58,464.75) |  | -0.15(-0.18,-0.12) | 52.62(39.01,70.04) |
| Croatia | 1436(1004,1939) | 118.93(112.84,125.27) |  | 1042(737,1404) | 116.99(109.88,124.48) |  | -0.08(-0.10,-0.06) | -27.46(-34.42,-18.99) |
| Cuba | 19041(13109,25754) | 608.50(599.75,617.36) |  | 14405(10680,18913) | 589.77(580.08,599.59) |  | -1.80(-2.64,-0.96) | -24.35(-34.23,-12.68) |
| Cyprus | 281(198,375) | 141.03(125.00,158.59) |  | 494(348,659) | 143.27(130.38,157.28) |  | -0.09(-0.15,-0.03) | 76.06(58.57,98.66) |
| Ivory Coast | 106949(83027,135153) | 3748.19(3724.50,3772.02) |  | 167683(120008,226805) | 2457.01(2445.00,2469.07) |  | -1.19(-1.36,-1.03) | 56.79(29.31,81.32) |
| Djibouti | 3479(2530,4746) | 3492.68(3371.99,3617.39) |  | 10341(7488,13918) | 3173.78(3112.71,3235.84) |  | -0.34(-0.51,-0.18) | 197.28(167.17,234.30) |
| Ecuador | 38932(26902,54374) | 1469.48(1454.51,1484.59) |  | 65078(45579,89845) | 1348.73(1338.35,1359.16) |  | -0.30(-0.33,-0.27) | 67.16(51.26,85.95) |
| El Salvador | 3374(2359,4622) | 254.85(246.01,263.96) |  | 4158(2942,5682) | 230.57(223.58,237.73) |  | -1.58(-1.99,-1.17) | 23.22(11.43,37.36) |
| Equatorial Guinea | 6926(5065,9074) | 7126.81(6955.10,7302.13) |  | 20971(15263,27995) | 5666.52(5588.14,5745.89) |  | -0.21(-0.42,0.00) | 202.77(171.25,237.96) |
| Estonia | 535(383,707) | 140.38(128.70,152.88) |  | 340(232,453) | 123.95(110.80,138.37) |  | -0.42(-0.45,-0.40) | -36.56(-43.44,-29.36) |
| Fiji | 10562(7612,14471) | 5329.28(5226.53,5433.77) |  | 9385(6927,12439) | 4114.56(4031.63,4198.82) |  | -0.37(-0.64,-0.10) | -11.15(-31.03,5.73) |
| Finland | 1669(1167,2230) | 132.94(126.57,139.58) |  | 1449(1017,1939) | 128.83(122.24,135.70) |  | -0.09(-0.14,-0.05) | -13.19(-20.50,-5.11) |
| Ghana | 103088(74277,138648) | 2848.87(2830.88,2866.96) |  | 241837(174560,325916) | 2577.28(2566.88,2587.72) |  | -0.44(-0.80,-0.08) | 134.59(111.68,158.49) |
| Guatemala | 8525(6096,11492) | 460.94(450.88,471.19) |  | 20423(14489,27718) | 456.55(450.21,462.96) |  | -0.29(-0.54,-0.04) | 139.56(118.33,167.69) |
| Guinea | 47192(34403,62362) | 3386.00(3354.97,3417.28) |  | 94440(67687,125248) | 2777.39(2759.14,2795.75) |  | -0.90(-1.25,-0.55) | 100.12(80.11,124.30) |
| Guinea-Bissau | 6153(4440,8239) | 2607.52(2540.47,2676.19) |  | 10569(7725,14214) | 1961.66(1923.40,2000.64) |  | -0.99(-1.04,-0.93) | 71.76(53.16,89.23) |
| Guyana | 627(444,859) | 298.09(274.34,323.73) |  | 658(481,873) | 318.25(294.15,343.90) |  | -0.33(-0.57,-0.10) | 4.97(-4.64,16.92) |
| Haiti | 50897(36676,66394) | 3200.63(3172.24,3229.25) |  | 92695(67946,122260) | 2586.44(2569.73,2603.25) |  | -0.45(-0.78,-0.11) | 82.12(53.77,106.23) |
| Honduras | 3231(2303,4303) | 306.99(296.05,318.29) |  | 7830(5576,10552) | 273.86(267.73,280.10) |  | -2.20(-2.97,-1.43) | 142.34(122.87,165.86) |
| Iceland | 88(63,117) | 133.63(107.13,165.21) |  | 101(71,136) | 127.12(103.50,154.80) |  | -0.12(-0.16,-0.09) | 14.62(5.68,25.97) |
| India | 2774844(2029467,3722112) | 1360.97(1359.34,1362.59) |  | 3675900(2685675,4990890) | 961.16(960.18,962.15) |  | -3.60(-4.51,-2.69) | 32.47(28.06,36.96) |
| Indonesia | 740364(516268,1002886) | 1505.83(1502.32,1509.34) |  | 1063313(764347,1402597) | 1417.31(1414.62,1420.01) |  | 1.06(0.69,1.44) | 43.62(33.94,53.98) |
| Iraq | 15275(10846,20837) | 375.53(369.32,381.84) |  | 38638(27112,52230) | 363.96(360.31,367.64) |  | -0.16(-0.18,-0.14) | 152.95(132.15,181.18) |
| Italy | 21560(15081,29511) | 150.88(148.87,152.91) |  | 16586(11709,22261) | 142.36(140.14,144.61) |  | -0.18(-0.24,-0.13) | -23.07(-26.94,-18.02) |
| Kazakhstan | 5981(4172,8063) | 140.36(136.77,144.02) |  | 6493(4667,8646) | 136.50(133.17,139.89) |  | -0.79(-1.15,-0.43) | 8.56(-2.03,20.26) |
| Kenya | 282348(202535,372001) | 5297.90(5277.01,5318.86) |  | 319355(232750,423923) | 2367.10(2358.67,2375.57) |  | -4.35(-5.22,-3.48) | 13.11(6.29,20.54) |
| Kiribati | 656(479,890) | 3487.90(3218.56,3777.05) |  | 1063(800,1389) | 3303.83(3106.77,3511.25) |  | 0.29(0.10,0.47) | 62.10(45.94,78.83) |
| South Korea | 55538(38726,74708) | 429.14(425.53,432.78) |  | 46895(33307,61283) | 408.38(404.59,412.20) |  | 0.07(-0.03,0.18) | -15.56(-25.98,-4.00) |
| Latvia | 914(651,1203) | 140.64(131.65,150.11) |  | 493(353,660) | 129.47(117.96,141.93) |  | -0.37(-0.40,-0.34) | -46.07(-51.76,-40.28) |
| Liberia | 40136(29016,54121) | 7007.44(6935.79,7079.81) |  | 83700(59675,111524) | 5992.82(5951.56,6034.32) |  | 1.47(0.82,2.13) | 108.54(85.76,135.08) |
| Lithuania | 1115(770,1516) | 119.84(112.90,127.11) |  | 643(449,857) | 112.66(103.99,121.94) |  | -0.97(-1.23,-0.70) | -42.35(-47.40,-36.28) |
| Madagascar | 199970(145542,255263) | 7154.63(7121.93,7187.46) |  | 328677(245316,433153) | 4460.33(4444.64,4476.06) |  | -2.02(-2.28,-1.76) | 64.36(31.32,96.01) |
| Malawi | 82952(76360,90129) | 3536.32(3511.00,3561.81) |  | 69924(65713,80139) | 1359.92(1349.35,1370.58) |  | -2.26(-2.73,-1.80) | -15.70(-21.61,-5.12) |
| Maldives | 212(150,288) | 440.09(379.05,509.83) |  | 365(260,492) | 314.18(282.30,348.95) |  | -1.16(-1.32,-0.99) | 71.69(47.35,98.85) |
| Mali | 91839(66582,120395) | 4716.83(4685.55,4748.30) |  | 208459(147757,276723) | 3768.04(3751.09,3785.07) |  | 1.01(0.54,1.48) | 126.98(96.39,160.56) |
| Malta | 108(75,144) | 115.35(94.47,139.71) |  | 105(75,139) | 112.72(91.80,137.61) |  | -0.18(-0.22,-0.14) | -2.79(-11.43,6.69) |
| Mauritius | 1960(1338,2668) | 626.73(598.93,655.68) |  | 1846(1320,2481) | 591.63(564.84,619.40) |  | 1.09(0.73,1.46) | -5.86(-15.76,5.86) |
| Moldova | 3588(2525,4826) | 311.99(301.79,322.46) |  | 2336(1648,3139) | 265.10(254.06,276.57) |  | 0.65(0.23,1.08) | -34.91(-42.82,-26.04) |
| Mozambique | 334486(286455,382842) | 10409.95(10373.87,10446.15) |  | 379355(273815,506378) | 4908.97(4892.63,4925.35) |  | -3.61(-4.06,-3.16) | 13.41(-14.00,43.91) |
| Namibia | 13496(9679,18335) | 3953.33(3883.61,4024.19) |  | 23066(16511,31847) | 3410.20(3365.84,3455.06) |  | -2.49(-3.47,-1.49) | 70.91(51.49,90.75) |
| Nauru | 86(61,117) | 3476.06(2765.97,4341.98) |  | 108(81,143) | 3735.04(3056.90,4535.36) |  | 0.87(0.69,1.05) | 26.43(14.64,38.68) |
| Nicaragua | 3311(2316,4432) | 361.95(349.11,375.25) |  | 5969(4217,8155) | 324.18(315.98,332.56) |  | -3.61(-4.92,-2.28) | 80.29(59.51,98.27) |
| Niue | 11(8,15) | 2216.95(1105.28,4014.97) |  | 9(7,11) | 2317.85(1045.50,4497.36) |  | 0.06(0.01,0.11) | -20.86(-29.99,-11.84) |
| Palau | 47(33,64) | 1115.84(816.15,1504.44) |  | 42(32,55) | 1193.59(853.36,1633.39) |  | 0.24(0.16,0.33) | -9.68(-20.21,4.19) |
| Panama | 5410(3747,7407) | 868.43(844.92,892.51) |  | 9423(6825,12578) | 879.31(861.61,897.29) |  | 1.08(0.73,1.43) | 74.17(54.57,98.61) |
| Paraguay | 26166(18569,36087) | 2748.25(2714.23,2782.64) |  | 50443(36244,67324) | 2621.03(2598.09,2644.13) |  | 1.51(1.07,1.96) | 92.78(74.76,114.48) |
| Peru | 65605(45825,89082) | 1149.50(1140.47,1158.59) |  | 100113(70183,138800) | 1021.29(1014.97,1027.65) |  | -3.62(-4.42,-2.82) | 52.60(37.83,68.68) |
| Poland | 13711(9636,18597) | 144.72(142.28,147.20) |  | 11380(8021,15456) | 128.42(126.00,130.89) |  | -0.38(-0.40,-0.36) | -17.00(-21.33,-11.89) |
| Rwanda | 47790(35382,64127) | 2865.44(2838.62,2892.50) |  | 75348(54610,101924) | 2101.71(2086.45,2117.07) |  | -2.04(-2.51,-1.57) | 57.66(41.38,78.13) |
| San Marino | 15(9,25) | 244.00(136.59,404.28) |  | 15(10,19) | 226.50(123.73,382.58) |  | -0.15(-0.24,-0.05) | -3.69(-28.19,22.95) |
| Senegal | 19431(13916,26489) | 1105.27(1089.09,1121.67) |  | 35178(25140,47270) | 882.34(872.91,891.85) |  | -3.21(-3.85,-2.56) | 81.04(62.54,102.10) |
| Serbia | 2912(2019,3990) | 124.53(120.05,129.15) |  | 2466(1736,3316) | 122.58(117.74,127.59) |  | -0.12(-0.15,-0.09) | -15.31(-24.11,-5.38) |
| Seychelles | 77(55,104) | 400.45(312.70,509.69) |  | 85(64,110) | 362.16(289.11,448.40) |  | -0.50(-0.76,-0.24) | 10.61(-2.12,25.31) |
| Sierra Leone | 17065(12407,22896) | 1624.88(1599.55,1650.60) |  | 32424(23253,44773) | 1379.76(1364.20,1395.49) |  | -0.59(-0.66,-0.52) | 90.00(70.46,107.71) |
| Singapore | 3475(2436,4688) | 359.96(348.00,372.26) |  | 4854(3369,6620) | 332.35(322.43,342.57) |  | -0.29(-0.32,-0.27) | 39.68(22.97,59.98) |
| Slovenia | 602(422,799) | 120.09(110.67,130.12) |  | 485(342,660) | 117.31(106.77,128.71) |  | -0.08(-0.10,-0.07) | -19.36(-27.21,-8.83) |
| South Africa | 915902(731987,1131933) | 9189.90(9170.54,9209.29) |  | 693233(501230,928063) | 4395.44(4385.07,4405.83) |  | -2.33(-3.53,-1.12) | -24.31(-35.42,-13.17) |
| South Sudan | 77162(56140,102897) | 5837.44(5793.92,5881.27) |  | 119979(86311,158377) | 5277.53(5246.82,5308.38) |  | 0.22(-0.14,0.57) | 55.49(40.90,75.05) |
| Sudan | 71810(49622,96724) | 1540.28(1528.62,1552.02) |  | 175457(117910,243252) | 1553.76(1546.36,1561.20) |  | 0.37(-0.04,0.77) | 144.34(114.71,183.06) |
| Suriname | 272(196,370) | 274.24(241.87,310.10) |  | 420(312,555) | 290.44(263.30,319.64) |  | -0.07(-0.20,0.05) | 54.17(37.96,70.22) |
| Tajikistan | 1669(1145,2292) | 132.26(125.53,139.37) |  | 3147(2264,4180) | 119.81(115.62,124.13) |  | -1.44(-1.86,-1.02) | 88.48(66.94,112.49) |
| Congo (Brazzaville) | 21593(15584,29182) | 3862.96(3809.01,3917.60) |  | 46631(34324,63856) | 3257.70(3227.98,3287.65) |  | -0.72(-0.82,-0.61) | 115.95(93.79,136.59) |
| The Gambia | 8266(5965,11242) | 3510.02(3429.88,3592.07) |  | 19415(13951,26272) | 3095.80(3050.69,3141.53) |  | -1.04(-1.27,-0.81) | 134.87(114.55,159.80) |
| Marshall Islands | 279(201,378) | 2853.70(2511.25,3243.65) |  | 429(317,559) | 2906.66(2636.96,3197.83) |  | 0.70(0.35,1.06) | 53.67(37.55,72.55) |
| Niger | 10362(7671,13822) | 582.26(570.65,594.08) |  | 22136(16146,30200) | 406.59(400.89,412.36) |  | -2.60(-3.06,-2.14) | 113.62(92.52,133.47) |
| Philippines | 147704(102790,200901) | 923.32(918.48,928.17) |  | 306923(222947,417938) | 1026.53(1022.87,1030.19) |  | 1.48(1.13,1.83) | 107.80(96.43,120.66) |
| Myanmar | 71597(49492,100963) | 658.78(653.82,663.77) |  | 95858(67842,129493) | 633.27(629.26,637.30) |  | 0.99(0.69,1.29) | 33.89(18.91,50.42) |
| Trinidad and Tobago | 1662(1160,2296) | 524.53(499.32,550.83) |  | 1654(1198,2213) | 494.29(470.50,519.03) |  | -1.53(-1.94,-1.12) | -0.50(-10.44,11.41) |
| Tunisia | 7809(5402,10973) | 373.73(365.22,382.41) |  | 10928(7782,14582) | 358.96(352.21,365.82) |  | -0.16(-0.17,-0.15) | 39.93(22.86,59.91) |
| Turkey | 85126(58587,114560) | 593.26(589.19,597.35) |  | 120296(82651,161854) | 559.45(556.29,562.63) |  | -0.21(-0.22,-0.19) | 41.31(26.15,60.22) |
| Uganda | 191440(137186,257121) | 4874.74(4851.40,4898.18) |  | 525433(379252,708929) | 4911.91(4897.98,4925.89) |  | 1.21(0.62,1.79) | 174.46(148.68,206.41) |
| Uzbekistan | 8344(5725,11222) | 162.61(158.91,166.39) |  | 12716(8922,17215) | 140.22(137.79,142.69) |  | -0.63(-0.69,-0.58) | 52.41(35.89,72.39) |
| Vanuatu | 1135(817,1546) | 3193.68(3004.13,3394.12) |  | 2595(1927,3536) | 3300.96(3173.58,3432.53) |  | 0.53(0.33,0.74) | 128.72(107.68,157.07) |
| Yemen | 8259(5697,11528) | 298.27(291.63,305.05) |  | 24042(16796,33596) | 287.48(283.78,291.21) |  | -0.22(-0.26,-0.19) | 191.09(163.37,220.91) |
| Zambia | 116822(103884,130941) | 6184.60(6146.63,6222.78) |  | 227103(163476,305314) | 4500.56(4481.34,4519.87) |  | -1.81(-2.17,-1.46) | 94.40(46.43,146.08) |
| Zimbabwe | 113416(75122,157985) | 4550.34(4522.45,4578.40) |  | 107994(78715,145738) | 2615.96(2600.07,2631.94) |  | -2.41(-3.00,-1.81) | -4.78(-29.51,25.41) |
| Romania | 15676(11116,20800) | 282.81(278.37,287.31) |  | 9896(6985,13071) | 249.54(244.51,254.66) |  | -0.21(-0.41,0.00) | -36.87(-44.83,-28.69) |
| Russia | 65244(45969,89840) | 170.58(169.26,171.90) |  | 53295(38152,72015) | 156.59(155.21,157.98) |  | -0.36(-0.48,-0.24) | -18.31(-23.24,-11.99) |
| Saint Kitts and Nevis | 264(183,357) | 2537.05(2225.91,2892.61) |  | 334(239,452) | 2149.03(1924.17,2394.07) |  | -0.39(-0.47,-0.32) | 26.66(13.40,42.53) |
| Saint Lucia | 580(411,799) | 1657.26(1519.77,1806.02) |  | 780(572,1019) | 1725.67(1606.07,1852.27) |  | 0.87(0.63,1.11) | 34.59(17.90,55.41) |
| Saint Vincent and the Grenadines | 236(167,331) | 871.92(759.09,1000.50) |  | 229(163,307) | 831.73(727.22,947.49) |  | -0.15(-0.18,-0.12) | -3.32(-14.82,8.67) |
| Slovakia | 997(688,1328) | 75.12(70.52,79.96) |  | 995(708,1334) | 79.42(74.40,84.73) |  | 0.06(-0.03,0.15) | -0.20(-10.84,11.83) |
| Viet Nam | 57688(39870,80437) | 317.52(314.82,320.24) |  | 75938(53827,103333) | 299.04(296.90,301.20) |  | -0.29(-0.34,-0.24) | 31.63(17.33,48.60) |
| Solomon Islands | 2818(2035,3791) | 3861.01(3712.01,4015.34) |  | 6493(4677,8649) | 3812.44(3719.37,3907.40) |  | -0.07(-0.30,0.16) | 130.39(106.92,159.97) |
| Eritrea | 16804(12140,22438) | 2103.12(2070.37,2136.32) |  | 28891(21067,38595) | 1709.80(1689.81,1729.98) |  | -1.21(-1.45,-0.97) | 71.93(53.39,92.67) |
| Israel | 1636(1134,2207) | 134.37(127.89,141.13) |  | 2840(2031,3819) | 129.45(124.72,134.31) |  | -0.13(-0.17,-0.09) | 73.59(55.98,91.16) |
| Kuwait | 1602(1084,2219) | 365.28(346.88,384.68) |  | 5164(3604,7013) | 349.23(339.28,359.47) |  | -0.15(-0.17,-0.13) | 222.28(182.50,275.65) |
| Libya | 3389(2333,4651) | 373.12(359.84,386.86) |  | 7107(5005,9386) | 360.27(351.93,368.77) |  | -0.15(-0.16,-0.14) | 109.72(86.89,140.83) |
| Qatar | 544(354,763) | 660.23(604.10,721.56) |  | 3674(2385,5293) | 634.67(612.97,657.06) |  | -0.15(-0.18,-0.13) | 575.10(491.18,689.01) |
| Oman | 382(271,539) | 110.28(99.15,122.54) |  | 1116(767,1537) | 107.50(101.18,114.16) |  | -1.97(-2.62,-1.32) | 192.52(163.92,227.05) |
| Switzerland | 1698(1177,2290) | 95.44(90.92,100.15) |  | 1780(1236,2422) | 91.96(87.65,96.44) |  | 0.06(-0.03,0.16) | 4.86(-4.70,16.30) |
| Syria | 11886(8332,16155) | 432.14(423.96,440.46) |  | 13809(10027,18239) | 381.91(375.10,388.83) |  | -0.44(-0.48,-0.40) | 16.17(3.20,32.47) |
| Taiwan (province of China) | 17188(12207,22514) | 302.55(297.98,307.18) |  | 15198(10891,20135) | 270.24(265.82,274.72) |  | -0.45(-0.62,-0.28) | -11.58(-26.01,2.18) |
| Togo | 16330(11879,21950) | 1865.55(1835.88,1895.66) |  | 33460(24457,46079) | 1529.04(1512.54,1545.69) |  | -3.62(-4.53,-2.70) | 104.90(80.56,130.99) |
| Tokelau | 9(6,12) | 2447.90(1094.14,4920.00) |  | 8(6,10) | 2402.25(1018.41,4865.82) |  | -0.09(-0.13,-0.05) | -12.81(-24.10,0.89) |
| Turkmenistan | 1619(1107,2169) | 177.35(168.30,186.86) |  | 1929(1378,2622) | 152.01(145.29,158.97) |  | -0.53(-0.59,-0.48) | 19.18(6.88,33.67) |
| Tuvalu | 37(25,51) | 1493.30(1048.58,2074.90) |  | 46(33,62) | 1542.81(1124.79,2075.69) |  | -0.04(-0.17,0.08) | 24.85(11.89,44.15) |
| Ukraine | 18307(12845,25690) | 143.04(140.97,145.14) |  | 13949(10147,18680) | 138.34(135.96,140.76) |  | -0.37(-0.51,-0.23) | -23.80(-31.25,-14.86) |
| Comoros | 5208(3781,7022) | 4841.44(4705.55,4980.84) |  | 5242(3804,7008) | 2647.17(2575.43,2720.52) |  | -3.54(-4.10,-2.98) | 0.65(-22.01,20.38) |
| United Arab Emirates | 451(313,635) | 124.10(112.24,137.36) |  | 1939(1348,2644) | 120.33(114.38,126.54) |  | -2.23(-3.01,-1.45) | 330.02(257.15,423.34) |
| UK | 28745(20550,38788) | 201.82(199.48,204.18) |  | 31143(22669,41263) | 206.31(204.00,208.63) |  | 0.28(0.14,0.43) | 8.34(4.57,13.07) |
| Mexico | 113374(79895,153695) | 516.88(513.78,520.01) |  | 152088(109873,207238) | 435.08(432.89,437.27) |  | -0.54(-0.98,-0.09) | 34.15(26.18,44.32) |
| Tanzania | 438322(399725,478773) | 6706.73(6685.88,6727.65) |  | 621862(453292,829263) | 4109.22(4098.72,4119.75) |  | -3.98(-4.65,-3.30) | 41.87(3.66,81.18) |
| USA | 349415(248521,481938) | 510.38(508.68,512.08) |  | 359965(259841,491955) | 475.64(474.08,477.19) |  | -0.42(-0.71,-0.14) | 3.02(0.35,5.87) |
| Virgin Islands | 248(179,322) | 884.36(777.22,1002.53) |  | 157(118,201) | 947.23(803.20,1110.95) |  | 0.09(0.03,0.16) | -36.79(-42.83,-30.19) |

ASR, age standardized rate; CI, confidence interval; EAPC, estimated annual percentage change; UI, uncertainty interval; SDI, socio-demographic index.

## Table S3 The DALY of syphilis cases and rates among WCBA in 1990 and 2021 across 204 countries, and the trends from 1990 to 2021

| **Location** | **1990** | |  | **2021** | |  | **1990-2021** | |
| --- | --- | --- | --- | --- | --- | --- | --- | --- |
|  | **Cases number** | **ASR per 100,000** |  | **Cases number** | **ASR per 100,000** |  | **EAPC** | **Cases change(%)** |
|  | **No.  (95% UI)** | **No. (95% CI)** |  | **No.  (95% UI)** | **No. (95% CI)** |  | **No. (95% CI)** | **No. (95% UI)** |
| American Samoa | 0(0,0) | 0.89(0.00,53.73) |  | 0(0,1) | 3.10(0.00,43.65) |  | 3.61(3.22,4.00) | 241.11(83.93,536.23) |
| Antigua and Barbuda | 0(0,0) | 0.58(0.00,35.45) |  | 0(0,0) | 0.86(0.00,19.91) |  | 2.11(1.72,2.50) | 130.16(83.18,186.06) |
| Egypt | 69(46,111) | 0.54(0.41,0.68) |  | 68(44,105) | 0.26(0.20,0.34) |  | -1.98(-2.19,-1.77) | -1.94(-38.61,51.14) |
| Argentina | 55(36,91) | 0.68(0.51,0.89) |  | 87(58,144) | 0.73(0.59,0.91) |  | 0.74(0.45,1.04) | 60.04(31.02,94.35) |
| Australia | 10(8,16) | 0.23(0.11,0.43) |  | 7(4,14) | 0.12(0.05,0.25) |  | -1.79(-2.12,-1.46) | -28.87(-51.37,2.30) |
| Barbados | 1(1,1) | 1.05(0.01,9.12) |  | 1(1,1) | 1.09(0.01,8.04) |  | 0.98(0.07,1.89) | 14.17(-17.38,55.13) |
| Belize | 0(0,0) | 0.49(0.00,18.43) |  | 1(1,1) | 0.85(0.02,5.30) |  | 2.08(1.49,2.67) | 416.70(304.26,552.78) |
| Bermuda | 0(0,0) | 0.47(0.00,30.42) |  | 0(0,0) | 0.50(0.00,39.27) |  | 0.50(0.24,0.77) | -14.93(-36.28,11.61) |
| Venezuela | 63(48,91) | 1.34(1.02,1.74) |  | 114(83,162) | 1.65(1.36,1.99) |  | 1.43(1.13,1.74) | 81.07(44.72,126.65) |
| Bosnia and Herzegovina | 1(1,2) | 0.11(0.01,0.58) |  | 1(0,1) | 0.09(0.00,0.83) |  | -1.12(-1.31,-0.92) | -48.79(-62.52,-29.87) |
| Brunei | 0(0,0) | 0.35(0.00,11.09) |  | 1(0,1) | 0.40(0.00,4.31) |  | 0.51(0.20,0.81) | 126.48(55.38,209.57) |
| Burkina Faso | 68(48,95) | 3.22(2.48,4.14) |  | 143(102,199) | 2.56(2.15,3.06) |  | -0.33(-0.50,-0.16) | 110.76(60.63,158.72) |
| Canada | 16(13,19) | 0.21(0.12,0.35) |  | 13(10,16) | 0.15(0.08,0.26) |  | -1.14(-1.38,-0.90) | -18.52(-29.86,-5.57) |
| Central African Republic | 104(75,145) | 16.41(13.28,20.13) |  | 228(161,321) | 16.56(14.41,18.97) |  | -0.46(-0.70,-0.21) | 119.18(69.93,185.53) |
| Dominica | 0(0,0) | 0.84(0.00,32.18) |  | 0(0,1) | 2.90(0.00,29.75) |  | 4.00(3.53,4.48) | 237.49(96.56,495.07) |
| The Bahamas | 1(1,1) | 1.04(0.01,9.38) |  | 2(1,3) | 1.85(0.22,6.84) |  | 2.21(1.83,2.60) | 175.90(109.77,284.71) |
| Cook Islands | 0(0,0) | 0.90(0.00,111.57) |  | 0(0,0) | 1.02(0.00,102.04) |  | 0.28(0.07,0.49) | 10.31(-17.20,49.59) |
| Czechia | 6(5,8) | 0.24(0.09,0.53) |  | 4(3,5) | 0.15(0.03,0.46) |  | -1.23(-1.46,-0.99) | -42.76(-57.86,-15.10) |
| North Korea | 17(11,27) | 0.30(0.17,0.50) |  | 25(16,37) | 0.36(0.23,0.54) |  | 0.37(0.20,0.54) | 48.22(-2.28,115.83) |
| Sao Tome and Principe | 1(0,1) | 2.20(0.00,28.49) |  | 1(1,2) | 1.94(0.06,12.37) |  | -1.33(-1.65,-1.01) | 92.78(39.07,154.45) |
| DR Congo | 1103(767,1528) | 13.03(12.23,13.86) |  | 1496(1009,2281) | 7.08(6.71,7.47) |  | -3.03(-3.44,-2.62) | 35.57(-2.86,86.61) |
| Timor-Leste | 2(2,3) | 1.29(0.19,5.10) |  | 4(3,5) | 1.11(0.27,3.28) |  | -0.75(-1.02,-0.47) | 59.82(27.53,108.41) |
| Sri Lanka | 34(26,47) | 0.73(0.51,1.04) |  | 23(15,32) | 0.40(0.25,0.61) |  | -2.20(-2.43,-1.98) | -32.77(-55.66,-3.67) |
| Dominican Republic | 65(45,88) | 3.68(2.81,4.79) |  | 102(63,158) | 3.57(2.91,4.35) |  | 0.47(0.23,0.70) | 57.26(-5.37,157.71) |
| Uruguay | 6(4,9) | 0.75(0.26,1.69) |  | 6(4,9) | 0.70(0.25,1.57) |  | -0.02(-0.15,0.10) | 3.38(-15.63,23.99) |
| Ethiopia | 2886(1873,4433) | 24.57(23.64,25.53) |  | 2163(1340,4468) | 7.45(7.13,7.79) |  | -4.64(-5.06,-4.22) | -25.07(-50.58,38.41) |
| Nepal | 270(178,381) | 6.00(5.28,6.79) |  | 251(176,350) | 2.82(2.48,3.20) |  | -2.52(-2.63,-2.41) | -6.81(-38.57,35.67) |
| Germany | 27(21,36) | 0.14(0.09,0.20) |  | 12(8,20) | 0.07(0.04,0.13) |  | -2.06(-2.28,-1.84) | -54.10(-64.82,-40.83) |
| Nigeria | 832(525,1194) | 4.07(3.78,4.38) |  | 1806(1183,2629) | 3.10(2.95,3.25) |  | -0.88(-1.14,-0.62) | 117.00(83.67,160.34) |
| Somalia | 281(180,502) | 16.23(14.34,18.34) |  | 674(429,1173) | 13.40(12.36,14.51) |  | -0.13(-0.52,0.25) | 140.12(59.62,252.24) |
| Federated States of Micronesia | 1(1,2) | 4.47(0.08,36.97) |  | 1(1,2) | 5.19(0.28,25.71) |  | 0.37(0.13,0.61) | 34.16(-9.19,113.41) |
| Brazil | 736(603,958) | 1.93(1.79,2.08) |  | 1165(899,1591) | 1.97(1.85,2.08) |  | -0.73(-1.33,-0.13) | 58.28(43.39,74.57) |
| France | 24(18,35) | 0.17(0.11,0.25) |  | 14(9,25) | 0.10(0.05,0.17) |  | -1.26(-1.45,-1.08) | -39.63(-52.58,-25.27) |
| Gabon | 17(11,24) | 7.85(4.37,13.58) |  | 18(13,27) | 3.81(2.24,6.16) |  | -4.24(-4.89,-3.59) | 9.15(-19.15,41.43) |
| Georgia | 7(5,9) | 0.48(0.18,1.04) |  | 2(1,3) | 0.21(0.02,0.98) |  | -4.11(-4.76,-3.46) | -74.32(-82.72,-61.24) |
| Luxembourg | 0(0,0) | 0.16(0.00,5.67) |  | 0(0,0) | 0.07(0.00,3.69) |  | -2.51(-2.66,-2.37) | -27.80(-45.58,-8.26) |
| Greenland | 0(0,0) | 0.18(0.00,34.23) |  | 0(0,0) | 0.29(0.00,34.41) |  | 1.21(0.75,1.68) | 40.45(-5.24,124.18) |
| Grenada | 0(0,0) | 1.54(0.00,34.60) |  | 1(0,1) | 2.20(0.00,20.88) |  | 2.53(2.18,2.89) | 92.00(56.80,135.59) |
| Guam | 0(0,1) | 0.73(0.00,18.93) |  | 0(0,1) | 0.96(0.00,13.29) |  | 0.37(0.01,0.73) | 39.90(6.42,107.70) |
| Jordan | 1(0,3) | 0.18(0.01,1.10) |  | 5(2,11) | 0.16(0.05,0.39) |  | -0.46(-0.54,-0.38) | 234.81(111.17,427.61) |
| Greece | 3(2,4) | 0.11(0.02,0.34) |  | 3(2,4) | 0.12(0.02,0.41) |  | 0.95(0.70,1.21) | 1.85(-12.28,18.53) |
| Hungary | 19(16,24) | 0.74(0.44,1.16) |  | 9(7,13) | 0.38(0.17,0.79) |  | -1.77(-2.03,-1.52) | -53.70(-66.97,-33.47) |
| Papua New Guinea | 41(24,63) | 4.33(3.06,6.03) |  | 106(66,165) | 4.10(3.35,4.98) |  | -0.75(-1.12,-0.37) | 161.66(75.00,312.32) |
| Samoa | 0(0,0) | 0.71(0.00,17.70) |  | 0(0,1) | 1.01(0.00,11.58) |  | 0.54(0.11,0.97) | 100.71(51.94,170.24) |
| Ireland | 1(1,2) | 0.15(0.01,0.77) |  | 1(1,2) | 0.08(0.00,0.54) |  | -1.72(-1.97,-1.47) | -27.12(-40.91,-11.03) |
| Afghanistan | 21(14,36) | 0.98(0.58,1.63) |  | 50(32,83) | 0.69(0.50,0.94) |  | -1.46(-1.71,-1.22) | 131.79(105.92,169.44) |
| Iran | 18(10,31) | 0.14(0.08,0.24) |  | 31(16,60) | 0.13(0.09,0.19) |  | -0.08(-0.38,0.23) | 76.04(36.06,110.08) |
| Mauritania | 32(21,46) | 6.66(4.46,9.76) |  | 30(21,43) | 2.78(1.85,4.08) |  | -2.47(-2.64,-2.31) | -5.34(-28.22,29.57) |
| Pakistan | 850(537,1294) | 3.78(3.52,4.06) |  | 2185(1501,3109) | 3.70(3.54,3.86) |  | -0.61(-0.86,-0.36) | 156.97(89.69,247.37) |
| Jamaica | 8(5,16) | 1.48(0.63,3.14) |  | 14(10,20) | 1.77(0.96,3.02) |  | 1.35(0.96,1.74) | 61.54(11.23,151.56) |
| Japan | 56(32,102) | 0.17(0.13,0.22) |  | 45(27,76) | 0.17(0.12,0.23) |  | -0.02(-0.20,0.15) | -20.05(-27.77,-11.14) |
| Bahrain | 0(0,1) | 0.22(0.00,7.21) |  | 1(0,2) | 0.23(0.00,1.68) |  | 0.24(0.14,0.33) | 185.91(97.39,320.13) |
| Belgium | 4(3,5) | 0.15(0.04,0.41) |  | 2(2,3) | 0.08(0.01,0.32) |  | -1.58(-1.80,-1.37) | -38.90(-50.24,-26.59) |
| Bhutan | 12(7,17) | 8.31(4.02,16.14) |  | 8(6,12) | 4.06(1.78,8.18) |  | -3.14(-3.43,-2.85) | -27.84(-47.62,2.85) |
| Cambodia | 17(11,29) | 0.67(0.38,1.13) |  | 21(13,40) | 0.46(0.29,0.72) |  | -2.17(-2.54,-1.80) | 25.92(-25.11,141.33) |
| Denmark | 4(3,4) | 0.27(0.07,0.75) |  | 2(1,2) | 0.13(0.01,0.56) |  | -2.68(-3.01,-2.36) | -52.76(-63.38,-36.85) |
| Eswatini | 19(13,26) | 9.68(5.61,16.15) |  | 17(11,25) | 5.41(3.11,9.08) |  | -1.68(-1.90,-1.46) | -9.41(-38.83,31.61) |
| Lesotho | 30(21,44) | 8.05(5.40,11.67) |  | 40(27,58) | 7.88(5.57,10.99) |  | 0.12(-0.07,0.31) | 31.28(-7.31,86.04) |
| Morocco | 124(81,187) | 1.97(1.63,2.37) |  | 120(70,203) | 1.24(1.02,1.48) |  | 0.11(-0.86,1.08) | -3.42(-20.52,19.43) |
| Norway | 1(1,2) | 0.12(0.01,0.60) |  | 2(1,2) | 0.13(0.01,0.59) |  | -0.47(-0.85,-0.10) | 25.44(17.35,35.26) |
| Saudi Arabia | 5(3,8) | 0.16(0.05,0.44) |  | 10(5,17) | 0.09(0.04,0.18) |  | -1.95(-2.09,-1.82) | 90.72(16.58,177.99) |
| Spain | 16(13,22) | 0.17(0.10,0.28) |  | 9(6,14) | 0.08(0.04,0.17) |  | -2.08(-2.29,-1.86) | -46.30(-58.09,-33.30) |
| Sweden | 2(2,3) | 0.11(0.02,0.39) |  | 2(1,3) | 0.09(0.01,0.35) |  | -1.18(-1.86,-0.49) | -13.56(-28.99,0.75) |
| Thailand | 78(48,123) | 0.49(0.39,0.62) |  | 64(44,91) | 0.39(0.30,0.50) |  | -2.42(-3.08,-1.75) | -17.42(-48.85,27.49) |
| Netherlands | 6(4,8) | 0.14(0.05,0.33) |  | 3(2,5) | 0.08(0.02,0.25) |  | -1.58(-1.77,-1.39) | -45.17(-54.73,-35.70) |
| Tonga | 0(0,0) | 0.91(0.00,24.90) |  | 0(0,1) | 1.77(0.00,20.69) |  | 1.45(1.16,1.74) | 136.10(77.74,222.09) |
| Kyrgyzstan | 11(10,14) | 1.13(0.54,2.23) |  | 4(3,5) | 0.24(0.07,0.64) |  | -5.65(-6.74,-4.55) | -63.20(-72.75,-49.69) |
| Laos | 12(9,18) | 1.30(0.66,2.37) |  | 17(12,26) | 0.87(0.51,1.42) |  | -1.33(-1.57,-1.08) | 39.28(3.47,97.44) |
| Lebanon | 3(2,5) | 0.39(0.07,1.25) |  | 3(2,6) | 0.21(0.05,0.66) |  | -2.01(-2.19,-1.84) | 13.40(-30.13,64.41) |
| Malaysia | 19(14,28) | 0.45(0.27,0.73) |  | 26(19,37) | 0.31(0.20,0.46) |  | -1.30(-1.53,-1.06) | 33.83(-5.26,84.12) |
| Mongolia | 18(10,32) | 3.66(2.06,6.21) |  | 18(12,25) | 2.10(1.24,3.37) |  | -2.11(-2.52,-1.70) | 1.41(-35.55,68.37) |
| Montenegro | 0(0,0) | 0.15(0.00,3.08) |  | 0(0,0) | 0.13(0.00,3.34) |  | -0.58(-0.88,-0.27) | -15.01(-37.59,12.15) |
| New Zealand | 2(1,3) | 0.20(0.02,0.82) |  | 2(1,3) | 0.13(0.01,0.59) |  | -1.10(-1.75,-0.46) | -13.54(-34.81,14.97) |
| North Macedonia | 1(1,2) | 0.22(0.01,1.20) |  | 1(0,2) | 0.12(0.00,1.20) |  | -2.52(-2.74,-2.29) | -37.43(-68.66,30.77) |
| Northern Mariana Islands | 0(0,0) | 1.24(0.00,54.63) |  | 0(0,0) | 2.18(0.00,44.95) |  | 1.16(0.50,1.82) | 64.97(6.06,183.84) |
| Palestine | 1(1,2) | 0.24(0.00,1.82) |  | 3(1,5) | 0.24(0.05,0.78) |  | 0.33(0.01,0.65) | 196.87(94.17,348.32) |
| Algeria | 11(6,22) | 0.20(0.10,0.38) |  | 16(7,34) | 0.14(0.08,0.23) |  | -2.12(-2.72,-1.53) | 38.97(-5.18,100.93) |
| Bangladesh | 1078(718,1556) | 4.71(4.42,5.02) |  | 722(515,1065) | 1.60(1.48,1.72) |  | -4.41(-4.76,-4.05) | -33.06(-54.03,-1.59) |
| China | 2031(1376,2854) | 0.68(0.65,0.71) |  | 921(615,1444) | 0.27(0.25,0.29) |  | -3.49(-3.69,-3.28) | -54.63(-66.88,-36.78) |
| Bolivia | 70(46,105) | 4.57(3.54,5.84) |  | 62(45,89) | 1.97(1.51,2.54) |  | -3.82(-4.24,-3.40) | -12.40(-38.45,26.76) |
| Portugal | 4(4,6) | 0.17(0.05,0.43) |  | 2(1,3) | 0.08(0.01,0.35) |  | -2.11(-2.26,-1.95) | -51.64(-61.75,-38.92) |
| Andorra | 0(0,0) | 0.33(0.00,31.31) |  | 0(0,0) | 0.25(0.00,29.31) |  | -0.74(-0.93,-0.54) | 6.68(-36.85,79.31) |
| Monaco | 0(0,0) | 0.21(0.00,90.56) |  | 0(0,0) | 0.16(0.00,67.03) |  | -0.83(-1.00,-0.66) | -23.02(-53.38,40.25) |
| Puerto Rico | 6(4,9) | 0.64(0.24,1.41) |  | 6(4,9) | 0.80(0.29,1.77) |  | 1.09(0.72,1.45) | 1.65(-20.15,30.19) |
| Albania | 2(1,3) | 0.25(0.03,1.11) |  | 1(0,2) | 0.13(0.00,0.93) |  | -1.86(-2.03,-1.69) | -59.76(-81.85,-12.32) |
| Angola | 305(208,425) | 13.42(11.90,15.12) |  | 514(338,774) | 6.75(6.16,7.39) |  | -2.43(-2.60,-2.26) | 68.38(16.05,130.94) |
| Armenia | 8(7,10) | 0.95(0.40,2.06) |  | 1(1,2) | 0.15(0.01,0.97) |  | -5.93(-6.82,-5.04) | -85.73(-90.52,-75.30) |
| Austria | 3(3,5) | 0.17(0.04,0.50) |  | 2(2,3) | 0.11(0.01,0.43) |  | -1.62(-1.75,-1.50) | -35.24(-46.60,-25.02) |
| Azerbaijan | 31(23,42) | 1.67(1.10,2.49) |  | 23(12,42) | 0.83(0.52,1.27) |  | -3.21(-3.48,-2.93) | -25.93(-56.39,25.67) |
| Belarus | 15(12,19) | 0.60(0.33,1.00) |  | 11(8,15) | 0.46(0.22,0.88) |  | -2.08(-2.56,-1.59) | -25.76(-49.60,5.66) |
| Benin | 36(25,50) | 3.22(2.21,4.61) |  | 69(50,98) | 2.10(1.62,2.71) |  | -2.60(-3.10,-2.10) | 91.35(56.21,146.14) |
| Botswana | 35(24,50) | 10.95(7.45,15.86) |  | 31(21,46) | 4.53(3.07,6.50) |  | -3.61(-4.57,-2.65) | -11.77(-34.48,26.67) |
| Bulgaria | 3(2,4) | 0.14(0.03,0.43) |  | 2(1,2) | 0.10(0.01,0.54) |  | -0.66(-0.83,-0.50) | -44.04(-60.33,-17.66) |
| Burundi | 98(57,194) | 7.22(5.81,8.96) |  | 140(80,303) | 4.16(3.47,4.99) |  | -3.25(-4.01,-2.48) | 43.06(-15.92,150.70) |
| Cabo Verde | 1(1,1) | 1.10(0.01,10.62) |  | 1(1,2) | 0.78(0.03,4.56) |  | -2.52(-3.00,-2.03) | 32.49(-8.19,77.67) |
| Cameroon | 263(175,374) | 10.80(9.48,12.29) |  | 360(214,529) | 4.52(4.05,5.03) |  | -2.52(-2.73,-2.31) | 36.95(-6.66,101.71) |
| Chad | 109(73,158) | 8.15(6.64,9.96) |  | 242(149,355) | 6.17(5.38,7.07) |  | -0.98(-1.43,-0.53) | 121.60(52.58,223.07) |
| Chile | 17(12,24) | 0.46(0.26,0.76) |  | 18(13,27) | 0.38(0.22,0.61) |  | -1.21(-1.63,-0.79) | 8.36(-11.17,30.62) |
| Colombia | 117(100,141) | 1.39(1.14,1.68) |  | 147(118,189) | 1.12(0.95,1.32) |  | -0.21(-0.58,0.16) | 26.01(2.38,55.20) |
| Costa Rica | 6(5,8) | 0.84(0.30,2.01) |  | 12(10,15) | 0.95(0.50,1.66) |  | 0.67(0.28,1.05) | 102.28(65.58,153.77) |
| Croatia | 1(1,2) | 0.12(0.01,0.56) |  | 1(0,1) | 0.07(0.00,0.69) |  | -1.44(-1.65,-1.22) | -53.03(-66.89,-30.78) |
| Cuba | 33(28,42) | 1.11(0.76,1.58) |  | 56(46,69) | 2.14(1.61,2.81) |  | 2.32(1.46,3.19) | 70.38(34.75,118.24) |
| Cyprus | 0(0,1) | 0.17(0.00,2.32) |  | 0(0,1) | 0.09(0.00,1.95) |  | -2.42(-2.69,-2.15) | 7.67(-43.09,63.32) |
| Ivory Coast | 153(99,220) | 5.42(4.55,6.45) |  | 227(157,311) | 3.35(2.92,3.84) |  | -1.45(-1.56,-1.35) | 48.25(10.05,102.69) |
| Djibouti | 7(4,12) | 7.18(2.76,16.78) |  | 19(12,34) | 5.95(3.59,9.42) |  | -0.84(-1.24,-0.44) | 165.54(62.63,316.95) |
| Ecuador | 34(24,52) | 1.34(0.91,1.92) |  | 40(25,67) | 0.83(0.59,1.14) |  | -1.13(-1.35,-0.91) | 15.99(-9.87,51.04) |
| El Salvador | 7(5,9) | 0.57(0.22,1.28) |  | 7(5,10) | 0.42(0.17,0.86) |  | -0.99(-1.23,-0.75) | 5.15(-22.38,34.41) |
| Equatorial Guinea | 18(12,24) | 18.08(10.43,29.84) |  | 20(13,31) | 5.56(3.34,8.96) |  | -4.11(-4.51,-3.70) | 13.62(-24.65,63.56) |
| Estonia | 4(3,5) | 1.02(0.27,2.72) |  | 1(1,2) | 0.47(0.03,2.60) |  | -4.02(-4.51,-3.54) | -65.01(-74.16,-51.30) |
| Fiji | 8(5,12) | 4.22(1.76,8.83) |  | 9(6,15) | 4.02(1.85,7.69) |  | 0.09(-0.01,0.19) | 17.02(-15.37,60.92) |
| Finland | 5(5,6) | 0.40(0.13,0.99) |  | 2(2,3) | 0.19(0.03,0.71) |  | -1.93(-2.16,-1.71) | -57.51(-65.50,-48.60) |
| Ghana | 153(95,221) | 4.22(3.56,5.00) |  | 284(201,395) | 3.06(2.71,3.45) |  | -1.10(-1.39,-0.81) | 86.21(33.88,163.45) |
| Guatemala | 31(27,37) | 1.77(1.19,2.59) |  | 54(44,66) | 1.27(0.95,1.68) |  | -0.69(-1.15,-0.23) | 73.48(43.82,112.51) |
| Guinea | 95(61,134) | 6.86(5.53,8.47) |  | 169(107,238) | 4.96(4.22,5.83) |  | -1.15(-1.40,-0.89) | 77.33(20.73,144.10) |
| Guinea-Bissau | 18(11,28) | 7.77(4.53,12.90) |  | 25(15,37) | 4.56(2.89,7.06) |  | -1.84(-2.04,-1.63) | 33.03(-20.09,106.60) |
| Guyana | 3(3,4) | 1.87(0.41,5.97) |  | 6(4,9) | 3.18(1.19,7.00) |  | 2.17(1.30,3.06) | 79.52(18.73,161.79) |
| Haiti | 165(114,233) | 10.80(9.17,12.69) |  | 278(171,404) | 7.90(6.99,8.90) |  | -1.00(-1.16,-0.84) | 68.86(14.28,138.73) |
| Honduras | 35(23,55) | 3.58(2.45,5.11) |  | 52(29,94) | 1.94(1.44,2.56) |  | -2.59(-2.88,-2.31) | 46.51(-25.79,168.92) |
| Iceland | 0(0,0) | 0.13(0.00,7.36) |  | 0(0,0) | 0.08(0.00,5.61) |  | -1.28(-1.47,-1.08) | -22.58(-38.46,-7.16) |
| India | 11957(8502,15323) | 6.08(5.97,6.19) |  | 11291(7794,14987) | 3.02(2.97,3.08) |  | -3.24(-3.74,-2.74) | -5.58(-21.71,14.84) |
| Indonesia | 1125(667,1707) | 2.36(2.22,2.51) |  | 1859(1176,2702) | 2.47(2.36,2.58) |  | 0.57(0.35,0.80) | 65.18(43.92,95.93) |
| Iraq | 13(8,24) | 0.33(0.17,0.61) |  | 21(12,37) | 0.21(0.13,0.32) |  | -2.00(-2.24,-1.75) | 64.12(-8.72,140.48) |
| Italy | 20(15,29) | 0.14(0.09,0.22) |  | 14(10,21) | 0.10(0.05,0.18) |  | -1.02(-1.18,-0.87) | -30.69(-37.48,-19.32) |
| Kazakhstan | 38(34,43) | 0.94(0.66,1.32) |  | 15(12,20) | 0.31(0.18,0.53) |  | -3.96(-5.52,-2.38) | -59.50(-68.29,-45.89) |
| Kenya | 446(308,629) | 8.15(7.36,9.03) |  | 790(546,1175) | 5.75(5.34,6.18) |  | -1.98(-2.46,-1.50) | 77.07(41.01,147.21) |
| Kiribati | 1(1,2) | 7.14(0.33,42.76) |  | 2(2,4) | 7.68(1.21,27.63) |  | 0.30(0.05,0.56) | 85.79(37.64,177.78) |
| South Korea | 30(18,48) | 0.24(0.16,0.35) |  | 26(16,44) | 0.21(0.13,0.32) |  | -0.28(-0.38,-0.19) | -13.73(-37.20,13.58) |
| Latvia | 9(7,10) | 1.32(0.60,2.58) |  | 3(2,5) | 0.66(0.12,2.38) |  | -3.57(-3.99,-3.15) | -66.85(-77.08,-48.13) |
| Liberia | 65(43,90) | 11.40(8.66,14.95) |  | 114(76,166) | 8.10(6.65,9.80) |  | 0.00(-0.50,0.50) | 75.99(17.73,140.97) |
| Lithuania | 5(4,6) | 0.51(0.16,1.23) |  | 2(1,3) | 0.30(0.03,1.37) |  | -2.83(-3.16,-2.51) | -59.73(-70.29,-34.33) |
| Madagascar | 513(343,699) | 18.13(16.51,19.89) |  | 844(557,1167) | 11.04(10.29,11.85) |  | -1.80(-2.03,-1.56) | 64.65(13.75,137.16) |
| Malawi | 247(162,361) | 10.20(8.90,11.68) |  | 308(196,470) | 5.74(5.08,6.48) |  | -2.17(-2.41,-1.93) | 24.76(-21.68,109.59) |
| Maldives | 1(1,2) | 2.56(0.06,17.89) |  | 1(1,1) | 0.68(0.01,5.28) |  | -4.38(-4.65,-4.11) | -28.77(-56.40,29.60) |
| Mali | 178(119,250) | 9.14(7.81,10.67) |  | 372(240,551) | 6.69(5.99,7.46) |  | 0.00(-0.33,0.34) | 108.94(44.27,189.27) |
| Malta | 0(0,0) | 0.10(0.00,4.93) |  | 0(0,0) | 0.06(0.00,6.07) |  | -1.10(-1.28,-0.92) | -32.53(-44.70,-21.48) |
| Mauritius | 1(0,2) | 0.25(0.00,2.28) |  | 2(1,3) | 0.58(0.06,2.30) |  | 3.27(3.00,3.54) | 150.00(63.37,313.76) |
| Moldova | 14(11,17) | 1.24(0.67,2.12) |  | 6(5,9) | 0.66(0.24,1.59) |  | -2.87(-3.17,-2.57) | -53.37(-66.19,-34.02) |
| Mozambique | 919(646,1285) | 28.20(26.35,30.16) |  | 898(591,1342) | 11.13(10.37,11.93) |  | -3.36(-3.56,-3.16) | -2.29(-31.02,47.67) |
| Namibia | 20(14,30) | 6.04(3.58,9.85) |  | 26(18,40) | 3.96(2.57,5.91) |  | -2.75(-3.50,-1.99) | 29.31(-5.37,76.14) |
| Nauru | 0(0,0) | 5.82(0.00,248.41) |  | 0(0,1) | 12.47(0.00,200.49) |  | 2.07(1.76,2.38) | 154.15(64.84,335.19) |
| Nicaragua | 7(5,9) | 0.78(0.28,1.87) |  | 10(7,13) | 0.53(0.25,1.02) |  | -1.62(-1.86,-1.39) | 45.58(4.41,90.19) |
| Niue | 0(0,0) | 2.88(0.00,849.41) |  | 0(0,0) | 5.89(0.00,1110.04) |  | 1.79(1.55,2.04) | 59.21(-1.16,166.77) |
| Palau | 0(0,0) | 0.94(0.00,131.39) |  | 0(0,0) | 2.90(0.00,139.21) |  | 3.67(3.19,4.14) | 192.72(54.64,392.68) |
| Panama | 6(5,8) | 1.08(0.40,2.48) |  | 10(8,14) | 0.97(0.47,1.79) |  | 0.67(0.25,1.09) | 61.73(25.35,103.99) |
| Paraguay | 23(17,34) | 2.50(1.56,3.86) |  | 50(36,71) | 2.63(1.95,3.50) |  | 1.09(0.81,1.36) | 115.80(62.99,184.92) |
| Peru | 83(62,116) | 1.52(1.20,1.91) |  | 59(36,99) | 0.61(0.46,0.78) |  | -4.44(-4.88,-4.00) | -29.00(-54.03,6.87) |
| Poland | 31(26,37) | 0.32(0.22,0.47) |  | 15(11,20) | 0.15(0.08,0.26) |  | -2.77(-3.05,-2.49) | -53.05(-61.40,-43.12) |
| Rwanda | 249(154,371) | 14.39(12.57,16.46) |  | 177(115,310) | 4.84(4.14,5.64) |  | -4.65(-5.08,-4.22) | -29.05(-56.56,26.04) |
| San Marino | 0(0,0) | 0.21(0.00,64.89) |  | 0(0,0) | 0.18(0.00,65.57) |  | -0.30(-0.46,-0.15) | -2.73(-44.01,89.76) |
| Senegal | 43(31,60) | 2.48(1.77,3.45) |  | 55(38,80) | 1.39(1.04,1.84) |  | -3.00(-3.39,-2.61) | 26.67(-6.87,73.64) |
| Serbia | 4(3,6) | 0.17(0.05,0.44) |  | 2(1,4) | 0.10(0.01,0.39) |  | -1.84(-1.96,-1.72) | -45.51(-60.76,-22.83) |
| Seychelles | 0(0,1) | 2.40(0.00,37.21) |  | 0(0,0) | 1.34(0.00,19.37) |  | -1.25(-2.13,-0.37) | -24.09(-48.13,28.39) |
| Sierra Leone | 32(23,45) | 3.06(2.04,4.53) |  | 55(38,78) | 2.38(1.77,3.18) |  | -0.87(-0.98,-0.76) | 75.44(41.85,116.76) |
| Singapore | 2(1,3) | 0.23(0.03,0.87) |  | 3(2,5) | 0.20(0.04,0.81) |  | -0.58(-0.74,-0.42) | 46.62(13.28,84.66) |
| Slovenia | 1(1,1) | 0.20(0.00,1.18) |  | 0(0,1) | 0.09(0.00,1.44) |  | -2.35(-2.53,-2.16) | -59.75(-73.06,-39.86) |
| South Africa | 1077(723,1477) | 11.34(10.65,12.06) |  | 923(650,1307) | 5.92(5.54,6.32) |  | -2.64(-3.18,-2.09) | -14.33(-33.22,36.94) |
| South Sudan | 193(129,302) | 14.24(12.18,16.65) |  | 319(210,532) | 13.35(11.87,14.98) |  | 0.01(-0.19,0.21) | 65.26(18.09,139.39) |
| Sudan | 93(63,134) | 1.99(1.59,2.47) |  | 129(82,199) | 1.14(0.95,1.37) |  | -1.29(-1.72,-0.85) | 38.22(18.07,66.24) |
| Suriname | 1(1,1) | 0.98(0.02,6.79) |  | 2(1,3) | 1.27(0.13,4.87) |  | 0.33(0.12,0.54) | 102.31(42.22,181.73) |
| Tajikistan | 17(10,28) | 1.49(0.82,2.64) |  | 18(8,41) | 0.70(0.41,1.14) |  | -3.25(-3.51,-2.99) | 3.69(-63.79,160.38) |
| Congo (Brazzaville) | 46(31,64) | 8.46(6.06,11.64) |  | 73(46,105) | 5.12(4.00,6.48) |  | -1.92(-2.14,-1.69) | 59.05(3.90,130.63) |
| The Gambia | 11(7,16) | 4.51(2.08,9.46) |  | 25(15,36) | 3.93(2.48,6.13) |  | -1.14(-1.39,-0.88) | 132.73(51.26,252.98) |
| Marshall Islands | 0(0,1) | 4.21(0.00,90.43) |  | 1(1,2) | 6.78(0.17,41.55) |  | 1.40(1.11,1.69) | 150.43(63.84,296.80) |
| Niger | 34(23,52) | 1.97(1.34,2.84) |  | 56(37,99) | 1.06(0.79,1.42) |  | -3.05(-3.48,-2.61) | 63.88(21.21,135.12) |
| Philippines | 129(82,203) | 0.84(0.69,1.00) |  | 562(370,805) | 1.92(1.76,2.09) |  | 3.43(3.15,3.72) | 335.55(229.56,441.23) |
| Myanmar | 177(121,288) | 1.69(1.44,1.97) |  | 186(137,252) | 1.23(1.06,1.42) |  | -0.77(-0.94,-0.61) | 4.92(-23.13,36.87) |
| Trinidad and Tobago | 3(2,3) | 0.90(0.15,3.11) |  | 4(3,5) | 1.04(0.26,2.94) |  | 0.35(-0.51,1.21) | 40.19(4.44,86.77) |
| Tunisia | 3(1,6) | 0.13(0.02,0.48) |  | 4(1,8) | 0.11(0.03,0.33) |  | -0.25(-0.34,-0.15) | 39.15(-9.64,125.34) |
| Turkey | 46(27,81) | 0.32(0.23,0.44) |  | 39(17,84) | 0.18(0.13,0.25) |  | -1.72(-2.12,-1.32) | -14.13(-49.83,23.74) |
| Uganda | 354(225,521) | 8.77(7.81,9.84) |  | 1141(785,1584) | 10.37(9.74,11.03) |  | 0.29(-0.05,0.63) | 222.07(113.34,372.44) |
| Uzbekistan | 40(31,50) | 0.84(0.58,1.21) |  | 23(17,31) | 0.26(0.16,0.39) |  | -4.60(-5.35,-3.85) | -42.51(-59.73,-16.41) |
| Vanuatu | 1(1,2) | 3.87(0.17,24.02) |  | 4(2,6) | 5.24(1.42,14.24) |  | 0.94(0.83,1.06) | 205.86(102.77,416.71) |
| Yemen | 11(7,16) | 0.40(0.19,0.79) |  | 26(17,38) | 0.31(0.20,0.47) |  | -1.11(-1.22,-1.00) | 135.12(105.09,177.85) |
| Zambia | 326(212,455) | 16.67(14.77,18.78) |  | 489(295,831) | 9.38(8.53,10.31) |  | -2.42(-2.74,-2.10) | 49.83(-9.90,187.14) |
| Zimbabwe | 115(75,187) | 4.89(3.98,5.99) |  | 211(127,337) | 5.25(4.55,6.04) |  | 1.00(0.57,1.44) | 83.44(23.06,163.25) |
| Romania | 13(10,20) | 0.24(0.13,0.42) |  | 6(4,11) | 0.15(0.05,0.34) |  | -1.07(-1.27,-0.88) | -53.09(-66.77,-33.75) |
| Russia | 336(314,380) | 0.90(0.81,1.01) |  | 290(246,352) | 0.76(0.68,0.86) |  | -1.36(-2.08,-0.63) | -13.51(-24.92,-1.97) |
| Saint Kitts and Nevis | 0(0,1) | 3.99(0.00,84.87) |  | 0(0,0) | 2.18(0.00,31.52) |  | -1.31(-1.73,-0.88) | -9.87(-33.05,20.22) |
| Saint Lucia | 1(1,1) | 2.32(0.01,22.32) |  | 2(1,2) | 3.23(0.23,15.17) |  | 1.86(1.52,2.20) | 108.43(66.87,164.85) |
| Saint Vincent and the Grenadines | 0(0,0) | 0.77(0.00,27.63) |  | 0(0,0) | 0.92(0.00,16.60) |  | 0.94(0.63,1.25) | 35.30(11.98,70.25) |
| Slovakia | 4(3,5) | 0.29(0.07,0.77) |  | 3(2,4) | 0.18(0.03,0.72) |  | -1.28(-1.42,-1.13) | -32.77(-52.62,4.65) |
| Viet Nam | 52(36,80) | 0.32(0.23,0.43) |  | 53(35,81) | 0.20(0.15,0.26) |  | -1.59(-1.73,-1.46) | 1.63(-21.92,28.25) |
| Solomon Islands | 4(3,6) | 5.64(1.39,16.99) |  | 9(5,13) | 5.06(2.23,10.08) |  | -0.37(-0.69,-0.03) | 110.12(38.18,212.44) |
| Eritrea | 76(45,160) | 9.35(7.29,11.89) |  | 98(60,184) | 5.73(4.63,7.05) |  | -1.56(-1.71,-1.42) | 29.33(-22.02,118.85) |
| Israel | 1(1,2) | 0.09(0.00,0.58) |  | 1(1,2) | 0.06(0.00,0.29) |  | -1.32(-1.50,-1.14) | 17.45(-6.72,41.82) |
| Kuwait | 0(0,1) | 0.11(0.00,2.01) |  | 4(2,6) | 0.23(0.05,0.81) |  | 2.78(2.30,3.27) | 664.05(348.03,1570.57) |
| Libya | 1(0,3) | 0.13(0.00,0.97) |  | 3(2,6) | 0.16(0.03,0.46) |  | 0.39(0.15,0.63) | 176.28(70.91,411.47) |
| Qatar | 0(0,1) | 0.51(0.00,10.00) |  | 2(1,3) | 0.29(0.02,1.58) |  | -1.82(-2.05,-1.60) | 306.24(152.73,532.48) |
| Oman | 0(0,1) | 0.11(0.00,2.05) |  | 1(0,1) | 0.07(0.00,0.62) |  | -1.64(-1.86,-1.43) | 94.55(21.40,185.65) |
| Switzerland | 3(3,4) | 0.18(0.04,0.56) |  | 2(1,3) | 0.09(0.01,0.42) |  | -2.34(-2.50,-2.17) | -41.34(-56.10,-19.21) |
| Syria | 38(24,55) | 1.43(0.99,2.04) |  | 24(14,50) | 0.68(0.42,1.06) |  | -2.81(-3.45,-2.16) | -35.88(-64.58,26.52) |
| Taiwan (province of China) | 14(10,21) | 0.26(0.14,0.46) |  | 13(10,20) | 0.21(0.11,0.39) |  | -0.26(-0.59,0.07) | -3.88(-22.75,16.54) |
| Togo | 32(20,47) | 3.71(2.49,5.45) |  | 53(37,77) | 2.44(1.82,3.23) |  | -3.24(-3.88,-2.59) | 65.71(15.35,128.19) |
| Tokelau | 0(0,0) | 8.71(0.00,1470.77) |  | 0(0,0) | 7.50(0.00,1319.01) |  | -0.59(-0.79,-0.40) | -21.69(-53.39,55.99) |
| Turkmenistan | 11(9,13) | 1.26(0.59,2.52) |  | 7(5,10) | 0.59(0.25,1.21) |  | -2.87(-3.56,-2.17) | -31.41(-52.22,-1.66) |
| Tuvalu | 0(0,0) | 5.20(0.00,192.40) |  | 0(0,0) | 6.85(0.00,167.87) |  | 0.34(-0.36,1.03) | 58.32(-4.49,177.08) |
| Ukraine | 55(46,65) | 0.43(0.32,0.56) |  | 56(34,88) | 0.49(0.37,0.66) |  | 0.00(-0.53,0.53) | 2.98(-39.85,64.94) |
| Comoros | 15(9,24) | 13.37(7.21,23.65) |  | 14(9,23) | 6.75(3.63,11.66) |  | -3.56(-4.27,-2.85) | -8.06(-45.56,53.60) |
| United Arab Emirates | 2(1,5) | 0.68(0.09,3.54) |  | 5(3,7) | 0.34(0.09,0.93) |  | -2.48(-2.99,-1.96) | 111.35(5.39,327.35) |
| UK | 33(27,45) | 0.23(0.16,0.33) |  | 32(24,45) | 0.20(0.14,0.29) |  | -0.45(-0.73,-0.17) | -4.18(-12.82,4.75) |
| Mexico | 293(257,348) | 1.43(1.26,1.61) |  | 500(393,692) | 1.43(1.30,1.56) |  | 1.13(0.36,1.91) | 70.47(35.89,131.78) |
| Tanzania | 887(560,1229) | 13.94(12.98,14.96) |  | 1016(647,1527) | 6.52(6.12,6.96) |  | -3.50(-3.89,-3.10) | 14.52(-27.51,89.36) |
| USA | 290(217,411) | 0.43(0.38,0.48) |  | 258(184,386) | 0.33(0.29,0.38) |  | -0.89(-0.99,-0.79) | -11.17(-17.07,-4.91) |
| Virgin Islands | 0(0,1) | 1.12(0.00,16.61) |  | 0(0,0) | 1.46(0.00,29.50) |  | 0.81(0.50,1.14) | -21.16(-54.98,34.75) |

ASR, age standardized rate; CI, confidence interval; EAPC, estimated annual percentage change; UI, uncertainty interval; SDI, socio-demographic index.
